# Synergistic Spin‐Polarization and Single‐Atom Engineering in Magnetic Heterojunctions for Efficient Solar Water Splitting

**DOI:** 10.1002/advs.202524114

**Published:** 2026-01-21

**Authors:** Hongyang Ren, Zhenzhou Guo, Huirong Wu, Peilin Huang, Jie Yang, Jie Zhang, Guangqian Ding, Hongkuan Yuan, Biao Wang

**Affiliations:** ^1^ School of Physical Science and Technology Southwest University Chongqing China; ^2^ Institute for Superconducting and Electronic Materials, Faculty of Engineering and Information Sciences University of Wollongong Wollongong Australia; ^3^ School of Electronic Science and Engineering Chongqing University of Posts and Telecommunications Chongqing China; ^4^ Yibin Academy of Southwest University Yibin Sichuan China

**Keywords:** high‐throughput screening, single‐atom anchoring, spin polarization, solar water splitting, Z‐scheme heterojunction

## Abstract

The advancement of photocatalytic water splitting requires going beyond charge migration control to the greater challenge of utilizing the spin degree of freedom as a handle to precisely steer reaction pathways. Here, it is identified 67 magnetic heterojunctions composed of magnetic 2D transition metal halide and non‐magnetic transition metal chalcogenide monolayers via high‐throughput screening. These selected structures exhibit a lattice mismatch below 5%, with each constituent monolayer possessing a band gap in the optimal range of 0.5 – 2.5 eV. First‐principles calculations confirm a type‐II staggered band alignment and a built‐in electric field that promotes efficient charge separation. More importantly, the unique spin‐polarized electronic configuration of Cr^3+^ in CrI_3_ preferentially facilitates the generation of triplet oxygen, significantly boosting the oxygen evolution reaction. Meanwhile, the anchoring of Pt single atoms on the MoTe_2_ and WTe_2_ layers addresses their weak hydrogen adsorption, enhancing the HER kinetics to balance the overall water splitting process. This synergistic engineering of spin‐polarization for OER and single‐atom sites for HER works cooperatively within the heterojunction. Together with strong visible‐light absorption and a predicted solar‐to‐hydrogen efficiency that surpasses industrial benchmarks, this study highlights a high‐throughput‐guided strategy for designing high‐performance magnetic photocatalysts through multi‐component active site optimization.

## Graphical abstract

High‐throughput screening led to the identification of 67 Z‐scheme heterojunctions (comprising 2D magnetic transition metal halides and non‐magnetic transition metal chalcogenides). For CrI_3_/MoTe_2_ and CrI_3_/WTe_2_, electronic structure analysis demonstrated that synergistic crystallographic point group and built‐in electric field effects generate a distinct electronic configuration of Cr^3+^ ions favorable for oxygen evolution reaction (OER). Spin polarization's OER‐enhancing role is elucidated via collinear and non‐collinear magnetic calculations. Single‐atom anchoring on the heterojunctions improved hydrogen evolution and light absorption, yielding theoretical solar‐to‐hydrogen (STH) conversion efficiencies of 16.97% and 15.61%.

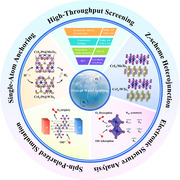

## 1 Introduction

With the escalating challenges of global warming and environmental pollution caused by the consumption of fossil fuels, increasing attention has been paid to the development of sustainable and clean energy sources [1]. Since the seminal discovery by Fujishima and Honda in 1972 that ultraviolet irradiation could induce water splitting on a TiO_2_ electrode, solar‐driven photocatalytic water splitting has emerged as a promising strategy for sustainable hydrogen production, offering a viable solution to global energy challenges [2]. Many 2D semiconductor materials, compared to bulk materials, exhibit a higher surface‐to‐volume ratio, significant carrier mobility, and suitable band edge positions, making them promising candidates for photocatalysts [3, 4, 5]. Additionally, the integration of 2D materials with suitable band alignment into van der Waals (vdW) heterojunctions (HJs) demonstrates enhanced photocatalytic performance through optimized light harvesting and efficient separation of photogenerated charge carriers [6, 7, 8]. Particularly, Z‐scheme heterojunctions, which mimic natural photosynthetic systems, enabling the hydrogen evolution reaction (HER) and oxygen evolution reaction (OER) to occur on different materials [9, 10, 11]. This configuration not only expands the selection scope for narrow‐bandgap semiconductors but also establishes interfacial built‐in electric fields through charge redistribution. These electric fields synergistically facilitate carrier separation while suppressing recombination, thereby boosting overall photocatalytic efficiency through dual enhancement mechanisms.

Electrons are fundamentally characterized by two intrinsic degrees of freedom: charge and spin [12]. While the built‐in electric field in heterojunctions exclusively modulates the charge degree of freedom to enhance carrier mobility, the effective manipulation of electron spin degree of freedom has emerged as a pivotal strategy for optimizing photocatalytic efficiency [13, 14, 15]. This spin control mechanism operates through two complementary aspects: individual electron spin orientation, which represents the intrinsic angular momentum manifested through spin‐up or spin‐down states, and collective spin configurations in multi‐electron systems, as quantified by singlet, doublet, triplet, and other multiplicity states within atomic orbitals [16, 17, 18, 19, 20]. Substantial evidence demonstrates that strategic spin engineering can significantly enhance photocatalytic performance. B. D. Ravetz et al. demonstrated that electron spin modulation enables band structure tailoring in photocatalysts, effectively broadening, expanding their absorption spectrum [21]. L. Pan et al. demonstrated that introducing Ti defects in bulk TiO_2_ induces spin polarization in the material, where parallel alignment of electron spins facilitates synergistic enhancement of charge separation efficiency and surface reaction kinetics [22]. Similarly, Y. Li et al., improved OER activity in Co_3_O_4_ through cobalt defect‐mediated spin polarization, which promotes triplet oxygen generation and reduces the Gibbs free energy barrier (ΔG) of rate‐determining steps [23]. External magnetic fields present a non‐invasive approach for dynamic spin manipulation, enabling real‐time modulation of catalytic activity and selectivity without structural modification [24, 25, 26]. T. Sun et al. developed ferromagnetic Ni_1_/MoS_2_ single‐atom catalysts exhibiting remarkable magnetoelectric effects, where spin coupling enables an order‐of‐magnitude current density increase under 502 mT magnetic field during water splitting [27].

Previous studies have primarily focused on defect engineering strategies to induce spin polarization, which inevitably compromises the intrinsic structural integrity of host materials. Moreover, the current hydrogen production efficiency of photocatalytic systems remains relatively low, still falling far short of industrial application requirements.

**FIGURE 1:**
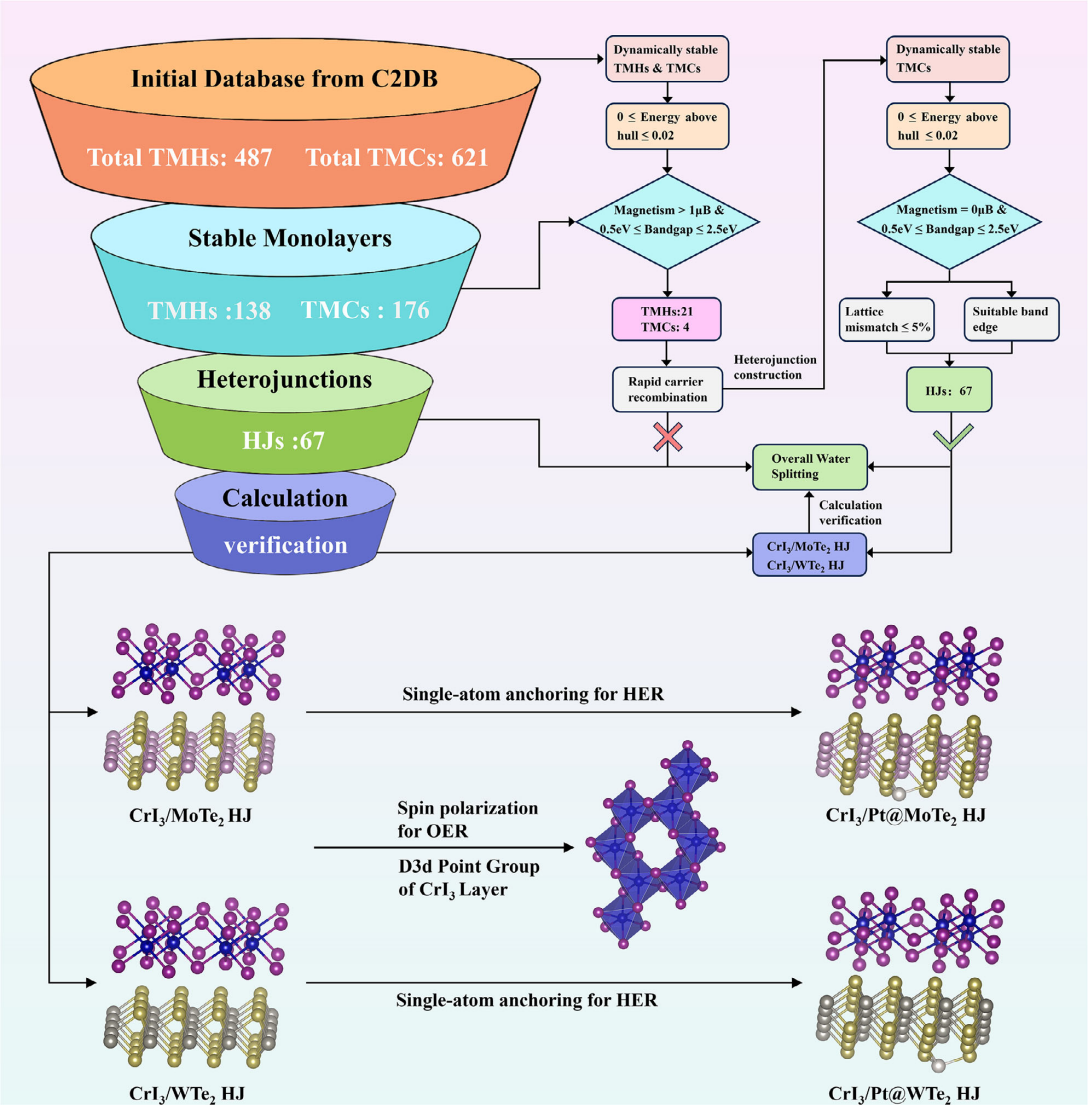
The main process of the high‐throughput screening and simplified diagram of the subsequent material analysis.

In this work, high‐throughput screening of the C2DB database for transition metal chalcogenides (TMCs) and halides (TMHs) led to the identification of potential magnetic monolayers as catalyst components for high‐performance photocatalytic hydrogen production [28, 29]. Through these magnetic TMHs materials, we have also identified several systems exhibiting fractional quantum ferroelectricity (FQFE). Selected magnetic monolayers were paired with non‐magnetic TMCs, as schematically illustrated in Figure 1, ultimately establishing 67 optimized HJs to address high carrier recombination in monolayers. The CrI_3_/MoTe_2_ and CrI_3_/WTe_2_ HJs were prioritized due to their exceptional magnetic moments (3 µB for CrI_3_) [30, 31] and favorable band alignment. These systems combine CrI_3_’s OER activity with MoTe_2_ and WTe_2_’s HER capabilities, while leveraging their complementary light absorption and charge transport properties [32, 33, 34]. Through ab initio molecular dynamics (AIMD) simulations and hybrid density functional theory calculations, we comprehensively analyzed their stability, band structure, optical properties, and spin polarization photocatalytic mechanism of both CrI_3_/MoTe_2_ and CrI_3_/WTe_2_ HJs. While their band edges are thermodynamically favorable for HER, both TMCs exhibit weak H^+^ adsorption. Therefore, we further modified these systems by anchoring single Pt atoms to enhance their HER performance. The synergistic combination of built‐in electric fields and spin polarization control in these architectures demonstrates theoretically superior photocatalytic performance.

## 2 Results and Discussion

### 2.1 High‐Throughput Screening

It is well known that transition metal chalcogenides and transition metal halides contain many experimentally confirmed classical semiconductor materials and magnetic materials. However, finding materials that simultaneously possess both semiconducting and magnetic properties requires significant time investment. Therefore, we conducted high‐throughput screening through an API of the C2DB database, targeting materials with chemical compositions ranging from 1:1 to 1:4 stoichiometries in TMHs and TMCs [28, 29]. This initial screening yielded 487 TMHs and 621 TMCs, as shown in Tables S1 and S2. Subsequently, we applied the second screening based on dynamic stability and energy above hull (ranging from 0 to 0.2 eV), resulting in 138 dynamically and thermally stable TMHs and 176 stable TMCs. In the third step, we screened for materials with a total magnetic moment greater than 1 µB and a Heyd‐Scuseria‐Ernzerhof (HSE06) hybrid functional band gap between 0.5 and 2.5 eV. This screening identified 21 TMHs meeting these criteria, as shown in Table S3, but only four suitable TMC with appreciable magnetism and a suitable band gap. Therefore, we selected TMHs as the magnetic component due to their greater quantity and larger magnetic moments. Due to the high carrier recombination rate in monolayers, we chose to construct a Z‐type heterojunction to reduce the carrier recombination rate, in order to achieve efficient water splitting.

**FIGURE 2:**
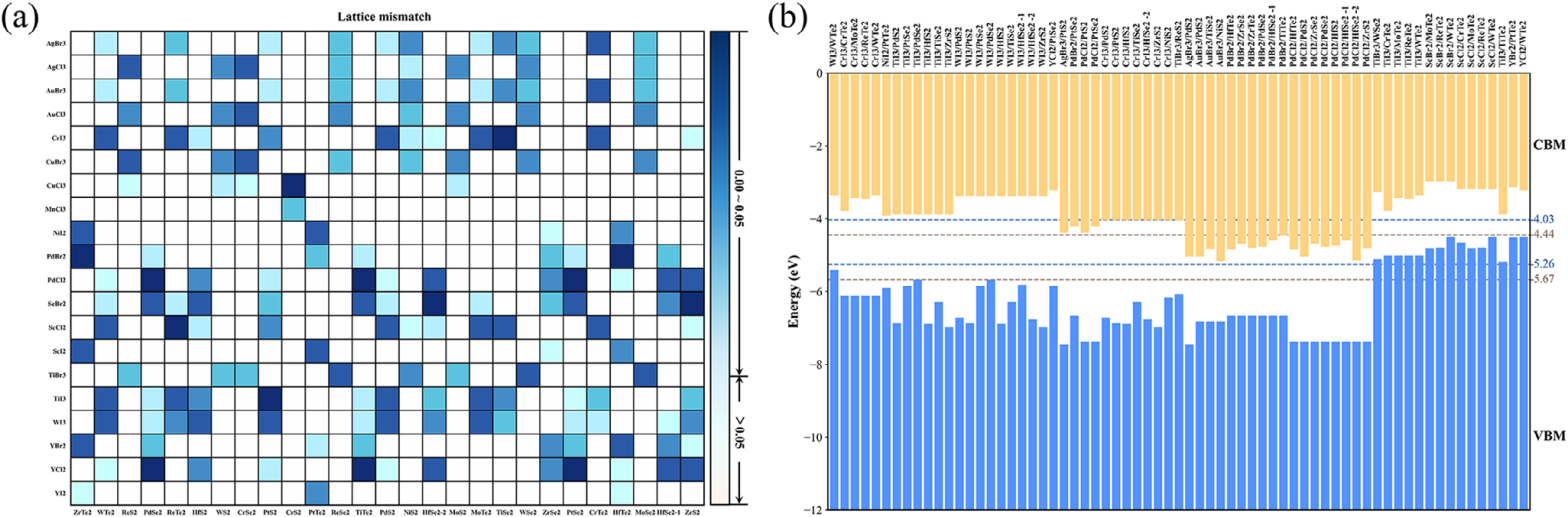
All potential heterojunctions are shown in (a), where the color transition from dark blue to white signifies an increase in lattice mismatch from 0 to values of 0.05 and above. Band edge positions of heterojunctions satisfying Z‐scheme band alignment relative to the redox potentials of water are presented in (b). The dashed lines indicate the redox potentials of water at different pH levels. The orange and blue regions indicate the Conduction Band Minimum (CBM) for the hydrogen evolution material and the Valence Band Maximum (VBM) for the oxygen evolution material in the heterojunction. Overall water splitting can only proceed if the CBM of the hydrogen evolution material exceeds the H^+^/H_2_ reduction potential and the VBM of the oxygen evolution material exceeds the O_2_/H_2_O oxidation potential.

To satisfy the lattice mismatch conditions more effectively, we excluded CuF_2_ from the dataset, as it is the only transition metal halide (TMH) with a rectangular unit cell, unlike the standard rhombus configuration common to the others. This allowed the remaining 20 TMHs to be used for heterojunction construction. Considering that magnetic interactions between two magnetic monolayers may lead to significant changes in magnetic properties, we modified the screening criteria in the subsequent selection process to focus on non‐magnetic semiconductors. Previous screening results indicated that many TMHs exhibit intrinsic magnetism; therefore, we narrowed the scope to TMCs. Furthermore, the construction of heterojunctions requires the lattice mismatch less than 5% between two monolayers and band edge alignment consistent with the Z‐scheme mechanism. Based on these constraints, 67 HJs meeting the criteria were identified. Figure 2a,b presents the potentially lattice‐matched heterojunctions, along with their band edge positions relative to the redox potentials of water under different pH conditions. From these candidates, we finally selected CrI_3_/MoTe_2_ and CrI_3_/WTe_2_ HJs which exhibit considerable magnetic moments and suitable band alignments for overall water splitting across various conditions. These systems were further subjected to detailed computational studies to validate our findings and to investigate the influence of magnetism on the water‐splitting performance.

### 2.2 Structural Stability

The optimized lattice parameters of the MoTe_2_ monolayer (ML), WTe_2_ ML and CrI_3_ ML are 3.56 Å, 3.52 Å and 6.96 Å, respectively, consistent with values reported in previous studies [30, 35]. The band structures for these monolayers were calculated using the HSE06 hybrid functional. As shown in Figure S2, the MoTe_2_ ML exhibits a direct bandgap of 1.63 eV, the WTe_2_ ML shows a direct bandgap of 1.66 eV, similarly the CrI_3_ ML is also a direct semiconductor with a bandgap of 1.90 eV. The obtained values are consistent with those reported in previous literature [36, 37]. The lattice mismatch ratio was calculated using the formula: 2|*a_A_* − *a_B_* |/(*a_A_* + *a_B_*)  ×  100%, where *a_A_* and *a_B_* represent the lattice parameters of the CrI_3_ ML and MoTe_2_ ML (WTe_2_ ML), respectively. A 1×1 unit cell of CrI_3_ ML and a 2 × 2 supercell of MoTe_2_ ML (WTe_2_ ML) were employed to construct CrI_3_/MoTe_2_ HJ (CrI_3_/WTe_2_ HJ), resulting in lattice mismatches of 2.27% and 1.14%, respectively.

**FIGURE 3:**
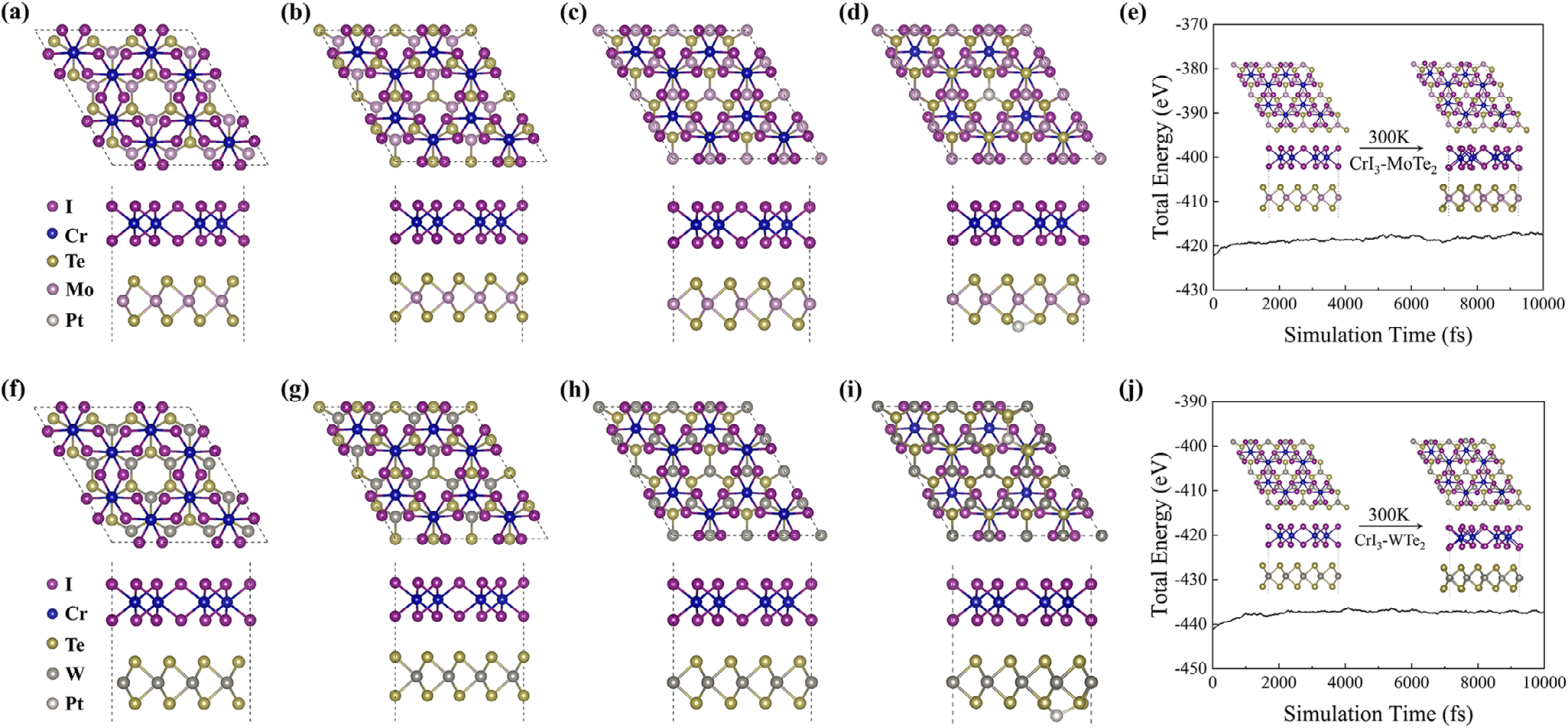
Top and side views of stacking configurations for CrI_3_/MoTe_2_ heterostructures: (a) stacking‐1, (b) stacking‐2, and (c) stacking‐3; and for CrI_3_/WTe_2_ heterostructures: (f) stacking‐4, (g) stacking‐5, and (h) stacking‐6. Also shown are the top and side views of (d) CrI_3_/Pt@MoTe_2_ and (i) CrI_3_/Pt@WTe_2_. Ab initio molecular dynamics simulation results at 300 K are presented for (e) CrI_3_/MoTe_2_ and (j) CrI_3_/WTe_2_ heterojunctions.

To preliminarily evaluate the most stable configuration of these HJs, we calculated their binding energy using the formula:

(1) $$E_{b}=\left[E_{\left(A/B\right)}-E_{A}-E_{B}\right]$$

where *E_A_* and *E_B_* represent the energies of the isolated CrI_3_ ML and MoTe_2_ ML (WTe_2_ ML), respectively, and *E*_(*A*/*B*)_ denotes the total energy of the formed CrI_3_/MoTe_2_ HJ (CrI_3_/WTe_2_ HJ) [38]. We designed three distinct stacking configurations of CrI_3_/MoTe_2_ HJs, labeled as Stacking‐1 to Stacking‐3 (Figure 3a–c), with corresponding binding energies of −0.76, −0.67, and −0.78 eV, respectively. Similarly, three CrI_3_/WTe_2_ HJs were designed and designated as Stacking‐4 to Stacking‐6 (Figure 3f–h), exhibiting binding energies of −0.78, −0.69, and −0.80 eV, respectively. The negative values confirm the thermal stability of all configurations, with more negative values indicating higher stability. Consequently, we selected the Stacking‐3 configuration of CrI_3_/MoTe_2_ HJ and the Stacking‐6 configuration of CrI_3_/WTe_2_ HJ for further analysis, as thy exhibit the lowest binding energies.

After structural optimization, both Stacking‐3 and Stacking‐6 configurations exhibited characteristic van der Waals (vdW) interlayer spacing and ferromagnetism, with the magnetic moments originating exclusively from the CrI_3_ layer. To further assess the magnetic ground state, we performed comparative antiferromagnetic simulations. The results indicate that the total energies of both CrI_3_/MoTe_2_ and CrI_3_/WTe_2_ HJs in their antiferromagnetic configurations are higher than those in the ferromagnetic state, confirming the stability of their intrinsic ferromagnetism. To evaluate their dynamical stability, phonon dispersion calculations were performed for these HJs. As shown in Figure S3, all systems demonstrate real‐frequency phonon modes without imaginary frequencies, indicating dynamical stability. To exam thermal stability, we constructed 2 × 2 × 1 supercells for both HJs and performed ab initio molecular dynamics (AIMD) simulations at 300 K. As shown in Figure 3e,j, the total energy converges to a stable range after 10 000 steps with a 1‐fs timestep, demonstrating the stability of both HJs under room temperature. Considering the influence of different stacking configurations on the electronic structure, we computed the projected band structures for the six stacking configurations mentioned above, as shown in Figure S4. It can be observed that the overall band arrangements are quite similar across these stackings. Therefore, in the subsequent study, we focus on a detailed investigation of the electronic structures of stacking‐3 and stacking‐6, which are the most stable configurations.

### 2.3 Electronic Structure and Orbital Analysis

**FIGURE 4:**
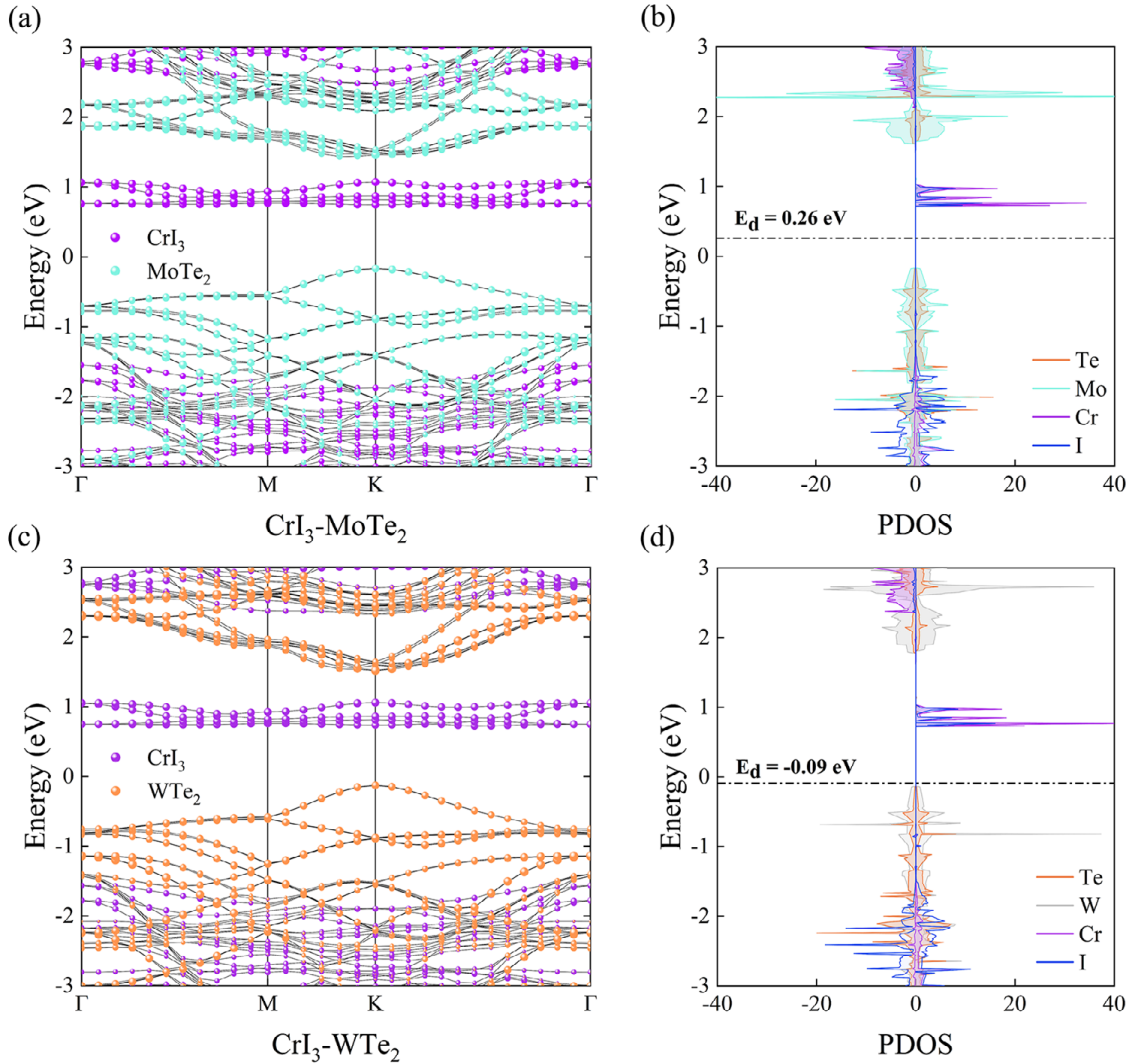
The projected band structure of CrI_3_/MoTe_2_ HJ (a) and CrI_3_/WTe_2_ HJ (c) and density of states of CrI_3_/WTe_2_ HJ (b) and CrI_3_/WTe_2_ HJ (d). The relative sizes of the cyan (orange) and purple dots correspond to the weight contributions of MoTe_2_ (WTe_2_) and CrI_3_, respectively. The Fermi level is defined as the energy reference point at zero energy. E_d_ is the d‐band center.

As shown in Figure 4a,c, the projected band structure of the CrI_3_/MoTe_2_ HJ exhibits an indirect bandgap of 0.9 eV. The conduction band minimum (CBM) lies between the K and Γ points, while the valence band maximum (VBM) is located at K point. A similar band alignment is observed in the CrI_3_/WTe_2_ HJ, with the CBM also positioned between K and Γ points, and the VBM remaining at K point. This system shows a slightly reduced bandgap of 0.85 eV. According to the projected density of states (PDOS) analysis illustrated in Figure 4b,d, the CBM of the CrI_3_/MoTe_2_ HJ is mostly contributed by CrI_3_, while the VBM originates mainly from MoTe_2_. Similarly, in the CrI_3_/WTe_2_ HJ, the CBM is primarily attributed to CrI_3_, whereas the VBM is chiefly from WTe_2_. Both HJs exhibit a characteristic type‐II band alignment, which facilitates the effective separation of photogenerated electron‐hole pairs. The magnetism in both systems is evidenced by the asymmetric distribution of spin‐up and spin‐down electrons in the PDOS. Furthermore, calculations reveal that the d‐band centers of CrI_3_/MoTe_2_ and CrI_3_/WTe_2_ HJ are positioned at 0.26 eV and −0.09 eV, respectively. Both d‐band centers are close to the Fermi level, indicating their strong catalytic activities [39].

**FIGURE 5:**
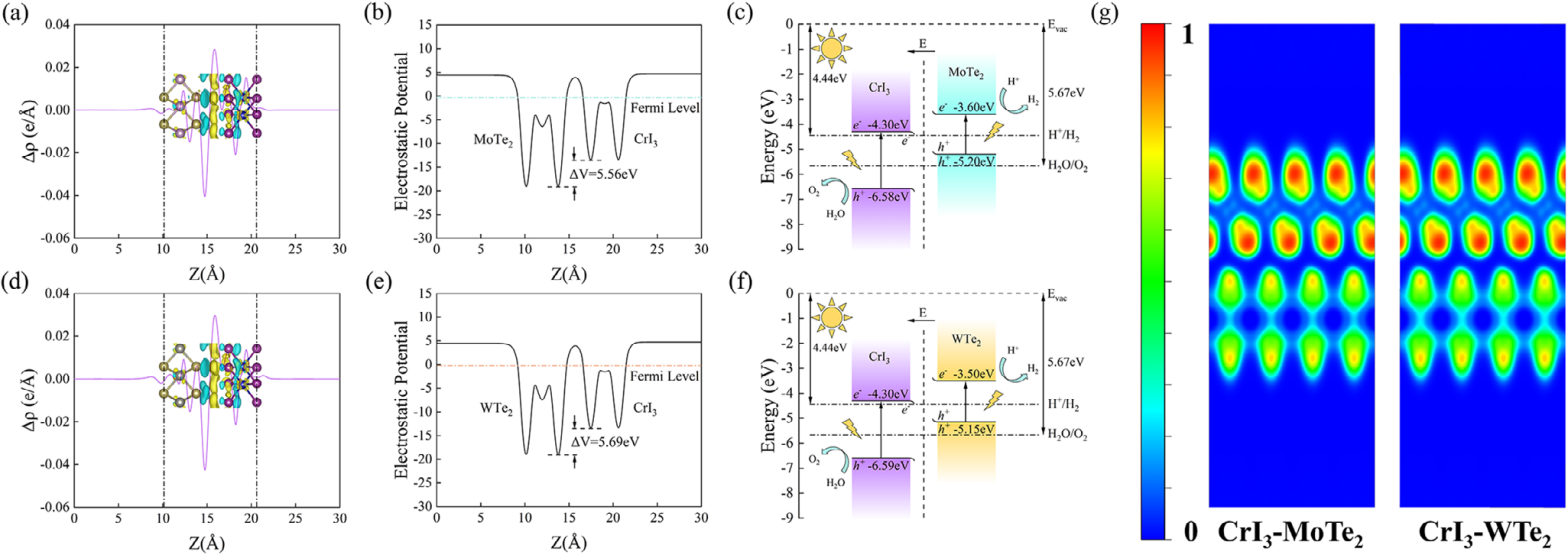
Planar‐averaged charge density and charge density difference for the CrI_3_/MoTe_2_ HJ (a) and the CrI_3_/WTe_2_ HJ (d). Yellow regions correspond to areas of charge accumulation, while cyan indicates regions of charge depletion. The purple curve depicts the planar‐averaged charge density difference, with positive (negative) values denoting charge accumulation (depletion). Electrostatic potential of the CrI_3_/MoTe_2_ HJ (b) and the CrI_3_/WTe_2_ HJ (e), where red and cyan dashed line indicates the Fermi level. Band edge arrangement and charge transfer mechanism for the CrI_3_/MoTe_2_ HJ (c) and the CrI_3_/WTe_2_ HJ (f) with respect to vacuum level. Electron localization function (ELF) distributions of CrI_3_/MoTe_2_ and CrI_3_/WTe_2_ HJs are presented in Figure 5g.

Through charge density difference analysis, we systematically investigated the interlayer charge transfer and redistribution along the Z‐axis in CrI_3_/MoTe_2_ HJ and CrI_3_/WTe_2_ HJ. The charge variation was quantitatively evaluated using the following computational approach:

(2) $$\Delta \;\rho_{\left(Z\right)}=\rho_{A/B\left(Z\right)}\;-\;\rho_{A\left(Z\right)}-\;\rho_{B\left(Z\right)}$$

where *ρ*_*A*/*B*(*Z*)_, *ρ*_*A*(*Z*)_, and *ρ*_*B*(*Z*)_, represent the charge densities of the CrI_3_/MoTe_2_ HJ (CrI_3_/WTe_2_ HJ), the MoTe_2_ ML (WTe_2_ ML), and the CrI_3_ ML, respectively. Regions with positive *Δρ*_(*Z*)_ values correspond to electron accumulation, while negative values indicate electron depletion. As illustrated in Figure 5a,d, charge redistribution occurs predominantly at the interface, which leads to electron depletion in the MoTe_2_ layer (WTe_2_ layer) and accumulation in the CrI_3_ layer. Bader charge analysis further reveals a transfer of 0.054 *e* from MoTe_2_ to CrI_3_ in the CrI_3_/MoTe_2_ HJ, while a migration of 0.058 *e* occurs from WTe_2_ to CrI_3_ in the CrI_3_/WTe_2_ HJ. These results demonstrate similar interfacial charge separation, with electrons accumulating preferentially in the CrI_3_ layer and holes localizing in the MoTe_2_ (or WTe_2_) layer, matching the charge density difference analysis. Consequently, built‐in electric fields are established across the HJs, oriented from the MoTe_2_ (or WTe_2_) layer toward the CrI_3_ layer. The built‐in electric field typically induces distinct electrostatic potentials on the two surfaces of the HJs. The planar averaged electrostatic potential is given by the following formula [40]:

(3) $$V\;\left(z\right)=\frac{1}{S}\;\int_{s}V\left(r\right)\mathrm{dxdy}$$

where *S* denotes the contact area of the xy‐plane and *V*(*r*) represents the electrostatic potential at any points. Furthermore, the work function (Φ) is also instrumental in elucidating the dynamics of charge transfer and redistribution at interfaces.

(4) $$\Phi =E_{vac}-E_{F}$$

Where *E_vac_* and *E_F_* denote the vacuum level and Fermi level, respectively. The electrostatic potentials and work functions of the MoTe_2_, WTe_2_, and CrI_3_ MLs are summarized in Figure S5. The calculated work functions (*Φ*) are 4.60 eV for MoTe_2_, 4.45 eV for WTe_2_, and 5.58 eV for CrI_3_, respectively. When two semiconductors possessing distinct work functions form a heterojunction, electrons transfer from the material with lower work function to that with higher work function until their Fermi level alignment is achieved. The vertical electrostatic potential of the CrI_3_/MoTe_2_ and CrI_3_/WTe_2_ HJs are presented in Figure 5b,e, respectively, which clearly demonstrates the existence of built‐in electric fields through the observed potential drop values. Consequently, in the CrI_3_/MoTe_2_ HJ, electrons transfer from the MoTe_2_ ML to the CrI_3_ ML, while in the CrI_3_/WTe_2_ HJ, charge transfer occurs from the WTe_2_ ML to the CrI_3_ ML until their Fermi levels reach equilibrium, accompanied by interfacial charge redistribution. These findings are consistent with the charge density difference analysis. Furthermore, Figure 5g presents the electron localization function (ELF) distributions of the two HJs. In the interlayer region between CrI_3_ and MoTe_2_ (WTe_2_), the ELF values approach zero, indicating a highly delocalized electronic state that allows electrons move freely. This observation suggests the absence of chemical bonding between CrI_3_ and MoTe_2_ (WTe_2_), with interactions governed solely by van der Waals. This results in efficient charge separation capabilities.

Based on the aforementioned calculations, the band edge positions of the CrI_3_/MoTe_2_ HJ and CrI_3_/WTe_2_ HJ relative to the vacuum level are presented in Figure 5c,f. At pH = 0, the water oxidation potential (E_O2/H2O_) and reduction potential (E_H+/H2_) are located at −5.67 eV and −4.44 eV, respectively. While, the respective E_O2/H2O_ and E_H+/H2_ shift to −5.26 eV and −4.03 eV at PH = 7, respectively. Under photoexcitation, electrons in the valence bands of MoTe_2_ layer (WTe_2_ layer) and CrI_3_ layer are excited to their respective conduction bands. The CrI_3_/MoTe_2_ HJ and CrI_3_/WTe_2_ HJ exhibit reduced bandgaps of 0.9 eV and 0.85 eV, respectively, which are significantly narrower than those of the constituent monolayers.

The results demonstrate that interfacial recombination of photogenerated electron‐hole pairs is more favorable than intralayer charge recombination in the vicinity of the interface. Furthermore, the interfacial charge transfer induces the band bending, which improves more efficient recombination between electrons in the CBM of CrI_3_ and holes in the VBM of MoTe_2_ (or WTe_2_). Concurrently, the synergistic interplay of Coulomb repulsion and band bending effectively impedes the interlayer migration of photogenerated electrons from the CBM of CrI_3_ to the VBM of MoTe_2_ (WTe_2_), as well as hindering the migration of holes from the VBM of MoTe_2_ (WTe_2_) to the VBM of CrI_3_. These results further indicate that electrons and holes remain predominantly localized at the CBM of MoTe_2_ (WTe_2_) and VBM of CrI_3_, respectively, demonstrating characteristic Z‐scheme charge transfer path. Significantly, the CBM of MoTe_2_ (WTe_2_) and VBM of CrI_3_ surpass the standard redox potentials for water reduction and oxidation, respectively, endowing the photogenerated carriers with enhanced redox capabilities. Collectively, these results confirm that the CrI_3_/MoTe_2_ HJ and CrI_3_/WTe_2_ HJ fully satisfy the essential requirements for Z‐scheme overall photocatalytic water splitting.

**FIGURE 6:**
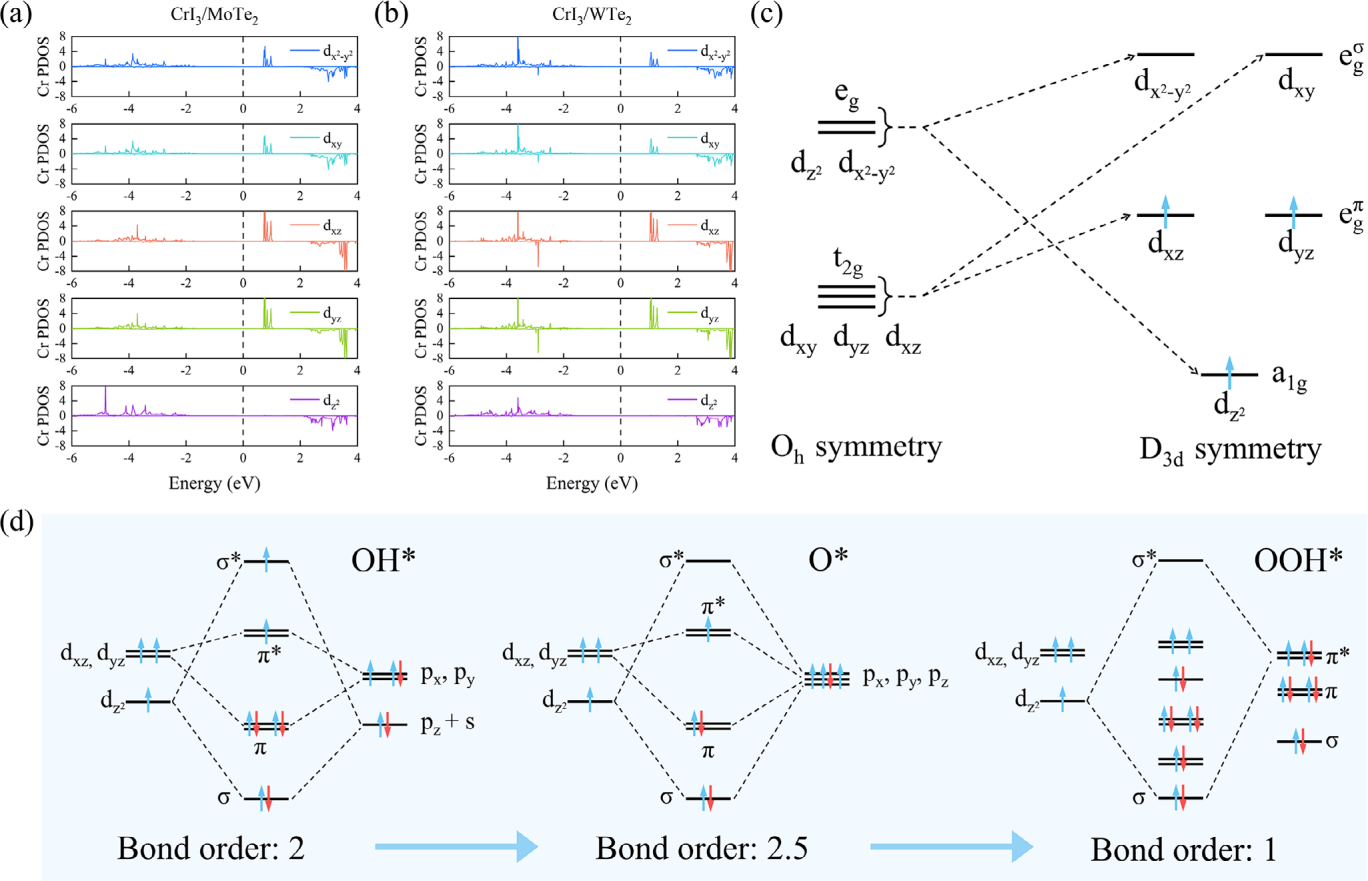
The PDOS of Cr^3+^ ions in CrI_3_/MoTe_2_ HJ (a) and CrI_3_/WTe_2_ HJ (b), the purple, blue, cyan, orange, and green curves represent the PDOS of the d_z2_, d_x2‐y2_, d_xy_, d_xz_, and d_yz_ orbitals, respectively. The dashed line indicates the Fermi level, while the dashed circle highlights the PDOS of the occupied states near the Fermi level. Within the circled region, the sharp peak of the purple curves is positioned farthest from the Fermi level, followed sequentially by blue (orange) line, with the cyan (green) appearing closest to Fermi level. The d‐orbital splitting patterns under O_h_ and D_3d_ symmetry groups (c). The crystal structure shown in the figure is CrI_3_, which exhibits D_3d_ symmetry. The orbital interactions between Cr^3+^ ions and the OER intermediates (d). The bond orders for each reaction step are explicitly indicated in the lower section of the figure.

Recent research indicates that the spin state of transition‐metals sites plays a significant yet poorly understood role in the oxygen evolution reaction (OER) [41, 42] It is thus essential to investigate the spin state of Cr^3+^ ions in CrI_3_/MoTe_2_ and CrI_3_/WTe_2_ HJs. Traditionally, the sixfold coordination of the metal cations in the dihalides and trihalides has been taken to imply octahedral symmetry (O_h_), which gives rise to the classic splitting of d‐orbitals into the e_g_ doublet (d_x2‐y2_, d_z2_) and t_2g_ triplet (d_xy_, d_yz_, d_xz_), as shown in Figure 6c. However, recent studies reveal that these halides often adopt structures with trigonal symmetries (such as D_3d_, D_3_ groups and their subgroups) [43]. In such a symmetry reduction from O_h_ to trigonal, the d‐orbital degeneracy is lifted, resulting in two doublets— e_g_^σ^ (d_xy_, d_x2‐y2_) and e_g_^π^ (d_xz_, d_yz_) doublets and one singlet a_1g_ (d_z2_). Within crystal field theory, the e_g_^σ^ and the e_g_^π^ doublets are degenerate but described by different wavefunctions, with their specific energy ordering determined by the nature of the trigonal distortion.

To further determine the d‐orbital splitting of Cr^3+^ ions in CrI_3_/MoTe_2_ and CrI_3_/WTe_2_ HJs, we calculated their PDOS in both heterojunctions. The results are presented in Figure 6a,b. In both HJs, the complete overlap of the PDOS between the d_yz_ and d_xz_ orbitals of Cr^3+^ ions strongly supports their assignment the e_g_^π^ doublet. Similarly, the perfect overlap of the PDOS for d_xy_ and d_x2‐y2_ orbitals confirms their assignment to the e_g_^σ^ doublet. Notably, although the PDOS peaks of e_g_^π^ doublet and e_g_^σ^ doublet are the same in energy, the peak intensity of the e_g_^π^ doublet is significantly higher than that of e_g_^σ^, indicating that the two doublets are not fully degenerate and the e_g_^π^ doublet has a greater tendency to capture electrons. This phenomenon can be attributed to symmetry reduction induced by the heterojunction formation and associated built‐in electric field. In contrast, the d_z2_ orbital exhibits no such overlap with any other orbitals, consistent with its classification into the a_1g_ singlet. Furthermore, its PDOS peaks reside at lower energy compared to those of e_g_^π^ doublet and e_g_^σ^ doublet, indicating a lower orbital energy level. [44] Based on above analysis, the d‐orbital splitting of Cr^3^^+^ in the heterojunctions follows the sequence: e_g_^σ^ (d_xy_, d_x2‐y2_) > e_g_^π^ (d_xz_, d_yz_) > a_1g_ (d_z2_), as illustrated in Figure 6c.

The Figure 6d illustrates the OER process within this orbital configuration [45, 46, 47]. From a thermodynamic perspective, an optimal OER catalyst should strongly adsorb of *OH while enabling rapid desorption of O_2_ to ensure efficient reaction progression and product release. To quantitatively assess adsorption strength, we analysed the bond orders of each intermediate species bound to Cr^3+^. The bond order is defined as half the difference between the number of bonding electrons and antibonding electrons (Bond Order = (Bonding Electrons—Antibonding Electrons) / 2) [45]. Owing to orbit interactions, the bond order between Cr^3+^ and *OH is calculated to be 2, whereas that for *OOH is 1. A higher bond order value indicates stronger orbital interactions between the cation and the reaction intermediate, which is thermodynamically favourable for both reactant capture and product desorption during OER. Furthermore, in the *OOH adsorbed structure, the presence of two spin‐up lone electrons in the π orbitals suggests that subsequent oxygen evolution would generate spin‐triplet oxygen. Compared to spin‐singlet oxygen, the formation of spin‐triplet oxygen generally involves a lower reaction energy barrier. Therefore, this spin‐parallel configuration is particularly advantageous for OER, as it facilitating the release of oxygen in its ground triplet state.

### 2.4 The Synergistic Effects of Spin Polarization and Single‐Atom Anchoring

The spontaneity of photocatalytic water splitting is governed by the electrochemical potential of photoexcited electrons and holes, requiring adequate driving energy from the photogenerated carriers to overcome the reaction barriers. The Gibbs free energy change (*ΔG*) between reactants and products serves as a fundamental thermodynamic descriptor for assessing the spontaneity. Consequently, the *ΔG* values for each intermediate state were determined to assess the redox reactivity using the following equation:

(5) $$\Delta G=\Delta E+\Delta E_{ZPE}-T\Delta S-qU$$

Here, Δ*E*, Δ*E_ZPE_*, and Δ*S* represent the variations in adsorption energy, zero‐point energy, and entropy between the reactants and products, respectively. *q* denotes the charge of the carrier, while *U* corresponds to the electrode potential relative to the standard H^+^/H_2_ reduction potential, which is pH‐dependent. The HER process primarily involves two elementary steps:

(6a) $$*+H^{+}+e^{-}\to *H$$

(6b) $$*H+H^{+}+e^{-}\to *+H_{2\;}$$

Where * denotes an active site for proton (H^+^) adsorption on the heterojunction surface. The oxygen evolution reaction (OER) proceeds through four consecutive steps, as follows:

(7a) $$*+H_{2}O\;\to *\mathrm{OH}+H^{+}+e^{-}$$

(7b) $$*\mathrm{OH}\;\to *O+H^{+}+e^{-}$$

(7c) $$*O+H_{2}O\;\to \;*\mathrm{OOH}+H^{+}+e^{-}$$

(7d) $$*\mathrm{OOH}\;\to *+O_{2}+H^{+}+e^{-\;}$$

Here * denotes an adsorption site on the HJs surface, while *O, *OH, and *OOH represent adsorbed intermediates. As HER primarily occurs on the MoTe_2_ (WTe_2_) layer, OER mainly proceeds on the CrI_3_ layer. Adsorption configurations for HER were constructed by depositing H^+^ onto MoTe_2_ (WTe_2_) surface of the heterojunctions. Similarly, the intermediate adsorption configurations involving *OH, *O, and *OOH were constructed by absorbing OH, O, and OOH onto the CrI_3_ surface. Spin‐polarized calculations were employed during structural optimization, enforcing collinear alignment of magnetic moments (typically aligned along the x‐direction by default), to mimic the effect of a weak magnetic field. The most stable adsorption sites for each step were determined by evaluating the relative energies of multiple potential adsorption sites.

**FIGURE 7:**
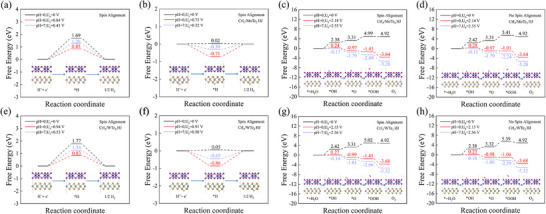
Free energy diagrams illustrate the HER and OER processes for both heterojunctions. Figure (a) and (b) present the HER pathways, respectively, for the CrI_3_/MoTe_2_ HJ and CrI_3_/Pt@MoTe_2_ HJ under spin alignment, while figure (e) and (f) show the similarly processes for CrI_3_/WTe_2_ HJ and CrI_3_/Pt@WTe_2_ HJ. Figure (c) and (d) present the OER pathways, respectively, for the CrI_3_/MoTe_2_ HJ under spin alignment and no spin alignment, while Figure (g) and (h) show the corresponding processes for CrI_3_/WTe_2_ HJ. The black line represented pure dark conditions, with red and blue lines quantifying the photogenerated electron and hole contributions at pH 0 and 7, respectively.

The potential of photogenerated electron for HER (*U_e_*) is quantitatively defined as the energy difference between the CBM of MoTe_2_(WTe_2_) and the hydrogen reduction potential. Similarly, the potentials of photogenerated hole for OER (*U_e_*) is defined as the energy difference between the VBM of CrI_3_ and the hydrogen reduction potential. Both *U*_*e*_ and *U*_*h*_, are referenced to the normal hydrogen electrode (NHE) and exhibit pH‐dependence described by the following formulas *U_e_*  =  *U_e_* (*pH*  =  0)  −  0.059  ×  *pH* and *U_h_*  =  *U_h_* (*pH*  =  0) +  0.059  ×  *pH*, respectively. As shown in Figure 7a,e, the Gibbs free energy changes (*ΔG*) for the HER on spin polarized CrI_3_/MoTe_2_ and CrI_3_/WTe_2_ HJs are 1.69 eV and 1.77 eV, respectively, in the absence of an external potential. As a result, the corresponding HER overpotentials (*η_HER_*) are 1.69 V and 1.77 V, respectively. Figure 7c,g show the free energy change for the OER process on the same heterojunctions. The black line represents the energy barrier for OER under dark conditions. The OER overpotential (*η_OER_*) is determined by the following equation: η_*OER*_  =  (Δ*G_OER_*/*e*)  −  1.23 *V*, where Δ*G_OER_* denotes the maximum free energy change among all steps. For CrI_3_/MoTe_2_ HJ, the OER proceeds with the first step as the rate‐determining step, exhibiting an *η_OER_* of 1.15 V. Similarly, for CrI_3_/WTe_2_ HJ, the rate‐ determining step is also the first reaction step, yielding a significantly higher *η_OER_* of 1.19 V. Furthermore, at pH = 7, all OER steps exhibit downhill exothermic reactions, suggesting spontaneous OER activity under neutral conditions, highlighting the strong oxidative capability of these HJs.

**FIGURE 8:**
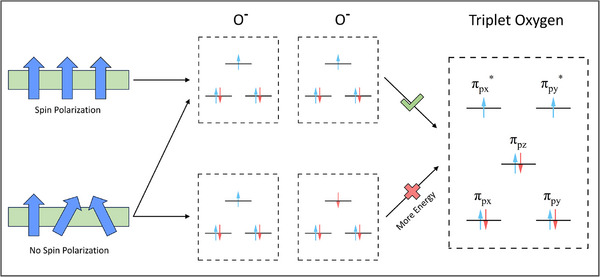
The formation pathways of triplet oxygen under both spin polarization and no polarized conditions. Notably, the formation of triplet oxygen requires higher energy under no spin polarized conditions compared to spin polarized scenarios.

To simulate the non‐polarized spin state of CrI_3_ in both HJs, we implemented non‐collinear spin‐polarized calculations, which relax spin degrees of freedom along all three Cartesian axes (x, y, and z) [26]. In this state, magnetic moments of the adsorbates change significantly, leading to altered reaction energy barriers. Figure 7d,h presents the OER free energy diagrams under the no spin polarized state. According to the projected band structure shown in Figure S4, *U_e_* and *U_h_* remains largely unchanged compared to the spin polarized condition, the energy barriers for the first two steps show minor variations, whereas the third step exhibits a pronounced increase. As illustrated in Figure 8, the non‐polarized spin state is energetically unfavorable for the formation of triplet oxygen, thereby resulting in a higher energy barrier for this step. Furthermore, the hydrogen evolution reaction occurs at the non‐magnetic MoTe_2_ and WTe_2_ layers, implying that spin has a negligible influence on the reaction.

Notably, *U_e_* values for CrI_3_/MoTe_2_ HJ and CrI_3_/WTe_2_ HJ are1.69 V and 1.77 V at pH = 7, respectively, indicating poor inertness HER activity. An efficient photocatalytic system for HER requires optimal hydrogen adsorption strength with a Gibbs free energy change (*ΔG*) close to zero. To enhance HER performance, single Pt atoms—which is a highly active HER catalyst, were anchored onto the MoTe_2_ and WTe_2_ surfaces. After evaluating various anchoring configurations (Figure S6), the most stable configurations were selected, as shown in Figure 3d,i. The structural stability of the anchored heterojunctions is further supported by AIMD simulations at 300 K, as shown in Figure S7. Pt anchoring on the non‐magnetic monolayers neither induces magnetic moments nor significantly alters the magnetic properties of the CrI_3_ layer. Figure S8 present the projected band structures, demonstrating a reduced bandgap after Pt modification. Subsequently, hydrogen adsorption on the Pt active sites was investigated and the corresponding Gibbs free energy diagram for HER are presented in Figure 7b,f. The CrI_3_/Pt@MoTe_2_ HJ demonstrates significantly improved HER activity, with the *ΔG* decreasing to 0.02 eV under acidic conditions (pH = 0) without illumination (*U_e_ =* 0 V), corresponding to an overpotential (*η_HER_*) of 0.02 V. Similarly, the CrI_3_/Pt@WTe_2_ HJ achieves an exceptionally low *η_HER_* of 0.02 V under the same conditions. These results confirm that single‐atom Pt anchoring effectively enhances HER performance without adversely affecting the heterojunction properties.

### 2.5 Optical Absorption and STH Efficiency

The optical absorption efficiency is a critical parameter for evaluating photocatalytic performance. Therefore, the design of photocatalysts with enhanced light‐harvesting capacity and optimized electronic structures is essential to achieve efficient solar‐driven hydrogen evolution. In this work, we systematically investigated the optical absorption characteristics of the CrI_3_/MoTe_2_ HJ and CrI_3_/WTe_2_ HJ through dielectric function analysis:

(8) $$\varepsilon \;\left(\omega \right)=\varepsilon_{1}\;\left(\omega \right)+i\varepsilon_{2}\left(\omega \right)$$

(9) $$\alpha \;\left(\omega \right)=\sqrt{2}\;\omega \sqrt{\sqrt{\varepsilon_{1}^{2}\left(\omega \right)+\varepsilon_{2}^{2}\left(\omega \right)}-\varepsilon_{1}\left(\omega \right)}$$

**FIGURE 9:**
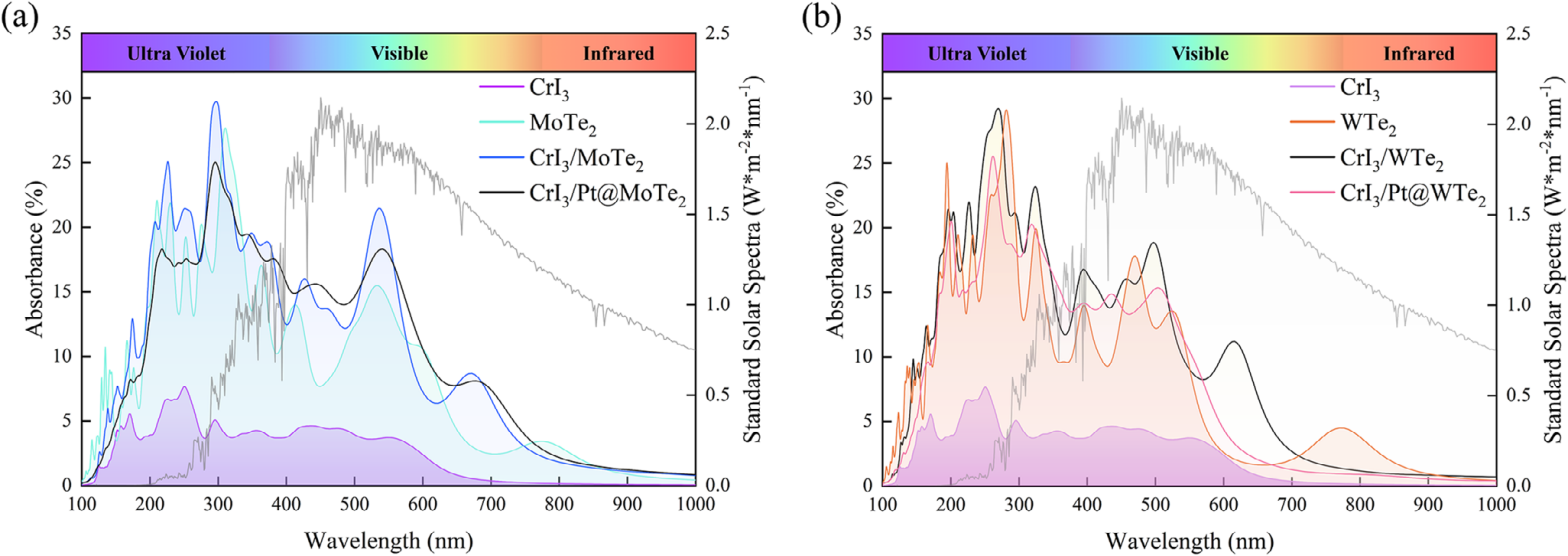
Optical absorption spectra of the related materials. The purple curve corresponds to the absorption spectrum of monolayer CrI_3_, while the cyan and orange curves represent the absorption characteristics of monolayer MoTe_2_ and WTe_2_, respectively. The gray line shows the reference solar spectrum (AM1.5G). The blue and pink curves represent the absorption profiles of CrI_3_/MoTe_2_ HJ and CrI_3_/WTe_2_ HJ. The black curve depicts the absorption profiles of CrI_3_/Pt@ MoTe_2_ HJ and CrI_3_/ Pt@ WTe_2_ HJ.

In the investigation of optical properties, the dielectric function *ε(ω)* is fundamentally associated with dipole transition probabilities between the valence band and conduction band. The parameter *ε*_*1*_ *(ω)* is mathematically derived from *ε*_*2*_ *(ω)* via the Kramers‐Kronig relations [48]. For comparative analysis, the optical absorption spectra of CrI_3_ ML, MoTe_2_ ML, WTe_2_ ML, and their corresponding HJs are presented in Figure 9a,b.

Notably, the heterojunction systems exhibit broadened absorption spectra compared to its constituent monolayers. The STH efficiency, a pivotal metric for evaluating photocatalytic water splitting performance, can be quantified using the following equation [49]:

(10) $$\eta_{\mathrm{STH}}=\frac{1}{2}\;\times \frac{\Delta G\;\int_{E}^{\infty }\frac{P\left(\hbar \omega \right)}{\hbar \omega }d\left(\hbar \omega \right)}{\int_{0}^{\infty }P\left(\hbar \omega \right)d\left(\hbar \omega \right)}$$

Here, *ΔG* represents the thermodynamic minimum potential required for water splitting, and *E* denotes the incident photon energy. *P* *(ℏω)* denotes the spectral photon flux density under AM1.5G solar illumination at photon energy ℏω. The calculated STH efficiency reach 15.19% for the CrI_3_/MoTe_2_ HJ and 14.28% for the CrI_3_/MoTe_2_ HJ, both exceeding the threshold for economically viable large‐scale hydrogen production and demonstrating strong potential for industrial application. Anchoring single‐atom Pt narrows the energy band gap, leading to additional improvements in STH conversion efficiency. Consequently, the STH efficiency rose to 16.97% for CrI_3_/MoTe_2_ HJ, and 15.61% for CrI_3_/WTe_2_ HJ, indicating a markedly enhanced light‐harvesting capability.

### 2.6 The Outlook of FQFE in Photocatalysis

According to the recently proposed theory [50, 51, 52, 53], even monolayers belonging to non‐polar point groups can exhibit FQFE. This theoretical breakthrough extends ferroelectricity beyond the conventional constraints of polar point groups and small ionic displacements, challenging the classical understanding—based on Neumann's principle—that the polarization direction must comply with the symmetry of the polar phase, systems with FQFE may host symmetry‐forbidden polarization. Specifically, among the selected TMHs, MoS_2_‐type AB_2_ (A = Sc, Y; B = Cl, Br, I) monolayer with the P‐6m2 (No. 187) symmetry hosts a fractional in‐plane ferroelectric polarization. As illustrated in Figure S1, during the ferroelectric transition from L1 to L2, the A atom relocates from (1/3, 2/3, z) to (2/3, 1/3, z), whereas the B atoms remain fixed. Consequently, the atomic displacements are Δd_A_ = (1/3, −1/3, 0) and Δd_B_ = 0. The resulting polarization change during this transition is given by:

(11) $$\Delta P=2\times \frac{1}{3}\left(a-b\right)\;\frac{e}{\Omega }=\frac{2}{3}\;Q$$

where 2 represents the valence charge of the A atom, Q = $\left(a-b\right)\frac{e}{\Omega }$ is the polarization quantum, ***a***, ***b*** denote the lattice vectors, and Ω is the cell volume. The specific values of the ferroelectric polarization for each material are summarized in Table 1. Hence, MoS_2_‐type AB_2_ emerges as a promising fractional ferroelectric material. In addition to its ferromagnetism, MoS_2_‐type AB_2_ also exhibits fractional quantum ferroelectricity with a large polarization magnitude. Such materials provide a new platform for the discovery and design of next‐generation multiferroics for spintronic applications [54].

**TABLE 1:** Structural parameters and spontaneous ferroelectric polarization of AB_2_ (A = Sc, Y; B = Cl, Br, I) monolayer. The values of effective thicknesses come from the van der Waals Bilayer Database (BiDB) [55].

Formula	Lattice vector (along the [1 ‐1 0] direction) (Å)	Effective thickness (Å)	Ferroelectric polarization (µC/m^2^)
YCl_2_	6.46	6.5	44.05
YBr_2_	6.7	6.96	39.7
YI_2_	7.11	7.58	34.32
ScCl_2_	6.09	6.39	47.48
ScBr_2_	6.4	6.8	42.53
ScI_2_	6.89	7.4	36.26

As for classical ferroelectrics, the out‐of‐plane polarization can generate a built‐in electric field, which is recognized to facilitate the separation of photogenerated carriers and suppress their recombination [56, 57]. Meanwhile, ferroelectric polarization can modulate the surface charge distribution and the associated interfacial charge transfer processes, thereby directly regulating the HER activity [58]. Additionally, such polarization‐enabled electrostatic regulation may further help optimize the adsorption free energy of hydrogen intermediates and lower kinetic barriers, offering an effective route to enhance catalytic performance [59].

In contrast, the in‐plane polarization of FQFE introduces unique effects that are not yet fully understood in the context of photocatalytic processes. Establishing a comprehensive relationship between this polarization and photocatalytic efficiency, such as enhanced charge separation efficiency, is challenging at present. Here, we emphasize that FQFE offers new prospects for multiferroic photocatalysis, and clarifying its mechanistic role in photocatalysis warrants dedicated and systematic future studies.

## 3 Conclusions

In summary, our investigation commenced with high‐throughput screening, which narrowed down the candidates to 67 magnetic heterojunctions from an initial pool of candidates. Detailed first‐principles calculations on selected CrI_3_/MoTe_2_ and CrI_3_/WTe_2_ heterostructures confirm a direct Z‐scheme charge transfer pathway, validated by charge density difference and electrostatic potential analyses. The band structures and free energy diagrams further verify their thermodynamic feasibility for overall water splitting. Crucially, the density of states analysis reveals a distinct Cr^3+^ 3d orbital configuration within the CrI_3_ layer: e_g_^σ^ (d_xy_, d_x2‐y2_) > e_g_^π^ (d_xz_, d_yz_) > a_1g_ (d_z2_). This electronic structure, consistent with bond order theory, favors OH^−^ adsorption and O_2_ dissociation. Furthermore, the parallel spin alignment of unpaired electrons during OOH* adsorption further promotes the generation of triplet oxygen. Non‐collinear spin calculations demonstrate that non‐parallel spin arrangements in the absence of an external magnetic field significantly increase the energy barrier of the OOH* step, thereby impeding triplet oxygen evolution. To address the non‐ideal H^+^ adsorption on the MoTe_2_ and WTe_2_ layers, Pt single‐atom anchoring was implemented, which substantially improves HER activity while preserving the intrinsic magnetic properties. Optical absorption calculations demonstrate strong visible‐light harvesting in both heterojunctions. The resulting estimated solar‐to‐hydrogen conversion efficiencies surpass key industrial benchmarks, highlighting the promise of these magnetic heterojunctions as efficient photocatalysts for overall water splitting. Moreover, the discovery of fractional ferroelectric semiconductors via high‐throughput screening not only opens a novel avenue for designing advanced multiferroic photocatalysts but also warrants in‐depth future studies to fully exploit their potential for achieving unprecedented photocatalytic efficiency.

## 4 Computational Methods

### 4.1 High‐Throughput Screening

High‐throughput screening is carried out by retrieving an extensive dataset of 2D materials from the C2DB database, which supplies critical material parameters such as elemental composition, crystal structure (including point group type and lattice constants), electronic properties, magnetic characteristics, and thermodynamic stability [28, 29]. The screening process for 2D materials with targeted properties can be efficiently automated using Python code.

### 4.2 DFT Calculation

In this work, first principles calculations based on density functional theory (DFT) were conducted using the Vienna Ab initio Simulation Package (VASP), with the projector augmented‐wave (PAW) method employed to describe electron‐ion interactions [60, 61, 62]. The exchange‐correlation interaction was described by the generalized gradient approximation (GGA), specifically using the Perdew–Burke–Ernzerhof (PBE) functional [63, 64]. To better incorporate van der Waals (vdW) interactions, the DFT‐D3 empirical dispersion correction scheme was employed [65]. Considering the correlation effects of the 3d electrons in Cr atoms, the DFT+U method was adopted for structural optimization, with an effective U_eff_ = 2.5 eV for Cr [66, 67, 68]. Owing to the bandgap underestimation inherent to the PBE functional, electronic and optical properties were computed with the Heyd–Scuseria–Ernzerhof (HSE06) hybrid functional [69]. The plane‐wave cutoff energy was set to 500 eV, and the convergence criteria for the total energy and Hellmann‐Feynman forces on each individual atoms were strictly set to 10^−5^ eV and 10^−2^ eV/Å, respectively. For static calculations, a k‐point grid of 9 × 9 × 1 mesh was assigned to the Brillouin zone sampling of CrI_3,_ MoTe_2_ and WTe_2_ MLs, while a 5 × 5 × 1 k‐point mesh was utilized for the CrI_3_/MoTe_2_ and CrI_3_/WTe_2_ HJs. Additionally, a vacuum layer of 20 Å was introduced to mitigate spurious interactions arising from periodic boundary conditions. The valence electron configurations adopted in this work were as follows: Cr‐3d^5^4s^1^, I‐5s^2^5p^5^, Mo‐4d^5^5s^1^, W‐5d^4^5s^2^ and Te‐5s^2^5p^4^. Post‐processing and analysis of the computational results were conducted by using VASPKIT [70].

## Conflicts of Interest

The authors declare no conflicts of interest.

## Supporting information




**Supporting File**: advs74006‐sup‐0001‐SuppMat.docx.

## Data Availability

The data that support the findings of this study are available in the supplementary material of this article.
