# Supplementary material for: Synergistic Spin‐Polarization and Single‐Atom Engineering in Magnetic Heterojunctions for Efficient Solar Water Splitting
Source: Adv Sci (Weinh). 2026 Jan 21:e24114. Online ahead of print. doi: 10.1002/advs.202524114 (PMC13325478; doi:10.1002/advs.202524114)
Supplement: Supplementary file 1 — Supporting File: advs74006‐sup‐0001‐SuppMat.docx. [file ADVS-9999-e24114-s001.docx]

Supporting Information

Synergistic Spin-Polarization and Single-Atom Engineering in Magnetic Heterojunctions for Efficient Solar Water Splitting

Hongyang Ren^#^, Zhenzhou Guo^#^, Huirong Wu, Peilin Huang, Jie Yang, Jie Zhang, Guangqian Ding*, Hongkuan Yuan*, Biao Wang*

High-throughput screening:

Table S1. All two-dimensional transition metal halides

| Formula | Point group | Magnetic moment | Work  fuction | vbm | cbm | Band  gap | a_length | b_length | space_group | uid | Dynamically stable | Energy above hull |
| --- | --- | --- | --- | --- | --- | --- | --- | --- | --- | --- | --- | --- |
| Au2I2 | 4/mmm | - | 4.607 | -2.063 | -0.471 | 1.592 | 4.227 | 4.227 | P4/nmm | 2AuI-3 | No | 0.163 |
| Au2I2 | -3m | - | 4.588 | -1.923 | -0.295 | 1.628 | 4.443 | 4.444 | P-3m1 | 2AuI-2 | No | 0.148 |
| Cd2F2 | 4/mmm | - | 4.956 | - | - | - | 4.279 | 4.279 | P4/nmm | 2CdF-2 | No | 0.436 |
| Cd2F2 | -3m | - | 5.711 | - | - | 0 | 3.442 | 3.443 | P-3m1 | 2CdF-1 | No | 0.18 |
| Co2I2 | -3m | 4 | 3.854 | - | - | - | 3.888 | 3.889 | P-3m1 | 2CoI-2 | Yes | 0.283 |
| Co2I2 | 4/mmm | 4 | 3.569 | - | - | - | 3.926 | 3.926 | P4/nmm | 2CoI-3 | No | 0.323 |
| Ag2Br4 | 2/m | - | 6.557 | - | - | - | 4.191 | 6.69 | C2/m | 2AgBr2-2 | No | 0.038 |
| AuBr2 | -3m | 0.856 | 6.042 | - | - | - | 4.063 | 4.063 | P-3m1 | 1AuBr2-1 | No | 0.152 |
| Au2Br4 | 2/m | - | 6.049 | - | - | - | 4.364 | 6.791 | C2/m | 2AuBr2-1 | No | 0.103 |
| AuBr2 | -42m | - | 6.269 | - | - | - | 3.996 | 3.996 | P-4m2 | 1AuBr2-2 | No | 0.171 |
| AuBr2 | -6m2 | 0.911 | 6.177 | - | - | - | 3.843 | 3.843 | P-6m2 | 1AuBr2-3 | No | 0.277 |
| CdCl2 | -42m | - | 6.118 | -6.706 | -1.722 | 4.984 | 4.085 | 4.085 | P-4m2 | 1CdCl2-2 | Yes | 0.043 |
| CdCl2 | -3m | - | 6.07 | -6.042 | -0.866 | 5.176 | 3.9 | 3.9 | P-3m1 | 1CdCl2-1 | Yes | 0 |
| CdCl2 | -6m2 | - | 6.461 | -5.785 | -1.441 | 4.344 | 3.735 | 3.735 | P-6m2 | 1CdCl2-3 | No | 0.13 |
| CoBr2 | -42m | 3 | 5.682 | -4.715 | -1.888 | 2.827 | 3.88 | 3.88 | P-4m2 | 1CoBr2-1 | Yes | 0.009 |
| CoBr2 | -6m2 | 3 | 5.476 | - | - | - | 3.576 | 3.576 | P-6m2 | 1CoBr2-3 | Unknown | 0.177 |
| CoBr2 | -3m | 3 | 5.782 | -4.126 | -0.598 | 3.528 | 3.732 | 3.732 | P-3m1 | 1CoBr2-2 | Yes | 0.023 |
| CuI2 | -42m | - | 5.689 | - | - | - | 3.943 | 3.943 | P-4m2 | 1CuI2-1 | Yes | 0 |
| Cu2I4 | 2/m | - | 5.586 | - | - | - | 4.106 | 6.638 | C2/m | 2CuI2-2 | No | 0.074 |
| CuI2 | -6m2 | -0.56 | 5.501 | - | - | - | 3.856 | 3.856 | P-6m2 | 1CuI2-3 | No | 0.223 |
| CuI2 | -3m | - | 5.569 | - | - | - | 3.941 | 3.941 | P-3m1 | 1CuI2-2 | No | 0.077 |
| HfI2 | -42m | - | 3.946 | - | - | - | 3.713 | 3.713 | P-4m2 | 1HfI2-3 | No | 0.585 |
| Hf2I4 | 2/m | - | 3.7 | 0.115 | 1.13 | 1.014 | 3.716 | 6.829 | P2_1/m | 2HfI2-1 | Yes | 0.063 |
| HfI2 | -6m2 | - | 2.789 | 1.147 | 2.168 | 1.022 | 3.773 | 3.773 | P-6m2 | 1HfI2-1 | Yes | 0.114 |
| HfI2 | -3m | - | 3.303 | - | - | - | 3.723 | 3.723 | P-3m1 | 1HfI2-2 | No | 0.242 |
| IrBr2 | -42m | - | 4.434 | - | - | - | 3.668 | 3.668 | P-4m2 | 1IrBr2-2 | No | 0.671 |
| IrBr2 | -6m2 | - | 4.48 | - | - | - | 3.629 | 3.629 | P-6m2 | 1IrBr2-3 | No | 0.95 |
| Ir2Br4 | 2/m | - | 4.27 | - | - | - | 3.703 | 7.37 | P2_1/m | 2IrBr2-1 | No | 0.481 |
| IrBr2 | -3m | 0.979 | 3.981 | - | - | - | 3.87 | 3.87 | P-3m1 | 1IrBr2-1 | No | 0.51 |
| MnBr2 | -42m | 5 | 5.142 | -5.003 | -0.58 | 4.424 | 4.067 | 4.067 | P-4m2 | 1MnBr2-2 | Yes | 0.033 |
| MnBr2 | -3m | 5 | 5.128 | -4.131 | 0.157 | 4.289 | 3.89 | 3.89 | P-3m1 | 1MnBr2-1 | Yes | 0 |
| MnBr2 | -6m2 | 5 | 5.527 | - | - | - | 3.748 | 3.748 | P-6m2 | 1MnBr2-3 | No | 0.129 |
| MoBr2 | -6m2 | 1.246 | 3.802 | - | - | - | 3.368 | 3.368 | P-6m2 | 1MoBr2-3 | No | 0.804 |
| Mo2Br4 | 2/m | 1.158 | 3.401 | - | - | - | 3.368 | 6.307 | P2_1/m | 2MoBr2-1 | No | 0.55 |
| MoBr2 | -3m | - | 3.661 | - | - | - | 3.379 | 3.379 | P-3m1 | 1MoBr2-1 | No | 0.59 |
| MoBr2 | -42m | - | 4.217 | - | - | - | 3.277 | 3.277 | P-4m2 | 1MoBr2-2 | No | 0.787 |
| Nb2I4 | 2/m | - | 2.98 | - | - | - | 3.808 | 6.569 | P2_1/m | 2NbI2-1 | Yes | 0.123 |
| NiI2 | -3m | 1.998 | 4.881 | -2.386 | -0.482 | 1.904 | 3.97 | 3.97 | P-3m1 | 1NiI2-1 | Yes | 0 |
| NiI2 | -42m | 1.773 | 5.37 | - | - | - | 4.158 | 4.158 | P-4m2 | 1NiI2-2 | Yes | 0.099 |
| NiI2 | -6m2 | 1.998 | 4.999 | - | - | - | 3.851 | 3.851 | P-6m2 | 1NiI2-3 | No | 0.273 |
| PtCl2 | -6m2 | 0.672 | 5.699 | - | - | - | 3.556 | 3.556 | P-6m2 | 1PtCl2-3 | Unknown | 0.682 |
| PtCl2 | -3m | 1.996 | 5.26 | -3.362 | -1.498 | 1.864 | 3.789 | 3.789 | P-3m1 | 1PtCl2-1 | Yes | 0.358 |
| PtCl2 | -42m | - | 5.651 | - | - | - | 3.682 | 3.682 | P-4m2 | 1PtCl2-2 | No | 0.491 |
| ReBr2 | -3m | 1 | 3.667 | - | - | - | 3.752 | 3.752 | P-3m1 | 1ReBr2-1 | No | 0.6 |
| ReBr2 | -6m2 | 1.429 | 3.907 | - | - | - | 3.245 | 3.245 | P-6m2 | 1ReBr2-3 | No | 0.889 |
| Re2Br4 | 2/m | - | 3.376 | - | - | - | 3.885 | 5.605 | P2_1/m | 2ReBr2-1 | No | 0.562 |
| ReBr2 | -42m | - | 3.826 | - | - | - | 3.217 | 3.217 | P-4m2 | 1ReBr2-2 | No | 0.617 |
| RhI2 | -6m2 | - | 4.546 | - | - | - | 3.844 | 3.844 | P-6m2 | 1RhI2-3 | No | 0.508 |
| RhI2 | -3m | 0.952 | 4.274 | - | - | - | 4.039 | 4.039 | P-3m1 | 1RhI2-1 | Yes | 0.169 |
| RhI2 | -42m | - | 4.804 | - | - | - | 3.953 | 3.953 | P-4m2 | 1RhI2-2 | No | 0.394 |
| RuI2 | -42m | - | 4.465 | - | - | - | 3.826 | 3.826 | P-4m2 | 1RuI2-2 | No | 0.487 |
| RuI2 | -6m2 | 2.504 | 4.292 | - | - | - | 3.898 | 3.898 | P-6m2 | 1RuI2-3 | No | 0.592 |
| RuI2 | -3m | - | 4.034 | -1.635 | 0.668 | 2.303 | 4.026 | 4.026 | P-3m1 | 1RuI2-1 | Yes | 0.102 |
| TaCl2 | -3m | - | 3.788 | - | - | - | 3.15 | 3.15 | P-3m1 | 1TaCl2-1 | Yes | 0.261 |
| TaCl2 | -42m | - | 4.521 | - | - | - | 3.087 | 3.087 | P-4m2 | 1TaCl2-3 | No | 0.5 |
| TaCl2 | -6m2 | - | 3.351 | - | - | - | 3.151 | 3.151 | P-6m2 | 1TaCl2-2 | No | 0.345 |
| W2Cl4 | 2/m | - | 3.329 | - | - | - | 3.11 | 5.408 | P2_1/m | 2WCl2-1 | Yes | 0.52 |
| ZrF2 | -42m | - | 5.888 | - | - | - | 2.919 | 2.919 | P-4m2 | 1ZrF2-3 | No | 0.588 |
| Au2Cl6 | -3m | 3.92 | 6.589 | -4.815 | -3.539 | 1.276 | 6.505 | 6.505 | P-31m | 2AuCl3-1 | Yes | 0.169 |
| Au2Cl6 | 6/mmm | 0.864 | 7.022 | - | - | - | 6.838 | 6.838 | P6/mmm | 2AuCl3-2 | No | 0.313 |
| W2Cl6 | -3m | - | 4.65 | -1.276 | -0.393 | 0.883 | 5.557 | 5.557 | P-31m | 2WCl3-2 | Yes | 0.108 |
| W2Cl6 | -6m2 | - | 4.186 | -0.975 | 0.19 | 1.165 | 5.525 | 5.525 | P-62m | 2WCl3-3 | Yes | 0.164 |
| Cu2Br6 | -3m | 3.899 | 6.416 | -4.448 | -3.805 | 0.643 | 6.481 | 6.481 | P-31m | 2CuBr3-1 | Yes | 0.091 |
| Cu2Br6 | -6m2 | 0.972 | 6.556 | - | - | - | 6.81 | 6.81 | P-62m | 2CuBr3-2 | No | 0.183 |
| Mn2Cl6 | -3m | 4.009 | 5.317 | -2.245 | -1.366 | 0.879 | 5.901 | 5.901 | P-31m | 2MnCl3-2 | Yes | 0.073 |
| Mn2Cl6 | 6/mmm | 8 | 6.729 | - | - | - | 6.512 | 6.512 | P6/mmm | 2MnCl3-3 | No | 0.075 |
| Nb2Br6 | -3m | 1.993 | 4.638 | - | - | - | 6.204 | 6.204 | P-31m | 2NbBr3-2 | No | 0.126 |
| Nb2Br6 | -6m2 | - | 4.49 | - | - | - | 6.206 | 6.206 | P-62m | 2NbBr3-3 | No | 0.157 |
| Ta2I6 | -6m2 | - | 4.003 | - | - | - | 6.732 | 6.732 | P-62m | 2TaI3-2 | Yes | 0.214 |
| Ta2I6 | -3m | 1.893 | 4.125 | - | - | - | 6.743 | 6.743 | P-31m | 2TaI3-1 | No | 0.188 |
| Zr2Cl6 | -3m | 1.994 | 4.527 | - | - | - | 6.248 | 6.248 | P-31m | 2ZrCl3-1 | No | 0.055 |
| Zr2Cl6 | -6m2 | 1.98 | 4.356 | - | - | - | 6.264 | 6.264 | P-62m | 2ZrCl3-2 | Yes | 0.083 |
| Mo2Cl6 | 2/m | 1.999 | 5.156 | -4.446 | -4.274 | 0.172 | 5.76 | 5.76 | C2/m | 2MoCl3-1 | Yes | 0 |
| Mo2Cl6 | -6m2 | - | 4.811 | -1.575 | -0.76 | 0.815 | 5.59 | 5.59 | P-62m | 2MoCl3-3 | Yes | 0.139 |
| Fe2I2 | 4/mmm | 6 | 3.503 | - | - | - | 3.804 | 3.804 | P4/nmm | 2FeI-1 | Yes | 0.211 |
| Fe2I2 | -3m | 6 | 3.556 | - | - | - | 3.972 | 3.973 | P-3m1 | 2FeI-2 | No | 0.227 |
| AgBr2 | -42m | - | 6.828 | - | - | - | 3.894 | 3.894 | P-4m2 | 1AgBr2-2 | No | 0.11 |
| AgBr2 | -3m | 0.863 | 6.579 | - | - | - | 3.991 | 3.991 | P-3m1 | 1AgBr2-1 | No | 0.052 |
| AgBr2 | -6m2 | 0.974 | 6.581 | - | - | - | 3.824 | 3.824 | P-6m2 | 1AgBr2-3 | No | 0.147 |
| AuCl2 | -6m2 | 0.921 | 6.761 | - | - | - | 3.687 | 3.687 | P-6m2 | 1AuCl2-3 | No | 0.351 |
| AuCl2 | -42m | - | 6.7 | - | - | - | 3.87 | 3.87 | P-4m2 | 1AuCl2-2 | No | 0.255 |
| AuCl2 | -3m | 0.936 | 6.455 | - | - | - | 3.964 | 3.964 | P-3m1 | 1AuCl2-1 | No | 0.213 |
| CdF2 | -42m | - | 6.715 | -8.721 | -2.838 | 5.883 | 3.773 | 3.773 | P-4m2 | 1CdF2-2 | No | 0.208 |
| CoCl2 | -42m | 3 | 5.966 | -4.846 | -2.179 | 2.667 | 3.682 | 3.682 | P-4m2 | 1CoCl2-1 | Yes | 0.01 |
| CoCl2 | -3m | 3 | 6.067 | -4.655 | -0.856 | 3.799 | 3.537 | 3.537 | P-3m1 | 1CoCl2-2 | Yes | 0.021 |
| CoCl2 | -6m2 | 3 | 5.858 | - | - | - | 3.376 | 3.376 | P-6m2 | 1CoCl2-3 | Unknown | 0.187 |
| CrBr2 | -6m2 | 4 | 5.114 | - | - | - | 3.693 | 3.693 | P-6m2 | 1CrBr2-3 | No | 0.262 |
| CrBr2 | -3m | 4 | 4.675 | - | - | - | 3.841 | 3.841 | P-3m1 | 1CrBr2-1 | No | 0.099 |
| CrBr2 | -42m | 4 | 5.182 | - | - | - | 3.918 | 3.918 | P-4m2 | 1CrBr2-2 | No | 0.203 |
| HgBr2 | -3m | - | 5.916 | -4.788 | -1.86 | 2.928 | 4.143 | 4.143 | P-3m1 | 1HgBr2-3 | No | 0.038 |
| HgBr2 | -42m | - | 5.92 | -5.454 | -2.392 | 3.062 | 4.255 | 4.255 | P-4m2 | 1HgBr2-2 | Yes | 0.019 |
| HgBr2 | -6m2 | - | 6.139 | -4.572 | -2.113 | 2.459 | 3.976 | 3.976 | P-6m2 | 1HgBr2-4 | No | 0.138 |
| MnCl2 | -6m2 | 5 | 5.899 | - | - | - | 3.56 | 3.56 | P-6m2 | 1MnCl2-3 | No | 0.136 |
| MnCl2 | -3m | 5 | 5.4 | -4.752 | 0.084 | 4.836 | 3.72 | 3.72 | P-3m1 | 1MnCl2-1 | Yes | 0 |
| MnCl2 | -42m | 5 | 5.359 | -5.57 | -0.551 | 5.019 | 3.91 | 3.91 | P-4m2 | 1MnCl2-2 | Yes | 0.035 |
| Mo2Cl4 | 2/m | - | 3.803 | - | - | - | 3.173 | 5.516 | P2_1/m | 2MoCl2-1 | Yes | 0.476 |
| NbI2 | -6m2 | - | 3.211 | - | - | - | 3.631 | 3.631 | P-6m2 | 1NbI2-2 | No | 0.376 |
| NbI2 | -42m | - | 4.003 | - | - | - | 3.649 | 3.649 | P-4m2 | 1NbI2-3 | No | 0.622 |
| NbI2 | -3m | - | 3.539 | - | - | - | 3.708 | 3.708 | P-3m1 | 1NbI2-1 | No | 0.316 |
| PdBr2 | -6m2 | - | 5.783 | - | - | - | 3.682 | 3.682 | P-6m2 | 1PdBr2-3 | No | 0.406 |
| PdBr2 | -3m | 1.981 | 5.577 | -3.642 | -2.117 | 1.525 | 3.904 | 3.904 | P-3m1 | 1PdBr2-1 | Yes | 0.148 |
| PdBr2 | -42m | - | 5.887 | - | - | - | 3.824 | 3.824 | P-4m2 | 1PdBr2-2 | No | 0.324 |
| Sc2Br4 | 2/m | - | 3.436 | - | - | - | 3.76 | 6.513 | P2_1/m | 2ScBr2-1 | No | 0.016 |
| TiBr2 | -6m2 | - | 3.496 | 0.105 | 1.27 | 1.166 | 3.472 | 3.472 | P-6m2 | 1TiBr2-1 | Yes | 0.033 |
| TiBr2 | -3m | 1.999 | 3.709 | - | - | - | 3.737 | 3.737 | P-3m1 | 1TiBr2-2 | No | 0.095 |
| TiBr2 | -42m | 2 | 4.177 | - | - | - | 3.673 | 3.673 | P-4m2 | 1TiBr2-3 | Unknown | 0.319 |
| VI2 | -6m2 | 3 | 4.055 | - | - | - | 3.957 | 3.957 | P-6m2 | 1VI2-3 | No | 0.277 |
| VI2 | -42m | 3 | 4.372 | - | - | - | 4.184 | 4.184 | P-4m2 | 1VI2-2 | No | 0.268 |
| VI2 | -3m | 3 | 3.947 | -2.31 | 1.085 | 3.395 | 4.126 | 4.126 | P-3m1 | 1VI2-1 | Yes | 0 |
| WCl2 | -6m2 | - | 3.624 | - | - | - | 3.05 | 3.05 | P-6m2 | 1WCl2-3 | No | 0.736 |
| WCl2 | -42m | - | 4.287 | - | - | - | 3.03 | 3.03 | P-4m2 | 1WCl2-2 | No | 0.6 |
| WCl2 | -3m | - | 3.28 | - | - | - | 3.109 | 3.109 | P-3m1 | 1WCl2-1 | No | 0.521 |
| Y2Br4 | 2/m | - | 3.428 | - | - | - | 3.894 | 6.784 | P2_1/m | 2YBr2-1 | No | 0.078 |
| Co2Br6 | 6/mmm | 5.852 | 6.113 | - | - | - | 6.747 | 6.747 | P6/mmm | 2CoBr3-2 | No | 0.195 |
| Co2Br6 | -3m | - | 5.528 | -4.512 | -1.414 | 3.098 | 6.205 | 6.205 | P-31m | 2CoBr3-1 | Yes | 0 |
| Cu2Cl6 | -3m | 3.985 | 7.168 | -5.65 | -4.241 | 1.409 | 6.107 | 6.107 | P-31m | 2CuCl3-1 | Yes | 0.072 |
| Cu2Cl6 | 6/mmm | 1.611 | 7.296 | - | - | - | 6.415 | 6.415 | P6/mmm | 2CuCl3-2 | No | 0.208 |
| Nb2Cl6 | -3m | 1.988 | 4.99 | - | - | - | 5.826 | 5.826 | P-31m | 2NbCl3-1 | No | 0.117 |
| Nb2Cl6 | -6m2 | - | 4.807 | - | - | - | 5.841 | 5.841 | P-62m | 2NbCl3-2 | No | 0.152 |
| Ru2Br6 | -3m | 1.997 | 5.477 | - | - | - | 6.51 | 6.51 | P-31m | 2RuBr3-1 | Yes | 0.017 |
| Ru2Br6 | 6/mmm | 3.292 | 5.121 | - | - | - | 6.938 | 6.938 | P6/mmm | 2RuBr3-2 | No | 0.297 |
| Sc2Br6 | -3m | - | 5.659 | -5.201 | -0.379 | 4.822 | 6.822 | 6.822 | P-31m | 2ScBr3-1 | Yes | 0 |
| Sc2Br6 | -6m2 | - | 5.69 | -5.333 | -0.789 | 4.544 | 7.121 | 7.121 | P-62m | 2ScBr3-3 | No | 0.082 |
| Zr2I6 | -3m | 1.996 | 4.204 | - | - | - | 7.114 | 7.114 | P-31m | 2ZrI3-1 | No | 0.089 |
| Zr2I6 | -6m2 | 1.98 | 4.042 | - | - | - | 7.1 | 7.1 | P-62m | 2ZrI3-2 | Yes | 0.126 |
| Cr2F4 | 2/m | 8 | 4.389 | - | - | 0 | 3.702 | 5.503 | P2_1/c | 2CrF2-1 | Yes | 0.117 |
| Cd2I2 | 4/mmm | - | 5.288 | - | - | - | 4.41 | 4.41 | P4/nmm | 2CdI-3 | No | 0.461 |
| Cd2I2 | -3m | - | 5.255 | - | - | - | 4.167 | 4.168 | P-3m1 | 2CdI-2 | No | 0.245 |
| CdI2 | -6m2 | - | 5.005 | -2.848 | -0.553 | 2.295 | 4.2 | 4.2 | P-6m2 | 1CdI2-3 | No | 0.159 |
| CdI2 | -3m | - | 4.96 | -3.333 | -0.34 | 2.992 | 4.324 | 4.324 | P-3m1 | 1CdI2-2 | Yes | 0.03 |
| CdI2 | -42m | - | 4.891 | -4.156 | -0.866 | 3.29 | 4.469 | 4.469 | P-4m2 | 1CdI2-1 | Yes | 0.003 |
| IrCl2 | -3m | 0.998 | 4.201 | - | - | - | 3.715 | 3.715 | P-3m1 | 1IrCl2-1 | No | 0.588 |
| IrCl2 | -6m2 | - | 4.849 | - | - | - | 3.443 | 3.443 | P-6m2 | 1IrCl2-3 | No | 1.042 |
| IrCl2 | -42m | - | 4.694 | - | - | - | 3.472 | 3.472 | P-4m2 | 1IrCl2-2 | No | 0.694 |
| MoCl2 | -6m2 | 0.373 | 3.995 | - | - | - | 3.133 | 3.133 | P-6m2 | 1MoCl2-3 | No | 0.721 |
| MoCl2 | -42m | - | 4.584 | - | - | - | 3.084 | 3.084 | P-4m2 | 1MoCl2-2 | No | 0.673 |
| MoCl2 | -3m | - | 3.876 | - | - | - | 3.176 | 3.176 | P-3m1 | 1MoCl2-1 | No | 0.477 |
| PtF2 | -42m | 1.998 | 7.676 | - | - | - | 2.824 | 2.824 | P-4m2 | 1PtF2-2 | No | 0.614 |
| Re2Cl4 | 2/m | - | 3.575 | - | - | - | 3.706 | 5.265 | C2/m | 2ReCl2-1 | No | 0.821 |
| ScBr2 | -3m | - | 3.414 | - | - | - | 3.765 | 3.765 | P-3m1 | 1ScBr2-2 | No | 0.017 |
| ScBr2 | -42m | 1 | 3.869 | - | - | - | 3.846 | 3.846 | P-4m2 | 1ScBr2-3 | Unknown | 0.297 |
| ScBr2 | -6m2 | 0.985 | 3.846 | -0.367 | 0.39 | 0.757 | 3.694 | 3.694 | P-6m2 | 1ScBr2-1 | Yes | 0 |
| YBr2 | -3m | - | 3.372 | - | - | - | 3.891 | 3.891 | P-3m1 | 1YBr2-2 | No | 0.079 |
| YBr2 | -6m2 | 0.997 | 3.762 | -0.554 | 0.347 | 0.901 | 3.867 | 3.867 | P-6m2 | 1YBr2-1 | Yes | 0.016 |
| YBr2 | -42m | 0.843 | 4.121 | - | - | - | 3.859 | 3.859 | P-4m2 | 1YBr2-3 | No | 0.383 |
| ZnBr2 | -42m | - | 5.258 | -5.475 | -0.969 | 4.507 | 3.925 | 3.925 | P-4m2 | 1ZnBr2-1 | Yes | 0 |
| ZnBr2 | -6m2 | - | 5.404 | -4.235 | -0.787 | 3.449 | 3.691 | 3.691 | P-6m2 | 1ZnBr2-3 | No | 0.249 |
| ZnBr2 | -3m | - | 5.263 | -4.842 | -0.28 | 4.563 | 3.798 | 3.798 | P-3m1 | 1ZnBr2-2 | Yes | 0.08 |
| Au2I6 | -3m | 2.735 | 5.518 | - | - | - | 7.305 | 7.305 | P-31m | 2AuI3-2 | No | 0.14 |
| Au2I6 | 6/mmm | - | 5.599 | - | - | - | 7.71 | 7.71 | P6/mmm | 2AuI3-3 | No | 0.206 |
| Mn2I6 | -3m | 8 | 5.474 | - | - | - | 7.099 | 7.099 | P-31m | 2MnI3-1 | No | 0 |
| Re2Br6 | -3m | 4 | 4.703 | - | - | - | 6.411 | 6.411 | P-31m | 2ReBr3-1 | Yes | 0 |
| Re2Br6 | -6m2 | 1.911 | 4.165 | - | - | - | 6.072 | 6.072 | P-62m | 2ReBr3-2 | No | 0.209 |
| W2I6 | -6m2 | - | 3.923 | -0.107 | 0.104 | 0.211 | 6.508 | 6.508 | P-62m | 2WI3-3 | Yes | 0.19 |
| W2I6 | -3m | 6 | 4.368 | -2.088 | -0.058 | 2.03 | 7.201 | 7.201 | P-31m | 2WI3-2 | Yes | 0.069 |
| OsBr2 | -3m | - | 3.668 | -1.666 | 0.758 | 2.424 | 3.828 | 3.828 | P-3m1 | 1OsBr2-1 | Yes | 0.402 |
| OsBr2 | -42m | - | 3.842 | - | - | - | 3.374 | 3.374 | P-4m2 | 1OsBr2-2 | No | 0.677 |
| OsBr2 | -6m2 | 3.946 | 4.517 | - | - | - | 3.63 | 3.63 | P-6m2 | 1OsBr2-3 | Yes | 0.97 |
| Hf2I2 | 4/mmm | - | 3.423 | - | - | - | 4.115 | 4.115 | P4/nmm | 2HfI-2 | No | 0.433 |
| Hf2I2 | -3m | - | 3.594 | - | - | 0 | 3.604 | 3.604 | P-3m1 | 2HfI-1 | Yes | 0.175 |
| HfBr2 | -3m | - | 3.701 | - | - | - | 3.437 | 3.437 | P-3m1 | 1HfBr2-2 | No | 0.131 |
| HfBr2 | -6m2 | - | 2.991 | 0.484 | 1.711 | 1.226 | 3.5 | 3.5 | P-6m2 | 1HfBr2-1 | Yes | 0.007 |
| HfBr2 | -42m | - | 4.403 | - | - | - | 3.405 | 3.405 | P-4m2 | 1HfBr2-3 | No | 0.446 |
| ReCl2 | -3m | - | 3.38 | - | - | - | 3.304 | 3.304 | P-3m1 | 1ReCl2-2 | No | 0.891 |
| ReCl2 | -42m | - | 4.21 | - | - | - | 3.038 | 3.038 | P-4m2 | 1ReCl2-1 | No | 0.76 |
| ReCl2 | -6m2 | 1.121 | 4.293 | - | - | - | 3.048 | 3.048 | P-6m2 | 1ReCl2-3 | No | 1.101 |
| Rh2Br4 | 2/m | 1.996 | 4.71 | - | - | - | 3.695 | 7.05 | C2/m | 2RhBr2-1 | Yes | 0.214 |
| TaF2 | -42m | - | 5.576 | - | - | - | 2.758 | 2.758 | P-4m2 | 1TaF2-1 | No | 0.57 |
| ZrI2 | -42m | 1.793 | 3.85 | - | - | - | 3.889 | 3.889 | P-4m2 | 1ZrI2-3 | No | 0.492 |
| ZrI2 | -6m2 | - | 3.051 | 0.855 | 1.894 | 1.038 | 3.829 | 3.829 | P-6m2 | 1ZrI2-1 | Yes | 0.029 |
| ZrI2 | -3m | - | 3.556 | - | - | - | 3.783 | 3.783 | P-3m1 | 1ZrI2-2 | No | 0.138 |
| Co2Cl6 | 6/mmm | 6.179 | 6.707 | - | - | - | 6.358 | 6.358 | P6/mmm | 2CoCl3-2 | No | 0.221 |
| Co2Cl6 | -3m | - | 5.992 | -5.151 | -1.594 | 3.558 | 5.82 | 5.82 | P-31m | 2CoCl3-1 | Yes | 0 |
| Cu2I6 | -6m2 | - | 5.635 | - | - | - | 7.319 | 7.319 | P-62m | 2CuI3-2 | No | 0.201 |
| Cu2I6 | -3m | -1.723 | 5.726 | - | - | - | 6.816 | 6.816 | P-31m | 2CuI3-1 | No | 0.143 |
| Hf2Br6 | -6m2 | 1.984 | 3.991 | - | - | - | 6.465 | 6.465 | P-62m | 2HfBr3-2 | No | 0.035 |
| Hf2Br6 | -3m | 1.955 | 3.919 | - | - | - | 6.521 | 6.521 | P-31m | 2HfBr3-1 | No | 0 |
| Nb2I6 | -3m | 4 | 4.596 | - | - | - | 7.153 | 7.153 | P-31m | 2NbI3-1 | Unknown | 0.104 |
| Nb2I6 | -6m2 | - | 4.201 | - | - | - | 6.781 | 6.781 | P-62m | 2NbI3-2 | Yes | 0.159 |
| Os2Br6 | -3m | 1.996 | 4.967 | - | - | - | 6.479 | 6.479 | P-31m | 2OsBr3-1 | Unknown | 0 |
| Os2Br6 | -6m2 | 2.337 | 4.466 | - | - | - | 6.905 | 6.905 | P-62m | 2OsBr3-2 | No | 0.323 |
| Pd2Br6 | -6m2 | - | 6.158 | - | - | - | 7.016 | 7.016 | P-62m | 2PdBr3-2 | No | 0.237 |
| Pd2Br6 | -3m | 1.988 | 5.865 | - | - | - | 6.659 | 6.659 | P-31m | 2PdBr3-1 | No | 0.015 |
| Ru2Cl6 | 6/mmm | 2.915 | 5.401 | - | - | - | 6.53 | 6.53 | P6/mmm | 2RuCl3-3 | No | 0.34 |
| Ru2Cl6 | -3m | 1.999 | 5.827 | - | - | - | 6.066 | 6.066 | P-31m | 2RuCl3-1 | Yes | 0.021 |
| Sc2Cl6 | 6/mmm | - | 6.185 | -6.275 | -0.97 | 5.305 | 6.775 | 6.775 | P6/mmm | 2ScCl3-3 | No | 0.072 |
| Sc2Cl6 | -3m | - | 6.246 | -6.439 | -0.469 | 5.97 | 6.488 | 6.488 | P-31m | 2ScCl3-1 | Yes | 0 |
| Ag2F4 | 2/m | 1.687 | 7.197 | - | - | - | 3.827 | 5.88 | P2_1/c | 2AgF2-1 | Yes | 0.024 |
| Au2Br2 | mmm | - | 4.365 | -4.385 | -1.459 | 2.926 | 4.315 | 4.315 | Cmme | 2AuBr-1 | Yes | 0 |
| Au2Br2 | -3m | - | 5.643 | -3.296 | -1.98 | 1.317 | 4.253 | 4.257 | P-3m1 | 2AuBr-3 | No | 0.238 |
| Au2Br2 | 4/mmm | - | 5.646 | -3.299 | -2.076 | 1.222 | 4.057 | 4.057 | P4/nmm | 2AuBr-2 | No | 0.235 |
| Ag2Br2 | -3m | - | 5.375 | -4.312 | -1.349 | 2.962 | 4.434 | 4.438 | P-3m1 | 2AgBr-1 | Yes | 0.009 |
| Ag2Br2 | 4/mmm | - | 5.411 | -4.738 | -1.647 | 3.092 | 4.411 | 4.411 | P4/nmm | 2AgBr-3 | Yes | 0.017 |
| Cr2F2 | 4/mmm | 3.154 | 5.16 | - | - | - | 3.272 | 3.272 | P4/nmm | 2CrF-2 | No | 1.275 |
| Cr2F2 | -3m | - | 5.973 | - | - | 0 | 2.706 | 2.707 | P-3m1 | 2CrF-1 | No | 0.358 |
| AgCl2 | -6m2 | 0.981 | 7.29 | - | - | - | 3.667 | 3.667 | P-6m2 | 1AgCl2-3 | No | 0.195 |
| AgCl2 | -3m | 0.975 | 7.089 | - | - | - | 3.862 | 3.862 | P-3m1 | 1AgCl2-1 | No | 0.078 |
| AgCl2 | -42m | - | 7.418 | - | - | - | 3.832 | 3.832 | P-4m2 | 1AgCl2-2 | No | 0.161 |
| AuF2 | -42m | 0.998 | 7.911 | - | - | - | 3.317 | 3.317 | P-4m2 | 1AuF2-4 | No | 0.415 |
| CoF2 | -42m | 3 | 5.642 | - | - | - | 3.308 | 3.308 | P-4m2 | 1CoF2-2 | No | 0.11 |
| CrCl2 | -6m2 | 4 | 5.4 | - | - | - | 3.502 | 3.502 | P-6m2 | 1CrCl2-3 | No | 0.284 |
| CrCl2 | -3m | 4 | 4.814 | - | - | - | 3.649 | 3.649 | P-3m1 | 1CrCl2-1 | No | 0.112 |
| CrCl2 | -42m | 4 | 5.31 | - | - | - | 3.73 | 3.73 | P-4m2 | 1CrCl2-2 | No | 0.21 |
| CuBr2 | -3m | 0.983 | 6.214 | - | - | - | 3.721 | 3.721 | P-3m1 | 1CuBr2-2 | No | 0.054 |
| CuBr2 | -42m | - | 6.469 | - | - | - | 3.698 | 3.698 | P-4m2 | 1CuBr2-1 | Yes | 0.034 |
| CuBr2 | -6m2 | 0.995 | 6.17 | - | - | - | 3.604 | 3.604 | P-6m2 | 1CuBr2-3 | No | 0.203 |
| HgCl2 | -3m | - | 6.399 | -5.677 | -2.078 | 3.599 | 3.994 | 3.994 | P-3m1 | 1HgCl2-1 | No | 0.026 |
| HgCl2 | -6m2 | - | 6.821 | -5.661 | -2.46 | 3.201 | 3.803 | 3.803 | P-6m2 | 1HgCl2-3 | No | 0.131 |
| HgCl2 | -42m | - | 6.458 | -6.382 | -2.674 | 3.708 | 4.123 | 4.123 | P-4m2 | 1HgCl2-2 | Yes | 0.033 |
| MnF2 | -42m | 1.008 | 6.234 | - | - | - | 2.606 | 2.606 | P-4m2 | 1MnF2-2 | Unknown | 0.887 |
| PdCl2 | -42m | - | 6.323 | - | - | - | 3.672 | 3.672 | P-4m2 | 1PdCl2-2 | No | 0.369 |
| PdCl2 | -6m2 | 1.99 | 6.533 | - | - | - | 3.549 | 3.549 | P-6m2 | 1PdCl2-3 | No | 0.447 |
| Pd2Cl4 | mmm | - | 5.985 | - | - | - | 4.237 | 6.457 | Pmna | 2PdCl2-1 | No | 0 |
| PdCl2 | -3m | 1.998 | 6.009 | -4.339 | -2.254 | 2.086 | 3.731 | 3.731 | P-3m1 | 1PdCl2-1 | Yes | 0.166 |
| RhBr2 | -3m | 0.999 | 4.702 | - | - | - | 3.824 | 3.824 | P-3m1 | 1RhBr2-1 | No | 0.229 |
| RhBr2 | -6m2 | 2.973 | 5.541 | - | - | - | 3.725 | 3.725 | P-6m2 | 1RhBr2-3 | No | 0.555 |
| RhBr2 | -42m | - | 5.101 | - | - | - | 3.678 | 3.678 | P-4m2 | 1RhBr2-2 | No | 0.441 |
| WF2 | -42m | 0.421 | 5.512 | - | - | - | 2.694 | 2.694 | P-4m2 | 1WF2-1 | No | 0.737 |
| Ir2Br6 | -6m2 | - | 4.786 | -2.469 | -1.699 | 0.77 | 6.548 | 6.548 | P-62m | 2IrBr3-2 | No | 0.499 |
| Ir2Br6 | -3m | - | 4.73 | -3.369 | -0.476 | 2.893 | 6.471 | 6.471 | P-31m | 2IrBr3-1 | Yes | 0 |
| Re2Cl6 | -6m2 | 1.899 | 4.44 | - | - | - | 5.674 | 5.674 | P-62m | 2ReCl3-2 | No | 0.506 |
| Re2Cl6 | -3m | 1.995 | 4.972 | - | - | - | 5.751 | 5.751 | P-31m | 2ReCl3-1 | No | 0.298 |
| V2Br6 | -6m2 | 4 | 5.177 | - | - | - | 6.913 | 6.913 | P-62m | 2VBr3-2 | No | 0.109 |
| V2Br6 | -3m | 4 | 5.598 | - | - | - | 6.599 | 6.599 | P-31m | 2VBr3-1 | Yes | 0 |
| IrF2 | -42m | 1.243 | 7.332 | - | - | - | 2.655 | 2.655 | P-4m2 | 1IrF2-1 | No | 0.728 |
| MoF2 | -42m | - | 5.761 | - | - | - | 2.695 | 2.695 | P-4m2 | 1MoF2-1 | No | 0.523 |
| PtI2 | -3m | - | 4.468 | - | - | - | 4.086 | 4.086 | P-3m1 | 1PtI2-1 | No | 0.267 |
| PtI2 | -42m | - | 4.911 | - | - | - | 4.086 | 4.086 | P-4m2 | 1PtI2-2 | No | 0.322 |
| PtI2 | -6m2 | - | 4.576 | - | - | - | 3.928 | 3.928 | P-6m2 | 1PtI2-3 | No | 0.504 |
| Pt4I8 | 2/m | - | 4.752 | -4.611 | -2.399 | 2.212 | 7.157 | 9.207 | P2_1/c | 4PtI2-1 | Yes | 0 |
| Sc2Cl4 | 2/m | - | 3.642 | - | - | - | 3.58 | 6.196 | P2_1/m | 2ScCl2-1 | No | 0.027 |
| Ta2I4 | 2/m | - | 2.815 | - | - | - | 3.746 | 6.529 | P2_1/m | 2TaI2-1 | Yes | 0.223 |
| TiCl2 | -42m | 2 | 4.459 | - | - | - | 3.46 | 3.46 | P-4m2 | 1TiCl2-4 | No | 0.355 |
| TiCl2 | -6m2 | - | 3.794 | -0.241 | 1.154 | 1.395 | 3.279 | 3.279 | P-6m2 | 1TiCl2-1 | Yes | 0.007 |
| TiCl2 | -3m | - | 4.11 | - | - | - | 3.291 | 3.291 | P-3m1 | 1TiCl2-2 | No | 0.113 |
| ZnCl2 | -42m | - | 5.676 | -6.54 | -0.844 | 5.696 | 3.726 | 3.726 | P-4m2 | 1ZnCl2-1 | Yes | 0 |
| ZnCl2 | -3m | - | 5.634 | -5.958 | -0.029 | 5.929 | 3.599 | 3.599 | P-3m1 | 1ZnCl2-2 | Yes | 0.062 |
| ZnCl2 | -6m2 | - | 5.97 | -5.44 | -0.722 | 4.718 | 3.48 | 3.48 | P-6m2 | 1ZnCl2-3 | No | 0.236 |
| Y2Cl4 | 2/m | - | 3.566 | - | - | - | 3.755 | 6.517 | P2_1/m | 2YCl2-1 | No | 0.113 |
| Co2I6 | -3m | - | 4.874 | -2.599 | -0.723 | 1.876 | 6.745 | 6.745 | P-31m | 2CoI3-1 | Yes | 0 |
| Co2I6 | -6m2 | 5.407 | 5.319 | - | - | - | 7.292 | 7.292 | P-62m | 2CoI3-3 | No | 0.205 |
| Fe2Br6 | -6m2 | 9.904 | 6.294 | - | - | - | 6.728 | 6.728 | P-62m | 2FeBr3-2 | No | 0.006 |
| Fe2Br6 | -3m | 2 | 5.423 | - | - | - | 6.293 | 6.293 | P-31m | 2FeBr3-1 | Unknown | 0 |
| Hf2Cl6 | -3m | 1.922 | 4.092 | - | - | - | 6.166 | 6.166 | P-31m | 2HfCl3-1 | No | 0.091 |
| Hf2Cl6 | -6m2 | 1.983 | 4.162 | - | - | - | 6.147 | 6.147 | P-62m | 2HfCl3-2 | No | 0.119 |
| Mo2I6 | -3m | 6 | 4.719 | -2.635 | -0.126 | 2.509 | 7.187 | 7.187 | P-31m | 2MoI3-1 | Yes | 0.101 |
| Os2Cl6 | 6/mmm | 2.141 | 4.738 | - | - | - | 6.491 | 6.491 | P6/mmm | 2OsCl3-2 | No | 0.424 |
| Os2Cl6 | -3m | 1.998 | 5.267 | - | - | - | 6.059 | 6.059 | P-31m | 2OsCl3-1 | Yes | 0.062 |
| Pd2Cl6 | -3m | 1.996 | 6.343 | - | - | - | 6.338 | 6.338 | P-31m | 2PdCl3-1 | No | 0 |
| Pd2Cl6 | 6/mmm | 3.006 | 6.821 | - | - | - | 6.692 | 6.692 | P6/mmm | 2PdCl3-3 | No | 0.261 |
| Ru2I6 | -6m2 | - | 4.513 | - | - | - | 6.959 | 6.959 | P-62m | 2RuI3-2 | No | 0.228 |
| Ru2I6 | -3m | 1.945 | 5.135 | - | - | - | 7.014 | 7.014 | P-31m | 2RuI3-1 | Yes | 0 |
| Sc2I6 | -3m | - | 5.002 | -3.413 | 0.016 | 3.428 | 7.353 | 7.353 | P-31m | 2ScI3-1 | Yes | 0 |
| Sc2I6 | -6m2 | - | 5.127 | - | - | - | 7.556 | 7.556 | P-62m | 2ScI3-4 | No | 0.092 |
| Ti2Br6 | -6m2 | 2 | 4.819 | -2.289 | -1.217 | 1.071 | 6.594 | 6.594 | P-62m | 2TiBr3-2 | Yes | 0.065 |
| Ag2Cl2 | 4/mmm | - | 5.718 | -5.518 | -2.056 | 3.462 | 4.477 | 4.477 | P4/nmm | 2AgCl-7 | No | 0.048 |
| Ag2Cl2 | -3m | - | 5.036 | -4.353 | -1.303 | 3.05 | 4.4 | 4.414 | P-3m1 | 2AgCl-2 | Yes | 0.018 |
| Au2I4 | 2/m | - | 5.429 | - | - | - | 4.499 | 7.117 | C2/m | 2AuI2-2 | No | 0.106 |
| Cr2I2 | -3m | 0.104 | 3.642 | - | - | - | 3.737 | 3.737 | P-3m1 | 2CrI-1 | Unknown | 0.699 |
| CoI2 | -3m | 1.002 | 4.696 | - | - | - | 3.885 | 3.885 | P-3m1 | 1CoI2-2 | Yes | 0.112 |
| CoI2 | -42m | 3 | 5.273 | - | - | - | 4.182 | 4.182 | P-4m2 | 1CoI2-1 | Yes | 0.072 |
| CoI2 | -6m2 | 2.999 | 5.08 | - | - | - | 3.859 | 3.859 | P-6m2 | 1CoI2-3 | Unknown | 0.257 |
| FeCl2 | -42m | 4 | 5.332 | - | - | - | 3.801 | 3.801 | P-4m2 | 1FeCl2-2 | Yes | 0.037 |
| FeCl2 | -6m2 | 4 | 5.569 | -1.477 | -1.406 | 0.071 | 3.365 | 3.365 | P-6m2 | 1FeCl2-3 | Yes | 0.067 |
| YCl2 | -6m2 | 0.998 | 3.901 | -0.746 | 0.253 | 0.999 | 3.73 | 3.73 | P-6m2 | 1YCl2-1 | Yes | 0.046 |
| YCl2 | -3m | - | 3.518 | - | - | - | 3.746 | 3.746 | P-3m1 | 1YCl2-2 | No | 0.115 |
| YCl2 | -42m | 0.768 | 4.356 | - | - | - | 3.702 | 3.702 | P-4m2 | 1YCl2-3 | No | 0.425 |
| MnI2 | -6m2 | 5 | 4.988 | - | - | - | 4.036 | 4.036 | P-6m2 | 1MnI2-3 | No | 0.135 |
| MnI2 | -3m | 5 | 4.715 | -2.687 | 0.624 | 3.311 | 4.166 | 4.166 | P-3m1 | 1MnI2-1 | Yes | 0 |
| MnI2 | -42m | 5 | 4.793 | -3.786 | -0.151 | 3.636 | 4.346 | 4.346 | P-4m2 | 1MnI2-2 | Yes | 0.012 |
| NbBr2 | -3m | - | 3.777 | - | - | - | 3.379 | 3.379 | P-3m1 | 1NbBr2-1 | No | 0.243 |
| NbBr2 | -42m | - | 4.312 | - | - | - | 3.304 | 3.304 | P-4m2 | 1NbBr2-3 | No | 0.542 |
| NbBr2 | -6m2 | - | 3.403 | - | - | - | 3.355 | 3.355 | P-6m2 | 1NbBr2-2 | No | 0.319 |
| ReF2 | -42m | - | 6.046 | - | - | - | 2.649 | 2.649 | P-4m2 | 1ReF2-1 | No | 0.575 |
| ScCl2 | -42m | 1 | 4.058 | - | - | - | 3.661 | 3.661 | P-4m2 | 1ScCl2-3 | No | 0.334 |
| ScCl2 | -6m2 | 0.981 | 4.057 | -0.521 | 0.215 | 0.736 | 3.518 | 3.518 | P-6m2 | 1ScCl2-1 | Yes | 0.009 |
| ScCl2 | -3m | - | 3.58 | - | - | - | 3.581 | 3.581 | P-3m1 | 1ScCl2-2 | No | 0.028 |
| TaI2 | -3m | - | 3.288 | - | - | - | 3.646 | 3.646 | P-3m1 | 1TaI2-1 | No | 0.469 |
| TaI2 | -42m | - | 3.835 | - | - | - | 3.598 | 3.598 | P-4m2 | 1TaI2-3 | No | 0.729 |
| TaI2 | -6m2 | - | 3.009 | - | - | - | 3.58 | 3.58 | P-6m2 | 1TaI2-2 | No | 0.506 |
| WI2 | -6m2 | - | 3.241 | - | - | - | 3.459 | 3.459 | P-6m2 | 1WI2-3 | No | 0.851 |
| WI2 | -3m | - | 3.244 | - | - | - | 3.692 | 3.692 | P-3m1 | 1WI2-1 | No | 0.664 |
| WI2 | -42m | - | 3.698 | - | - | - | 3.535 | 3.535 | P-4m2 | 1WI2-2 | No | 0.783 |
| Ir2Cl6 | -3m | - | 5.004 | -3.806 | -0.504 | 3.302 | 6.13 | 6.13 | P-31m | 2IrCl3-1 | Yes | 0 |
| Ir2Cl6 | -3m | - | 5.399 | -3.602 | -2.37 | 1.232 | 6.561 | 6.561 | P-3m1 | 2IrCl3-2 | No | 0.555 |
| Re2I6 | 6/mmm | - | 3.777 | -1.026 | -0.804 | 0.221 | 7.494 | 7.494 | P6/mmm | 2ReI3-2 | No | 0.425 |
| Re2I6 | -3m | 4 | 4.556 | - | - | - | 6.987 | 6.987 | P-31m | 2ReI3-1 | Yes | 0.27 |
| Ti2Cl6 | -6m2 | 2 | 4.995 | - | - | - | 6.236 | 6.236 | P-62m | 2TiCl3-3 | Yes | 0.071 |
| V2Cl6 | -3m | 4 | 5.886 | - | - | - | 6.224 | 6.224 | P-31m | 2VCl3-1 | Yes | 0 |
| V2Cl6 | 6/mmm | 4 | 5.425 | - | - | - | 6.513 | 6.513 | P6/mmm | 2VCl3-2 | No | 0.109 |
| Y2Br6 | 6/mmm | - | 5.425 | -5.547 | -0.638 | 4.91 | 7.564 | 7.564 | P6/mmm | 2YBr3-3 | No | 0.051 |
| Y2Br6 | -3m | - | 5.392 | -5.581 | -0.103 | 5.478 | 7.247 | 7.247 | P-31m | 2YBr3-1 | Yes | 0 |
| Cu2F4 | 2/m | 2 | 7.325 | -8.832 | -8.235 | 0.596 | 3.416 | 5.28 | P2_1/c | 2CuF2-1 | Yes | 0.011 |
| NbF4 | 4/mmm | 0.602 | 5.129 | - | - | - | 4.099 | 4.099 | P4/mmm | 1NbF4-1 | No | 0.014 |
| AgF2 | 4/mmm | - | 6.481 | - | - | - | 4.113 | 4.113 | P4/mmm | 1AgF2-1 | No | 0.031 |
| AuI2 | -42m | - | 5.65 | - | - | - | 4.187 | 4.187 | P-4m2 | 1AuI2-1 | Yes | 0.105 |
| AuI2 | -6m2 | 0.916 | 5.447 | - | - | - | 4.08 | 4.08 | P-6m2 | 1AuI2-3 | No | 0.271 |
| AuI2 | -3m | - | 5.462 | - | - | - | 4.226 | 4.226 | P-3m1 | 1AuI2-2 | No | 0.144 |
| CrF2 | -42m | 1.996 | 6.21 | - | - | - | 2.688 | 2.688 | P-4m2 | 1CrF2-3 | Unknown | 0.714 |
| CuCl2 | -6m2 | 1 | 6.848 | - | - | - | 3.411 | 3.411 | P-6m2 | 1CuCl2-3 | No | 0.224 |
| CuCl2 | -3m | 1 | 6.701 | - | - | - | 3.54 | 3.54 | P-3m1 | 1CuCl2-1 | No | 0.057 |
| CuCl2 | -42m | - | 7.067 | - | - | - | 3.562 | 3.562 | P-4m2 | 1CuCl2-2 | Yes | 0.061 |
| HfCl2 | -42m | - | 4.858 | - | - | - | 3.244 | 3.244 | P-4m2 | 1HfCl2-3 | No | 0.448 |
| HfCl2 | -6m2 | - | 3.245 | 0.157 | 1.628 | 1.471 | 3.349 | 3.349 | P-6m2 | 1HfCl2-1 | Yes | 0.007 |
| HfCl2 | -3m | - | 4.085 | - | - | - | 3.276 | 3.276 | P-3m1 | 1HfCl2-2 | No | 0.135 |
| HgF2 | -42m | - | 7.029 | -8.066 | -4.191 | 3.875 | 3.94 | 3.94 | P-4m2 | 1HgF2-2 | No | 0.162 |
| IrI2 | -3m | - | 3.768 | - | - | - | 4.039 | 4.039 | P-3m1 | 1IrI2-1 | No | 0.386 |
| IrI2 | -42m | - | 4.332 | - | - | - | 3.941 | 3.941 | P-4m2 | 1IrI2-2 | No | 0.584 |
| IrI2 | -6m2 | - | 4.064 | - | - | - | 3.878 | 3.878 | P-6m2 | 1IrI2-3 | No | 0.809 |
| Mo2I4 | 2/m | 0.919 | 3.318 | - | - | - | 4.103 | 6.329 | P2_1/m | 2MoI2-1 | Yes | 0.546 |
| NiBr2 | -6m2 | 2 | 5.598 | - | - | - | 3.569 | 3.569 | P-6m2 | 1NiBr2-3 | No | 0.294 |
| NiBr2 | -42m | 1.999 | 5.86 | - | - | - | 3.916 | 3.916 | P-4m2 | 1NiBr2-2 | Yes | 0.14 |
| NiBr2 | -3m | 2 | 5.572 | -4.25 | -0.933 | 3.317 | 3.699 | 3.699 | P-3m1 | 1NiBr2-1 | Yes | 0 |
| OsCl2 | -3m | - | 3.799 | -1.857 | 0.856 | 2.714 | 3.645 | 3.645 | P-3m1 | 1OsCl2-1 | Yes | 0.5 |
| OsCl2 | -6m2 | 3.971 | 5.003 | - | - | - | 3.424 | 3.424 | P-6m2 | 1OsCl2-3 | Yes | 1.06 |
| OsCl2 | -42m | - | 4.07 | - | - | - | 3.149 | 3.149 | P-4m2 | 1OsCl2-2 | No | 0.658 |
| PdF2 | -42m | 2 | 8.13 | - | - | - | 2.999 | 2.999 | P-4m2 | 1PdF2-3 | No | 0.453 |
| Rh2Cl4 | 2/m | 1.998 | 5.077 | -3.275 | -1.248 | 2.027 | 3.501 | 6.853 | C2/m | 2RhCl2-1 | Yes | 0.227 |
| RuBr2 | -42m | - | 4.658 | - | - | - | 3.489 | 3.489 | P-4m2 | 1RuBr2-2 | No | 0.503 |
| RuBr2 | -6m2 | 3.988 | 5.332 | - | - | - | 3.65 | 3.65 | P-6m2 | 1RuBr2-3 | Yes | 0.558 |
| RuBr2 | -3m | - | 4.19 | -2.351 | 0.19 | 2.542 | 3.78 | 3.78 | P-3m1 | 1RuBr2-1 | Yes | 0.187 |
| VBr2 | -42m | 3 | 4.589 | - | - | - | 3.789 | 3.789 | P-4m2 | 1VBr2-2 | No | 0.266 |
| VBr2 | -3m | 3 | 3.973 | -2.827 | 0.838 | 3.665 | 3.84 | 3.84 | P-3m1 | 1VBr2-1 | Yes | 0 |
| VBr2 | -6m2 | 1.002 | 3.835 | - | - | - | 3.44 | 3.44 | P-6m2 | 1VBr2-3 | No | 0.361 |
| Fe2Cl6 | -6m2 | 9.987 | 6.991 | - | - | - | 6.431 | 6.431 | P-62m | 2FeCl3-4 | No | 0.093 |
| Fe2Cl6 | -3m | 2 | 5.911 | - | - | - | 5.909 | 5.909 | P-31m | 2FeCl3-2 | Yes | 0.09 |
| Hf2I6 | -3m | 1.97 | 3.834 | - | - | - | 7.083 | 7.083 | P-31m | 2HfI3-1 | No | 0.103 |
| Hf2I6 | -6m2 | 1.951 | 3.779 | - | - | - | 7.044 | 7.044 | P-62m | 2HfI3-2 | Yes | 0.151 |
| Os2I6 | -3m | 1.885 | 4.728 | - | - | - | 7.031 | 7.031 | P-31m | 2OsI3-1 | Yes | 0.176 |
| Os2I6 | -6m2 | 0.88 | 4.136 | - | - | - | 7.111 | 7.111 | P-62m | 2OsI3-2 | No | 0.45 |
| Pd2I6 | -3m | 1.955 | 5.281 | - | - | - | 7.111 | 7.111 | P-31m | 2PdI3-1 | No | 0.061 |
| Pd2I6 | -6m2 | - | 5.371 | - | - | - | 7.571 | 7.571 | P-62m | 2PdI3-2 | No | 0.222 |
| Pd4I8 | 2/m | - | 5.257 | -5.129 | -3.111 | 2.017 | 7.169 | 9.103 | P2_1/c | 4PdI2-1 | Yes | 0 |
| Ag2F2 | 4/mmm | - | 4.81 | -5.273 | -2.798 | 2.475 | 4.693 | 4.693 | P4/nmm | 2AgF-3 | No | 0.101 |
| Ag2F2 | 1 | - | 5.664 | -4.718 | -2.908 | 1.81 | 3.078 | 4.161 | P1 | 2AgF-2 | No | 0.048 |
| Hg2I2 | 4/mmm | 0.887 | 5.546 | - | - | - | 4.504 | 4.504 | P4/nmm | 2HgI-3 | No | 0.396 |
| Hg2I2 | -3m | - | 5.273 | -2.431 | -0.513 | 1.918 | 4.456 | 4.483 | P-3m1 | 2HgI-1 | Yes | 0 |
| HgI2 | -6m2 | - | 5.387 | -2.87 | -1.5 | 1.37 | 4.253 | 4.253 | P-6m2 | 1HgI2-4 | No | 0.189 |
| HgI2 | -42m | - | 5.164 | -4.02 | -1.623 | 2.397 | 4.524 | 4.524 | P-4m2 | 1HgI2-1 | Yes | 0.006 |
| HgI2 | -3m | - | 5.269 | -3.195 | -1.275 | 1.919 | 4.387 | 4.387 | P-3m1 | 1HgI2-3 | No | 0.077 |
| Hg4I8 | -42m | - | 5.333 | -5.45 | -2.96 | 2.49 | 9.083 | 9.083 | P-4m2 | 4HgI2-1 | Yes | 0 |
| MoI2 | -3m | 2 | 3.64 | - | - | - | 4.02 | 4.02 | P-3m1 | 1MoI2-1 | Yes | 0.599 |
| MoI2 | -6m2 | 2 | 3.66 | - | - | - | 3.825 | 3.825 | P-6m2 | 1MoI2-2 | No | 0.786 |
| MoI2 | -42m | 1.606 | 4.154 | - | - | - | 3.731 | 3.731 | P-4m2 | 1MoI2-3 | No | 0.868 |
| PdI2 | -42m | - | 5.442 | - | - | - | 4.057 | 4.057 | P-4m2 | 1PdI2-2 | No | 0.269 |
| PdI2 | -3m | - | 5.003 | - | - | - | 4.064 | 4.064 | P-3m1 | 1PdI2-1 | No | 0.168 |
| PdI2 | -6m2 | - | 4.942 | - | - | - | 3.905 | 3.905 | P-6m2 | 1PdI2-3 | No | 0.353 |
| Re2I4 | 2/m | 1.946 | 3.623 | - | - | - | 4.154 | 6.69 | P2_1/m | 2ReI2-1 | Yes | 0.71 |
| RhCl2 | -42m | - | 5.421 | - | - | - | 3.49 | 3.49 | P-4m2 | 1RhCl2-2 | No | 0.452 |
| RhCl2 | -6m2 | 2.999 | 5.977 | - | - | - | 3.554 | 3.554 | P-6m2 | 1RhCl2-3 | No | 0.579 |
| RhCl2 | -3m | 1 | 5.033 | - | - | - | 3.654 | 3.654 | P-3m1 | 1RhCl2-1 | No | 0.269 |
| TiF2 | -42m | - | 6.2 | - | - | - | 2.732 | 2.732 | P-4m2 | 1TiF2-3 | No | 0.535 |
| ZnF2 | -42m | - | 6.642 | -9.275 | -2.364 | 6.911 | 3.342 | 3.342 | P-4m2 | 1ZnF2-2 | Yes | 0.112 |
| ZrBr2 | -3m | - | 3.883 | - | - | - | 3.5 | 3.5 | P-3m1 | 1ZrBr2-2 | No | 0.105 |
| ZrBr2 | -42m | - | 4.512 | - | - | - | 3.456 | 3.456 | P-4m2 | 1ZrBr2-3 | No | 0.462 |
| ZrBr2 | -6m2 | - | 3.238 | 0.193 | 1.469 | 1.276 | 3.562 | 3.562 | P-6m2 | 1ZrBr2-1 | Yes | 0 |
| Ir2I6 | -6m2 | - | 4.253 | -1.487 | -0.731 | 0.756 | 7.041 | 7.041 | P-62m | 2IrI3-2 | No | 0.42 |
| Ir2I6 | -3m | - | 4.439 | -2.398 | 0.051 | 2.45 | 6.947 | 6.947 | P-31m | 2IrI3-1 | Yes | 0 |
| Rh2Br6 | 32 | - | 5.469 | -3.498 | -2.368 | 1.129 | 6.641 | 6.641 | P321 | 2RhBr3-2 | No | 0.408 |
| Rh2Br6 | -3m | - | 5.363 | -4.071 | -1.334 | 2.737 | 6.437 | 6.437 | P-31m | 2RhBr3-1 | Yes | 0 |
| Ti2I6 | -6m2 | 2 | 4.668 | -1.85 | -0.709 | 1.141 | 7.154 | 7.154 | P-62m | 2TiI3-2 | Yes | 0.083 |
| V2I6 | -3m | 4 | 5.265 | - | - | - | 7.153 | 7.153 | P-31m | 2VI3-1 | Yes | 0.005 |
| V2I6 | -6m2 | 4 | 4.897 | - | - | - | 7.347 | 7.347 | P-62m | 2VI3-2 | No | 0.113 |
| Y2Cl6 | -3m | - | 5.821 | -6.741 | -0.015 | 6.726 | 6.966 | 6.966 | P-31m | 2YCl3-1 | Yes | 0 |
| Y2Cl6 | 6/mmm | - | 5.835 | -6.549 | -0.535 | 6.014 | 7.203 | 7.203 | P6/mmm | 2YCl3-3 | No | 0.042 |
| Ag2I4 | 2/m | - | 5.843 | - | - | - | 4.392 | 6.975 | C2/m | 2AgI2-2 | No | 0.104 |
| CrI2 | -6m2 | 4 | 4.668 | - | - | - | 3.971 | 3.971 | P-6m2 | 1CrI2-3 | No | 0.257 |
| CrI2 | -42m | 4 | 5.008 | - | - | - | 4.197 | 4.197 | P-4m2 | 1CrI2-2 | Yes | 0.192 |
| CrI2 | -3m | 4 | 4.47 | - | - | - | 4.116 | 4.116 | P-3m1 | 1CrI2-1 | No | 0.094 |
| FeF2 | -42m | - | 6.316 | - | - | - | 2.653 | 2.653 | P-4m2 | 1FeF2-3 | No | 0.719 |
| Nb2Cl4 | 2/m | - | 3.399 | - | - | - | 3.295 | 5.918 | P2_1/m | 2NbCl2-1 | No | 0.122 |
| ReI2 | -3m | 0.998 | 3.836 | - | - | - | 4.08 | 4.08 | P-3m1 | 1ReI2-1 | Unknown | 0.725 |
| ReI2 | -6m2 | 2.93 | 3.539 | - | - | - | 3.833 | 3.833 | P-6m2 | 1ReI2-3 | No | 1.062 |
| ReI2 | -42m | 0.86 | 3.734 | - | - | - | 3.633 | 3.633 | P-4m2 | 1ReI2-2 | No | 0.945 |
| ScF2 | -42m | 0.743 | 4.266 | - | - | - | 3.125 | 3.125 | P-4m2 | 1ScF2-2 | No | 0.623 |
| YF2 | -42m | - | 3.716 | - | - | - | 3.391 | 3.391 | P-4m2 | 1YF2-3 | No | 0.588 |
| ZnI2 | -3m | - | 4.951 | -3.104 | -0.509 | 2.595 | 4.1 | 4.1 | P-3m1 | 1ZnI2-2 | Yes | 0.124 |
| ZnI2 | -6m2 | - | 4.938 | -2.453 | -0.809 | 1.644 | 4.011 | 4.011 | P-6m2 | 1ZnI2-3 | No | 0.305 |
| ZnI2 | -42m | - | 4.625 | -3.893 | -0.474 | 3.419 | 4.226 | 4.226 | P-4m2 | 1ZnI2-1 | Yes | 0.003 |
| Ag2Br6 | 6/mmm | - | 6.733 | - | - | - | 7.134 | 7.134 | P6/mmm | 2AgBr3-6 | No | 0.197 |
| Ag2Br6 | -3m | 3.733 | 6.522 | -4.852 | -4.084 | 0.768 | 6.843 | 6.843 | P-31m | 2AgBr3-5 | Yes | 0.131 |
| Fe2I6 | -3m | 2 | 4.923 | - | - | - | 6.827 | 6.827 | P-31m | 2FeI3-1 | Yes | 0.02 |
| Fe2I6 | -6m2 | 8.811 | 5.517 | - | - | - | 7.284 | 7.284 | P-62m | 2FeI3-3 | Unknown | 0.074 |
| Ni2Br6 | 6/mmm | 3.981 | 6.168 | - | - | - | 6.759 | 6.759 | P6/mmm | 2NiBr3-3 | No | 0.19 |
| Ni2Br6 | -3m | 2 | 6.126 | - | - | - | 6.334 | 6.334 | P-31m | 2NiBr3-1 | No | 0.001 |
| Pt2Br6 | -6m2 | 1.072 | 5.677 | - | - | - | 7.006 | 7.006 | P-62m | 2PtBr3-2 | Unknown | 0.321 |
| Pt2Br6 | -3m | 1.979 | 5.246 | - | - | - | 6.725 | 6.725 | P-31m | 2PtBr3-1 | No | 0.04 |
| Ag2I2 | -3m | - | 4.531 | -2.815 | -0.033 | 2.782 | 4.575 | 4.579 | P-3m1 | 2AgI-1 | Yes | 0.01 |
| Ag2I2 | 4/mmm | - | 4.523 | -3.282 | -0.36 | 2.922 | 4.528 | 4.528 | P4/nmm | 2AgI-2 | No | 0.016 |
| AgI2 | -42m | - | 5.944 | - | - | - | 4.04 | 4.04 | P-4m2 | 1AgI2-2 | No | 0.117 |
| AgI2 | -3m | - | 5.889 | - | - | - | 4.191 | 4.191 | P-3m1 | 1AgI2-1 | No | 0.115 |
| AgI2 | -6m2 | - | 5.916 | - | - | - | 4.001 | 4.001 | P-6m2 | 1AgI2-3 | No | 0.205 |
| CdBr2 | -3m | - | 5.593 | -4.99 | -0.831 | 4.159 | 4.062 | 4.062 | P-3m1 | 1CdBr2-1 | Yes | 0 |
| CdBr2 | -6m2 | - | 5.801 | -4.602 | -1.228 | 3.373 | 3.914 | 3.914 | P-6m2 | 1CdBr2-3 | No | 0.121 |
| CdBr2 | -42m | - | 5.627 | -5.672 | -1.585 | 4.087 | 4.215 | 4.215 | P-4m2 | 1CdBr2-2 | Yes | 0.02 |
| CuF2 | -42m | - | 6.235 | - | - | 0 | 3.73 | 3.729 | P-4m2 | 1CuF2-2 | No | 0.091 |
| HfF2 | -42m | - | 5.833 | - | - | - | 2.913 | 2.913 | P-4m2 | 1HfF2-3 | No | 0.682 |
| NbCl2 | -42m | - | 4.723 | - | - | - | 3.12 | 3.12 | P-4m2 | 1NbCl2-3 | No | 0.495 |
| NbCl2 | -6m2 | - | 3.733 | - | - | - | 3.191 | 3.191 | P-6m2 | 1NbCl2-2 | No | 0.288 |
| NbCl2 | -3m | - | 4.053 | - | - | - | 3.197 | 3.197 | P-3m1 | 1NbCl2-1 | No | 0.19 |
| NiCl2 | -6m2 | 2 | 6.101 | - | - | - | 3.369 | 3.369 | P-6m2 | 1NiCl2-3 | Unknown | 0.322 |
| NiCl2 | -3m | 2 | 6.004 | -5.042 | -0.96 | 4.082 | 3.502 | 3.502 | P-3m1 | 1NiCl2-1 | Yes | 0 |
| NiCl2 | -42m | - | 5.841 | -4.674 | -4.65 | 0.024 | 4.091 | 4.091 | P-4m2 | 1NiCl2-2 | Yes | 0.227 |
| OsF2 | -42m | - | 6.219 | - | - | - | 2.625 | 2.625 | P-4m2 | 1OsF2-1 | No | 0.77 |
| RuCl2 | -6m2 | 3.998 | 5.715 | -2.391 | -1.483 | 0.908 | 3.468 | 3.468 | P-6m2 | 1RuCl2-3 | Yes | 0.565 |
| RuCl2 | -3m | - | 4.364 | -2.592 | 0.233 | 2.824 | 3.593 | 3.593 | P-3m1 | 1RuCl2-1 | Yes | 0.206 |
| RuCl2 | -42m | - | 4.862 | - | - | - | 3.27 | 3.27 | P-4m2 | 1RuCl2-2 | No | 0.463 |
| Ti2I4 | 2/m | - | 4.038 | 0.137 | 0.559 | 0.423 | 3.75 | 6.672 | P2_1/m | 2TiI2-1 | Yes | 0.004 |
| V2Cl4 | 2/m | 0.846 | 3.923 | - | - | - | 3.257 | 5.684 | P2_1/m | 2VCl2-1 | Yes | 0.11 |
| Y2I4 | 2/m | - | 3.305 | - | - | - | 4.148 | 7.218 | P2_1/m | 2YI2-1 | No | 0.039 |
| Cr2Br6 | -3m | 6 | 5.583 | -4.665 | -1.235 | 3.431 | 6.447 | 6.447 | P-31m | 2CrBr3-1 | Yes | 0 |
| Rh2Cl6 | -3m | - | 6.094 | - | - | - | 6.597 | 6.597 | P-3m1 | 2RhCl3-2 | No | 0.464 |
| Rh2Cl6 | -3m | - | 5.751 | -4.647 | -1.456 | 3.19 | 6.09 | 6.09 | P-31m | 2RhCl3-1 | Yes | 0 |
| Ta2Br6 | -6m2 | - | 4.297 | - | - | - | 6.129 | 6.129 | P-62m | 2TaBr3-2 | No | 0.231 |
| Ta2Br6 | -3m | 1.958 | 4.33 | - | - | - | 6.16 | 6.16 | P-31m | 2TaBr3-1 | No | 0.205 |
| Y2I6 | -3m | - | 4.847 | -3.816 | 0.072 | 3.889 | 7.709 | 7.709 | P-31m | 2YI3-1 | Yes | 0.001 |
| Y2I6 | -6m2 | - | 4.992 | -4.186 | -0.432 | 3.754 | 8.082 | 8.082 | P-62m | 2YI3-2 | No | 0.065 |
| Cu2F2 | 4/mmm | - | 6.333 | -5.937 | -5.46 | 0.477 | 3.577 | 3.577 | P4/nmm | 2CuF-2 | No | 0.452 |
| Cu2F2 | -3m | - | 8.147 | - | - | 0 | 2.921 | 2.923 | P-3m1 | 2CuF-1 | No | 0.219 |
| FeI2 | -42m | 4 | 5.01 | - | - | - | 4.273 | 4.273 | P-4m2 | 1FeI2-2 | Yes | 0.004 |
| FeI2 | -6m2 | 4 | 4.921 | -0.532 | -0.512 | 0.02 | 3.872 | 3.872 | P-6m2 | 1FeI2-3 | Yes | 0.112 |
| ScI2 | -42m | 1 | 3.434 | - | - | - | 4.338 | 4.338 | P-4m2 | 1ScI2-3 | Unknown | 0.264 |
| ScI2 | -3m | - | 3.289 | - | - | - | 4.061 | 4.061 | P-3m1 | 1ScI2-1 | No | 0.005 |
| ScI2 | -6m2 | 0.971 | 3.688 | 0.227 | 0.8 | 0.573 | 3.979 | 3.979 | P-6m2 | 1ScI2-2 | Yes | 0.008 |
| PtBr2 | -6m2 | - | 5.253 | - | - | - | 3.717 | 3.717 | P-6m2 | 1PtBr2-3 | No | 0.578 |
| PtBr2 | -3m | 1.967 | 4.938 | -2.772 | -1.421 | 1.352 | 3.954 | 3.954 | P-3m1 | 1PtBr2-1 | Yes | 0.293 |
| PtBr2 | -42m | - | 5.279 | - | - | - | 3.856 | 3.856 | P-4m2 | 1PtBr2-2 | No | 0.412 |
| RhF2 | -42m | 1.939 | 8.05 | - | - | - | 2.747 | 2.747 | P-4m2 | 1RhF2-2 | No | 0.618 |
| Ta2Br4 | 2/m | - | 2.679 | - | - | - | 3.497 | 6.157 | P2_1/m | 2TaBr2-1 | Yes | 0.304 |
| TiI2 | -6m2 | - | 3.33 | 0.793 | 1.622 | 0.828 | 3.769 | 3.769 | P-6m2 | 1TiI2-2 | Yes | 0.052 |
| TiI2 | -42m | 2 | 3.944 | - | - | - | 4.042 | 4.042 | P-4m2 | 1TiI2-3 | No | 0.27 |
| TiI2 | -3m | 2 | 3.728 | - | - | - | 4.083 | 4.083 | P-3m1 | 1TiI2-1 | No | 0.044 |
| VCl2 | -6m2 | 1 | 4.116 | - | - | - | 3.221 | 3.221 | P-6m2 | 1VCl2-3 | No | 0.301 |
| VCl2 | -3m | 3 | 4.007 | -2.964 | 0.929 | 3.894 | 3.66 | 3.66 | P-3m1 | 1VCl2-1 | Yes | 0 |
| VCl2 | -42m | 3 | 4.738 | - | - | - | 3.552 | 3.552 | P-4m2 | 1VCl2-2 | No | 0.263 |
| YI2 | -42m | 0.979 | 3.781 | - | - | - | 4.153 | 4.153 | P-4m2 | 1YI2-3 | No | 0.358 |
| YI2 | -3m | - | 3.264 | - | - | - | 4.151 | 4.151 | P-3m1 | 1YI2-2 | No | 0.04 |
| YI2 | -6m2 | 0.981 | 3.583 | 0.171 | 0.715 | 0.544 | 4.105 | 4.105 | P-6m2 | 1YI2-1 | Yes | 0.003 |
| Ag2Cl6 | 6/mmm | 1.445 | 7.498 | - | - | - | 6.815 | 6.815 | P6/mmm | 2AgCl3-2 | No | 0.229 |
| Ag2Cl6 | -3m | 3.919 | 7.184 | -5.778 | -4.5 | 1.278 | 6.476 | 6.476 | P-31m | 2AgCl3-1 | Yes | 0.123 |
| Cr2Cl6 | -3m | 6 | 5.902 | -5.272 | -1.225 | 4.047 | 6.061 | 6.061 | P-31m | 2CrCl3-1 | Yes | 0 |
| Ni2Cl6 | 6/mmm | 4.787 | 6.902 | - | - | - | 6.384 | 6.384 | P6/mmm | 2NiCl3-2 | No | 0.208 |
| Ni2Cl6 | -3m | 2 | 6.72 | - | - | - | 5.969 | 5.969 | P-31m | 2NiCl3-1 | No | 0 |
| Pt2Cl6 | -3m | 1.992 | 5.586 | - | - | - | 6.415 | 6.415 | P-31m | 2PtCl3-1 | No | 0.079 |
| Pt2Cl6 | -3m | 2.062 | 6.194 | - | - | - | 6.661 | 6.661 | P-3m1 | 2PtCl3-2 | No | 0.397 |
| Co2F2 | -3m | 4.743 | 6.939 | - | - | - | 2.73 | 2.729 | P-3m1 | 2CoF-1 | Yes | 0.166 |
| Co2F2 | 4/mmm | 4.629 | 5.597 | - | - | - | 3.408 | 3.408 | P4/nmm | 2CoF-2 | No | 0.834 |
| OsI2 | -42m | - | 4.061 | - | - | - | 3.826 | 3.826 | P-4m2 | 1OsI2-2 | No | 0.842 |
| OsI2 | -3m | - | 3.601 | -1.032 | 1.177 | 2.209 | 4.056 | 4.056 | P-3m1 | 1OsI2-1 | Yes | 0.438 |
| OsI2 | -6m2 | - | 3.702 | - | - | - | 3.863 | 3.863 | P-6m2 | 1OsI2-4 | No | 1.067 |
| TaBr2 | -6m2 | - | 3.198 | - | - | - | 3.305 | 3.305 | P-6m2 | 1TaBr2-2 | No | 0.489 |
| TaBr2 | -42m | - | 4.11 | - | - | - | 3.262 | 3.262 | P-4m2 | 1TaBr2-3 | No | 0.673 |
| TaBr2 | -3m | - | 3.473 | - | - | - | 3.318 | 3.318 | P-3m1 | 1TaBr2-1 | No | 0.431 |
| WBr2 | -6m2 | - | 3.362 | - | - | - | 3.214 | 3.214 | P-6m2 | 1WBr2-3 | No | 0.928 |
| WBr2 | -42m | - | 3.921 | - | - | - | 3.203 | 3.203 | P-4m2 | 1WBr2-2 | No | 0.83 |
| WBr2 | -3m | - | 3.166 | - | - | - | 3.288 | 3.288 | P-3m1 | 1WBr2-1 | No | 0.737 |
| ZrCl2 | -3m | - | 4.292 | - | - | - | 3.335 | 3.335 | P-3m1 | 1ZrCl2-2 | No | 0.107 |
| ZrCl2 | -42m | - | 4.992 | - | - | - | 3.282 | 3.282 | P-4m2 | 1ZrCl2-3 | No | 0.467 |
| ZrCl2 | -6m2 | - | 3.519 | -0.152 | 1.349 | 1.5 | 3.41 | 3.41 | P-6m2 | 1ZrCl2-1 | Yes | 0 |
| Cr2I6 | -3m | 6 | 5.009 | -2.86 | -0.769 | 2.091 | 7.008 | 7.008 | P-31m | 2CrI3-1 | Yes | 0 |
| Rh2I6 | -3m | - | 4.869 | -2.667 | -0.702 | 1.965 | 6.934 | 6.934 | P-31m | 2RhI3-1 | Yes | 0 |
| Rh2I6 | -6m2 | - | 4.599 | -1.81 | -1.259 | 0.551 | 7.071 | 7.071 | P-62m | 2RhI3-2 | No | 0.342 |
| Ta2Cl6 | -6m2 | - | 4.605 | - | - | - | 5.769 | 5.769 | P-62m | 2TaCl3-2 | No | 0.167 |
| Ta2Cl6 | -3m | 1.863 | 4.683 | - | - | - | 5.774 | 5.774 | P-31m | 2TaCl3-1 | No | 0.142 |
| Zr2Br6 | -3m | 1.996 | 4.342 | - | - | - | 6.586 | 6.586 | P-31m | 2ZrBr3-1 | No | 0.073 |
| Zr2Br6 | -6m2 | 1.987 | 4.21 | - | - | - | 6.573 | 6.573 | P-62m | 2ZrBr3-2 | Yes | 0.104 |
| Ru2F8 | 2/m | 4 | 7.629 | -8.029 | -7.927 | 0.102 | 4.986 | 5.361 | P2_1/c | 2RuF4-1 | Yes | 0 |
| V2F8 | 2/m | 2 | 7.28 | -7.608 | -7.207 | 0.401 | 4.979 | 5.353 | P2_1/c | 2VF4-1 | Yes | 0 |
| Cd2Cl2 | 4/mmm | - | 5.617 | - | - | - | 4.357 | 4.357 | P4/nmm | 2CdCl-4 | No | 0.481 |
| Cd2Cl2 | -3m | - | 5.716 | - | - | 0 | 3.749 | 3.751 | P-3m1 | 2CdCl-2 | No | 0.167 |
| Cu2I2 | 4/mmm | - | 3.637 | -2.809 | 0.742 | 3.552 | 4.115 | 4.115 | P4/nmm | 2CuI-2 | No | 0.008 |
| Cu2I2 | -3m | - | 3.646 | -2.327 | 1.032 | 3.359 | 4.177 | 4.177 | P-3m1 | 2CuI-1 | Yes | 0 |
| NbF2 | -42m | - | 5.823 | - | - | - | 2.749 | 2.749 | P-4m2 | 1NbF2-2 | No | 0.552 |
| NiF2 | -42m | 2 | 6.102 | - | - | - | 3.434 | 3.434 | P-4m2 | 1NiF2-2 | No | 0.239 |
| RuF2 | -42m | - | 6.026 | -3.382 | -2.471 | 0.911 | 2.711 | 2.711 | P-4m2 | 1RuF2-1 | No | 0.57 |
| Ag2I6 | 6/mmm | - | 5.742 | - | - | - | 7.207 | 7.207 | P6/mmm | 2AgI3-4 | No | 0.209 |
| Ag2I6 | -3m | - | 5.898 | - | - | - | 7.057 | 7.057 | P-31m | 2AgI3-3 | No | 0.205 |
| Au2Br6 | 6/mmm | - | 6.373 | - | - | - | 7.188 | 7.188 | P6/mmm | 2AuBr3-3 | No | 0.225 |
| Au2Br6 | -3m | 3.717 | 6.086 | -4.129 | -3.269 | 0.86 | 6.847 | 6.847 | P-31m | 2AuBr3-2 | Yes | 0.121 |
| Ni2I6 | -3m | 2 | 5.388 | - | - | - | 6.837 | 6.837 | P-31m | 2NiI3-1 | Yes | 0.014 |
| Ni2I6 | -6m2 | 3.451 | 5.33 | - | - | - | 7.117 | 7.117 | P-62m | 2NiI3-3 | No | 0.186 |
| Pt2I6 | -3m | 1.948 | 4.793 | - | - | - | 7.133 | 7.133 | P-31m | 2PtI3-1 | No | 0.069 |
| Pt2I6 | -6m2 | - | 5.083 | - | - | - | 7.552 | 7.552 | P-62m | 2PtI3-2 | No | 0.292 |
| Au2Cl2 | 4/mmm | - | 6.246 | -3.708 | -2.931 | 0.777 | 4.01 | 4.01 | P4/nmm | 2AuCl-4 | No | 0.335 |
| VF2 | -42m | - | 6.444 | - | - | - | 2.589 | 2.589 | P-4m2 | 1VF2-2 | No | 0.515 |
| Mn2Br6 | -3m | 8 | 6.046 | - | - | - | 6.582 | 6.582 | P-31m | 2MnBr3-1 | No | 0 |
| W2Br6 | -6m2 | - | 4.022 | -0.633 | -0.162 | 0.471 | 5.914 | 5.914 | P-62m | 2WBr3-3 | Yes | 0.271 |

Table S2. All two-dimensional transition metal chalcogenides

| Formula | Point  group | Magnetic  moment | Work  fuction | vbm | cbm | band_gap | a_length | b_length | space_group | uid | Dynamically  stable | Energy  above  hull |
| --- | --- | --- | --- | --- | --- | --- | --- | --- | --- | --- | --- | --- |
| Fe4S8 | m | 3.759 | 5 | - | - | - | 5.415 | 6.564 | Pc | 4FeS2-1 | No | 0.363 |
| Zr2Te10 | mmm | - | 4.489 | -2.27 | -2.128 | 0.142 | 4.04 | 13.81 | Pmmn | 2ZrTe5-1 | Yes | 0 |
| Ag2Se2 | 2/m | - | 5.38 | -3.808 | -2.629 | 1.179 | 3.756 | 6.059 | P2/m | 2AgSe-1 | Yes | 0.089 |
| Ag2Se2 | -3m | - | 5.52 | - | - | 0 | 4.322 | 4.335 | P-3m1 | 2AgSe-4 | Yes | 0.22 |
| Co2Te2 | -3m | 0.864 | 4.633 | - | - | - | 3.967 | 3.967 | P-3m1 | 2CoTe-5 | No | 0.441 |
| Co2Te2 | 4/mmm | - | 4.086 | - | - | - | 3.744 | 3.744 | P4/nmm | 2CoTe-1 | Yes | 0.079 |
| Co2Te2 | -6m2 | 0.807 | 4.455 | - | - | - | 4.051 | 4.051 | P-6m2 | 2CoTe-4 | Yes | 0.419 |
| Co2Te2 | 4/mmm | - | 4.373 | - | - | - | 3.64 | 3.64 | P4/mmm | 2CoTe-3 | No | 0.158 |
| Co2Te2 | -3m | - | 4.1 | - | - | 0 | 3.946 | 3.947 | P-3m1 | 2CoTe-2 | Yes | 0.14 |
| Fe2Te2 | 4/mmm | 3.821 | 4.18 | - | - | - | 3.62 | 3.62 | P4/nmm | 2FeTe-1 | Yes | 0.082 |
| Fe2Te2 | -3m | 4.979 | 4.417 | - | - | - | 3.823 | 3.824 | P-3m1 | 2FeTe-2 | Yes | 0.134 |
| Fe2Te2 | -6m2 | 3.902 | 4.53 | - | - | - | 4.132 | 4.132 | P-6m2 | 2FeTe-4 | No | 0.515 |
| Fe2Te2 | -3m | 1.14 | 4.676 | - | - | - | 3.988 | 3.988 | P-3m1 | 2FeTe-5 | Yes | 0.517 |
| Fe2Te2 | 4/mmm | 4.932 | 4.274 | - | - | - | 3.659 | 3.662 | P4/mmm | 2FeTe-3 | No | 0.168 |
| Hf2Se2 | -3m | - | 3.596 | 0.238 | 0.357 | 0.119 | 4.266 | 4.267 | P-3m1 | 2HfSe-3 | No | 0.535 |
| Hf2Se2 | -3m | 0.678 | 4.632 | - | - | - | 3.626 | 3.626 | P-3m1 | 2HfSe-6 | No | 0.621 |
| Hf2Se2 | 4/mmm | - | 3.963 | - | - | - | 4.378 | 4.378 | P4/nmm | 2HfSe-4 | No | 0.572 |
| Hf2Se2 | 4/mmm | - | 4.674 | - | - | 0 | 4.175 | 4.179 | P4/mmm | 2HfSe-2 | No | 0.464 |
| Hf2Se2 | -6m2 | 0.419 | 4.601 | - | - | - | 3.617 | 3.617 | P-6m2 | 2HfSe-5 | No | 0.614 |
| Hg2Te2 | -3m | - | 5.209 | -2.16 | -1.872 | 0.288 | 4.723 | 4.723 | P-3m1 | 2HgTe-2 | No | 0.158 |
| Hg2Te2 | -3m | - | 5.084 | -1.961 | -1.834 | 0.127 | 4.722 | 4.724 | P-3m1 | 2HgTe-1 | No | 0.108 |
| Ir2Se2 | -6m2 | - | 4.178 | 0.419 | 1.241 | 0.822 | 3.367 | 3.367 | P-6m2 | 2IrSe-3 | No | 0.426 |
| Ir2Se2 | -3m | - | 4.381 | 0.834 | 0.997 | 0.163 | 3.408 | 3.417 | P-3m1 | 2IrSe-1 | Yes | 0.244 |
| Ir2Se2 | 4/mmm | - | 3.744 | - | - | - | 3.952 | 3.952 | P4/nmm | 2IrSe-2 | No | 0.317 |
| Ir2Se2 | m | - | 4.503 | - | - | - | 2.829 | 4.701 | Pm | 2IrSe-4 | No | 0.432 |
| Ir2Se2 | -3m | - | 4.074 | 0.641 | 0.742 | 0.101 | 3.386 | 3.386 | P-3m1 | 2IrSe-5 | No | 0.454 |
| Mo2Te2 | 4/mmm | - | 3.74 | - | - | - | 3.895 | 3.895 | P4/nmm | 2MoTe-1 | No | 0.224 |
| Mo2Te2 | -6m2 | - | 4.309 | - | - | - | 3.268 | 3.268 | P-6m2 | 2MoTe-5 | No | 0.666 |
| Mo2Te2 | -3m | - | 3.973 | - | - | 0 | 4.06 | 4.06 | P-3m1 | 2MoTe-2 | No | 0.323 |
| Mo2Te2 | mm2 | - | 4.182 | - | - | - | 3.782 | 3.905 | Pmm2 | 2MoTe-3 | No | 0.34 |
| Nb2Te2 | -3m | - | 4.135 | - | - | 0 | 4.21 | 4.21 | P-3m1 | 2NbTe-3 | Yes | 0.276 |
| Nb2Te2 | -6m2 | 1.14 | 4.539 | - | - | - | 3.42 | 3.42 | P-6m2 | 2NbTe-4 | No | 0.636 |
| Nb2Te2 | 4/mmm | - | 4.574 | - | - | 0 | 3.954 | 3.955 | P4/mmm | 2NbTe-2 | No | 0.233 |
| Nb2Te2 | -3m | - | 4.246 | - | - | - | 3.494 | 3.494 | P-3m1 | 2NbTe-5 | No | 0.641 |
| Ni2Se2 | 4/mmm | - | 4.521 | - | - | - | 3.893 | 3.893 | P4/nmm | 2NiSe-3 | Yes | 0.087 |
| Ni2Se2 | -3m | - | 4.734 | - | - | 0 | 3.742 | 3.743 | P-3m1 | 2NiSe-1 | Yes | 0.022 |
| Ni2Se2 | -3m | - | 5.264 | - | - | - | 3.763 | 3.763 | P-3m1 | 2NiSe-5 | No | 0.324 |
| Ni2Se2 | 4/mmm | 1.116 | 4.666 | - | - | - | 3.624 | 3.624 | P4/mmm | 2NiSe-2 | Yes | 0.04 |
| Ni2Se2 | -6m2 | - | 4.99 | - | - | - | 3.873 | 3.873 | P-6m2 | 2NiSe-4 | Yes | 0.28 |
| Pd2S2 | -3m | - | 5.244 | - | - | 0 | 3.867 | 3.87 | P-3m1 | 2PdS-4 | Yes | 0.302 |
| Pd2S2 | -3m | - | 5.571 | - | - | - | 3.719 | 3.719 | P-3m1 | 2PdS-7 | No | 0.448 |
| Pd2S2 | -6m2 | - | 5.601 | - | - | - | 3.741 | 3.741 | P-6m2 | 2PdS-6 | No | 0.446 |
| Pt2Se2 | -6m2 | - | 4.838 | - | - | - | 3.923 | 3.923 | P-6m2 | 2PtSe-3 | No | 0.426 |
| Pt2Se2 | 4/mmm | - | 4.416 | -1.013 | -0.969 | 0.044 | 3.943 | 3.943 | P4/nmm | 2PtSe-5 | No | 0.483 |
| Pt2Se2 | -3m | - | 4.595 | - | - | 0 | 3.73 | 3.732 | P-3m1 | 2PtSe-2 | No | 0.265 |
| Pt2Se2 | 4/mmm | - | 4.378 | - | - | 0 | 3.967 | 3.968 | P4/mmm | 2PtSe-1 | Yes | 0.194 |
| Pt2Se2 | -3m | - | 4.823 | - | - | - | 3.946 | 3.946 | P-3m1 | 2PtSe-4 | No | 0.432 |
| Re2Se2 | m | - | 4.175 | - | - | - | 3.014 | 4.157 | Pm | 2ReSe-4 | No | 0.609 |
| Re2Se2 | -3m | - | 3.462 | - | - | - | 3.93 | 3.93 | P-3m1 | 2ReSe-1 | Yes | 0.512 |
| Re2Se2 | 4/mmm | - | 3.778 | - | - | - | 3.852 | 3.852 | P4/nmm | 2ReSe-2 | Yes | 0.546 |
| Re2Se2 | -6m2 | - | 4.239 | - | - | - | 3.004 | 3.004 | P-6m2 | 2ReSe-3 | No | 0.597 |
| Rh2Se2 | 4/mmm | - | 4.004 | - | - | - | 3.96 | 3.96 | P4/nmm | 2RhSe-1 | No | 0.022 |
| Rh2Se2 | -6m2 | - | 4.337 | 0.28 | 0.618 | 0.337 | 3.342 | 3.342 | P-6m2 | 2RhSe-3 | No | 0.164 |
| Rh2Se2 | -3m | - | 4.294 | - | - | - | 3.417 | 3.417 | P-3m1 | 2RhSe-4 | No | 0.191 |
| Rh2Se2 | -3m | - | 4.486 | - | - | 0 | 3.996 | 3.997 | P-3m1 | 2RhSe-2 | Yes | 0.05 |
| Rh2Se2 | 2/m | - | 5.038 | - | - | - | 2.835 | 4.714 | P2/m | 2RhSe-5 | No | 0.255 |
| Ru2Se2 | 4/mmm | - | 4.291 | - | - | - | 3.978 | 3.978 | P4/nmm | 2RuSe-1 | Yes | 0.087 |
| Ru2Se2 | -3m | - | 4.151 | - | - | 0 | 3.962 | 3.963 | P-3m1 | 2RuSe-2 | No | 0.341 |
| Ru2Se2 | -6m2 | - | 4.334 | - | - | - | 3.7 | 3.7 | P-6m2 | 2RuSe-5 | No | 0.465 |
| Ru2Se2 | -3m | - | 4.508 | - | - | - | 3.699 | 3.699 | P-3m1 | 2RuSe-3 | No | 0.438 |
| Ru2Se2 | m | - | 4.391 | - | - | - | 2.932 | 4.28 | Pm | 2RuSe-4 | No | 0.455 |
| Sc2Te2 | -3m | - | 4.654 | -0.175 | -0.173 | 0.002 | 4.053 | 4.053 | P-3m1 | 2ScTe-5 | No | 0.627 |
| Sc2Te2 | 4/mmm | - | 4.827 | - | - | 0 | 4.457 | 4.458 | P4/mmm | 2ScTe-3 | No | 0.459 |
| Sc2Te2 | 4/mmm | - | 3.883 | - | - | - | 4.862 | 4.862 | P4/nmm | 2ScTe-1 | No | 0.315 |
| Sc2Te2 | -6m2 | - | 4.637 | -0.556 | 0.162 | 0.718 | 4.079 | 4.079 | P-6m2 | 2ScTe-4 | No | 0.614 |
| Sc2Te2 | -3m | 1.116 | 3.875 | - | - | - | 4.646 | 4.65 | P-3m1 | 2ScTe-2 | No | 0.324 |
| Ta2Te2 | 4/mmm | - | 4.957 | - | - | - | 3.941 | 3.941 | P4/nmm | 2TaTe-1 | No | 0.185 |
| Ta2Te2 | 4/mmm | - | 4.35 | - | - | 0 | 3.971 | 3.968 | P4/mmm | 2TaTe-2 | No | 0.239 |
| Ta2Te2 | -6m2 | 0.919 | 4.337 | - | - | - | 3.263 | 3.263 | P-6m2 | 2TaTe-4 | No | 0.687 |
| Ta2Te2 | -3m | - | 3.974 | - | - | 0 | 4.195 | 4.196 | P-3m1 | 2TaTe-3 | Yes | 0.355 |
| Ag2S4 | 2/m | - | 4.838 | - | - | - | 5.883 | 6.039 | P2_1/c | 2AgS2-1 | No | 0.104 |
| Au2S4 | 2/m | - | 4.696 | - | - | - | 5.621 | 6.282 | P2_1/c | 2AuS2-1 | Yes | 0.133 |
| Co4S8 | -1 | - | 5.771 | -2.816 | -2.198 | 0.618 | 6.02 | 6.615 | P-1 | 4CoS2-1 | Yes | 0.071 |
| CrTe2 | -42m | 2 | 4.869 | - | - | - | 4.133 | 4.133 | P-4m2 | 1CrTe2-3 | Yes | 0.369 |
| CrTe2 | -6m2 | - | 4.548 | -0.105 | 0.764 | 0.869 | 3.47 | 3.47 | P-6m2 | 1CrTe2-2 | Yes | 0.108 |
| Cr2Te4 | 4/mmm | 5.689 | 4.751 | - | - | - | 6.81 | 6.81 | P4/mbm | 2CrTe2-3 | No | 0.408 |
| CrTe2 | -3m | 2.43 | 5.134 | - | - | - | 3.689 | 3.689 | P-3m1 | 1CrTe2-1 | No | 0.024 |
| CuTe2 | -3m | - | 4.904 | - | - | - | 3.499 | 3.499 | P-3m1 | 1CuTe2-3 | No | 0.213 |
| CuTe2 | -42m | - | 4.752 | - | - | - | 3.199 | 3.199 | P-4m2 | 1CuTe2-1 | No | 0.072 |
| CuTe2 | -6m2 | - | 4.018 | - | - | - | 3.916 | 3.916 | P-6m2 | 1CuTe2-2 | Yes | 0.195 |
| Cu2Te4 | 2/m | - | 4.826 | - | - | - | 3.147 | 6.565 | C2/m | 2CuTe2-2 | No | 0.194 |
| Cu2Te4 | 2/m | - | 4.594 | - | - | - | 6.3 | 6.574 | P2_1/c | 2CuTe2-1 | No | 0.158 |
| FeTe2 | -42m | 3.152 | 4.515 | - | - | - | 3.279 | 3.279 | P-4m2 | 1FeTe2-3 | No | 0.392 |
| FeTe2 | -6m2 | 1.744 | 4.268 | - | - | - | 3.569 | 3.569 | P-6m2 | 1FeTe2-2 | Yes | 0.168 |
| Fe2Te4 | 2/m | - | 4.397 | -0.533 | 0.048 | 0.581 | 3.649 | 5.938 | P2_1/m | 2FeTe2-1 | Yes | 0.092 |
| Fe2Te4 | 4/mmm | 5.86 | 4.86 | - | - | - | 6.669 | 6.669 | P4/mbm | 2FeTe2-2 | No | 0.467 |
| FeTe2 | -3m | 1.522 | 4.613 | - | - | - | 3.622 | 3.622 | P-3m1 | 1FeTe2-1 | No | 0.146 |
| HfTe2 | -6m2 | - | 5.154 | -1.305 | -0.64 | 0.665 | 3.909 | 3.909 | P-6m2 | 1HfTe2-2 | Yes | 0.133 |
| HfTe2 | -3m | - | 4.67 | - | - | - | 3.977 | 3.977 | P-3m1 | 1HfTe2-1 | Yes | 0.007 |
| HfTe2 | -42m | - | 4.834 | -2.82 | -1.076 | 1.744 | 4.282 | 4.282 | P-4m2 | 1HfTe2-3 | Yes | 0.371 |
| Ir2S4 | 2/m | - | 5.37 | - | - | 0 | 5.665 | 5.671 | P2_1/c | 2IrS2-2 | No | 0.408 |
| Ir2S4 | 2/m | - | 5.603 | - | - | - | 3.57 | 5.908 | P2_1/m | 2IrS2-1 | No | 0.15 |
| IrTe2 | -3m | - | 4.749 | - | - | - | 3.881 | 3.881 | P-3m1 | 1IrTe2-1 | No | 0.137 |
| IrTe2 | -42m | - | 4.044 | - | - | - | 3.411 | 3.411 | P-4m2 | 1IrTe2-3 | No | 0.495 |
| IrTe2 | -6m2 | - | 4.084 | - | - | - | 3.795 | 3.795 | P-6m2 | 1IrTe2-2 | No | 0.304 |
| Ir5Te10 | 2 | - | 4.601 | - | - | - | 4.01 | 16.325 | C2 | 5IrTe2-1 | Yes | 0.077 |
| Ir2Te4 | 2/m | - | 4.678 | - | - | - | 3.908 | 6.622 | P2_1/m | 2IrTe2-1 | No | 0.13 |
| Ir2Te4 | 2/m | - | 5.009 | - | - | - | 6.427 | 6.54 | P2_1/c | 2IrTe2-2 | No | 0.536 |
| Ir4Te8 | -1 | - | 4.45 | -1.045 | -0.147 | 0.898 | 7.083 | 7.93 | P-1 | 4IrTe2-1 | Yes | 0.066 |
| MnS2 | -3m | 2.964 | 5.722 | -2.327 | -1.193 | 1.134 | 3.35 | 3.35 | P-3m1 | 1MnS2-1 | Yes | 0 |
| MnS2 | -42m | 0.838 | 5.326 | - | - | - | 3.475 | 3.475 | P-4m2 | 1MnS2-3 | Yes | 0.333 |
| MnS2 | -6m2 | 1 | 5.242 | - | - | - | 3.091 | 3.091 | P-6m2 | 1MnS2-2 | No | 0.115 |
| MoTe2 | -3m | - | 4.202 | - | - | - | 3.493 | 3.493 | P-3m1 | 1MoTe2-2 | No | 0.171 |
| MoTe2 | -6m2 | - | 4.3 | -0.053 | 1.317 | 1.37 | 3.547 | 3.547 | P-6m2 | 1MoTe2-1 | Yes | 0 |
| MoTe2 | -42m | 0.419 | 4.418 | - | - | - | 4.029 | 4.029 | P-4m2 | 1MoTe2-3 | No | 0.64 |
| Mo2Te4 | 4/mmm | 4.093 | 4.594 | - | - | - | 6.954 | 6.953 | P4/mbm | 2MoTe2-2 | Yes | 0.819 |
| NbSe2 | -3m | - | 4.902 | - | - | - | 3.481 | 3.481 | P-3m1 | 1NbSe2-2 | Yes | 0.033 |
| NbSe2 | -6m2 | - | 5.538 | - | - | - | 3.472 | 3.472 | P-6m2 | 1NbSe2-1 | Yes | 0.001 |
| NbSe2 | -42m | 0.91 | 4.704 | - | - | - | 3.854 | 3.854 | P-4m2 | 1NbSe2-3 | No | 0.438 |
| Ni2Te4 | 2/m | - | 4.483 | -3.124 | -1.421 | 1.703 | 5.933 | 6.241 | P2_1/c | 2NiTe2-1 | Yes | 0.176 |
| Os2Te4 | 2/m | - | 4.074 | -0.461 | 0.918 | 1.379 | 3.876 | 6.133 | P2_1/m | 2OsTe2-1 | Yes | 0.071 |
| Os2Te4 | 4/mmm | - | 4.961 | -2.503 | -2.312 | 0.192 | 6.651 | 6.651 | P4/mbm | 2OsTe2-2 | No | 0.77 |
| PdS2 | -3m | - | 5.706 | -3.226 | -1.545 | 1.681 | 3.553 | 3.553 | P-3m1 | 1PdS2-2 | Yes | 0.101 |
| PdS2 | -6m2 | - | 4.084 | -1.613 | -1.503 | 0.11 | 3.915 | 3.915 | P-6m2 | 1PdS2-3 | No | 0.35 |
| PdS2 | -42m | - | 5.648 | - | - | - | 3.193 | 3.193 | P-4m2 | 1PdS2-4 | No | 0.632 |
| Pd2S4 | 2/m | - | 4.987 | -3.716 | -1.701 | 2.015 | 5.483 | 5.583 | P2_1/c | 2PdS2-1 | Yes | 0.002 |
| PtS2 | -42m | - | 5.412 | - | - | - | 3.274 | 3.274 | P-4m2 | 1PtS2-2 | No | 0.591 |
| Pt2S4 | 2/m | - | 4.692 | -3.557 | -0.77 | 2.787 | 5.472 | 5.56 | P2_1/c | 2PtS2-1 | Yes | 0.05 |
| PtS2 | -6m2 | - | 5.115 | - | - | - | 3.491 | 3.491 | P-6m2 | 1PtS2-3 | No | 0.593 |
| PtS2 | -3m | - | 5.402 | -3.218 | -0.73 | 2.488 | 3.574 | 3.574 | P-3m1 | 1PtS2-1 | Yes | 0 |
| ReTe2 | -42m | - | 4.207 | - | - | - | 4.097 | 4.097 | P-4m2 | 1ReTe2-3 | No | 0.684 |
| ReTe2 | -3m | - | 4.041 | - | - | - | 3.395 | 3.395 | P-3m1 | 1ReTe2-2 | No | 0.4 |
| ReTe2 | -6m2 | - | 3.918 | - | - | - | 3.701 | 3.701 | P-6m2 | 1ReTe2-1 | No | 0.273 |
| Re2Te4 | 2/m | - | 3.877 | - | - | - | 3.458 | 6.311 | P2_1/m | 2ReTe2-1 | No | 0.121 |
| Re4Te8 | -1 | - | 4.103 | -0.129 | 1.202 | 1.331 | 7.067 | 7.204 | P-1 | 4ReTe2-1 | Yes | 0 |
| RhSe2 | -3m | - | 5.451 | - | - | - | 3.596 | 3.596 | P-3m1 | 1RhSe2-1 | No | 0.117 |
| RhSe2 | -6m2 | - | 4.665 | - | - | - | 3.558 | 3.558 | P-6m2 | 1RhSe2-2 | No | 0.283 |
| Rh2Se4 | 2/m | - | 5.277 | - | - | - | 3.639 | 6.049 | P2_1/m | 2RhSe2-1 | No | 0.091 |
| RhSe2 | -42m | - | 4.652 | - | - | - | 3.155 | 3.155 | P-4m2 | 1RhSe2-3 | No | 0.398 |
| RuSe2 | -42m | - | 4.786 | - | - | - | 3.84 | 3.84 | P-4m2 | 1RuSe2-3 | Yes | 0.473 |
| RuSe2 | -6m2 | - | 4.392 | - | - | - | 3.471 | 3.471 | P-6m2 | 1RuSe2-2 | No | 0.31 |
| RuSe2 | -3m | 1.571 | 5.111 | - | - | - | 3.476 | 3.476 | P-3m1 | 1RuSe2-1 | No | 0.309 |
| Ru2Se4 | 4/mmm | - | 5.304 | -3.007 | -2.848 | 0.159 | 6.134 | 6.133 | P4/mbm | 2RuSe2-2 | No | 0.611 |
| TaS2 | -3m | - | 5.083 | - | - | - | 3.381 | 3.381 | P-3m1 | 1TaS2-2 | Yes | 0.02 |
| TaS2 | -6m2 | - | 5.932 | - | - | - | 3.341 | 3.341 | P-6m2 | 1TaS2-1 | Yes | 0 |
| TaS2 | -42m | - | 4.614 | - | - | - | 3.69 | 3.69 | P-4m2 | 1TaS2-3 | Yes | 0.448 |
| V2Se4 | 4/mmm | 3.796 | 5.023 | - | - | - | 6.328 | 6.328 | P4/mbm | 2VSe2-1 | No | 0.607 |
| W2S4 | 2/m | - | 5.661 | - | - | - | 3.201 | 5.725 | P2_1/m | 2WS2-1 | Yes | 0.177 |
| W2S4 | 4/mmm | 3.779 | 4.607 | - | - | - | 6.088 | 6.088 | P4/mbm | 2WS2-2 | Unknown | 1.188 |
| WTe2 | -42m | - | 4.189 | - | - | - | 3.908 | 3.908 | P-4m2 | 1WTe2-3 | No | 0.718 |
| WTe2 | -3m | - | 3.933 | - | - | - | 3.507 | 3.507 | P-3m1 | 1WTe2-2 | No | 0.215 |
| W2Te4 | 4/mmm | 4.018 | 4.421 | - | - | - | 6.956 | 6.957 | P4/mbm | 2WTe2-2 | No | 0.972 |
| W2Te4 | 2/m | - | 4.38 | 0.288 | 0.333 | 0.044 | 3.492 | 6.314 | P2_1/m | 2WTe2-1 | Yes | 0 |
| WTe2 | -6m2 | - | 4.055 | 0.332 | 1.473 | 1.141 | 3.55 | 3.55 | P-6m2 | 1WTe2-1 | Yes | 0.026 |
| YSe2 | -42m | - | 6.654 | - | - | - | 4.083 | 4.083 | P-4m2 | 1YSe2-1 | No | 0.489 |
| ZnSe2 | -42m | - | 5.106 | - | - | - | 3.142 | 3.142 | P-4m2 | 1ZnSe2-1 | No | 0.355 |
| Zn2Se4 | 2/m | - | 4.323 | -3.197 | -0.983 | 2.214 | 5.977 | 5.757 | P2_1/c | 2ZnSe2-1 | No | 0.316 |
| ZrSe2 | -42m | - | 5.325 | -3.792 | -1.285 | 2.507 | 4.038 | 4.038 | P-4m2 | 1ZrSe2-3 | Yes | 0.319 |
| ZrSe2 | -3m | - | 5.289 | -2.067 | -0.864 | 1.202 | 3.795 | 3.795 | P-3m1 | 1ZrSe2-1 | Yes | 0 |
| ZrSe2 | -6m2 | - | 5.886 | -2.771 | -1.087 | 1.684 | 3.702 | 3.702 | P-6m2 | 1ZrSe2-2 | No | 0.144 |
| Ni2S6 | 2/m | - | 5.537 | - | - | - | 4.596 | 3.359 | P2_1/m | 2NiS3-1 | Yes | 0.134 |
| Pt2Se6 | 2/m | - | 4.838 | - | - | - | 6.103 | 3.716 | P2_1/m | 2PtSe3-1 | Yes | 0.18 |
| Rh2Te6 | 2/m | - | 4.616 | - | - | - | 6.112 | 3.743 | P2_1/m | 2RhTe3-2 | Yes | 0.216 |
| HgTe | 3m | - | 5.03 | -3.27 | -2.991 | 0.279 | 4.732 | 4.732 | P3m1 | 1HgTe-1 | Yes | 0.165 |
| Os2S2 | m | - | 4.857 | - | - | - | 2.837 | 4.491 | Pm | 2OsS-5 | No | 0.659 |
| Os2S2 | -3m | - | 4.468 | - | - | 0 | 3.051 | 3.05 | P-3m1 | 2OsS-1 | No | 0.346 |
| Os2S2 | 4/mmm | - | 4.336 | - | - | - | 3.902 | 3.902 | P4/nmm | 2OsS-2 | Yes | 0.363 |
| Os2S2 | -3m | - | 4.423 | - | - | - | 3.027 | 3.027 | P-3m1 | 2OsS-3 | No | 0.447 |
| Os2S2 | -6m2 | - | 4.226 | - | - | - | 3.615 | 3.615 | P-6m2 | 2OsS-4 | No | 0.546 |
| Rh2Te2 | 4/mmm | - | 3.802 | - | - | - | 4.05 | 4.05 | P4/nmm | 2RhTe-1 | Yes | 0.059 |
| Rh2Te2 | 4/mmm | - | 4.57 | - | - | 0 | 3.923 | 3.923 | P4/mmm | 2RhTe-3 | Yes | 0.13 |
| Rh2Te2 | -3m | - | 4.004 | - | - | - | 3.66 | 3.66 | P-3m1 | 2RhTe-5 | No | 0.343 |
| Rh2Te2 | -6m2 | - | 3.919 | - | - | - | 3.642 | 3.642 | P-6m2 | 2RhTe-4 | No | 0.338 |
| Rh2Te2 | -3m | - | 4.2 | - | - | 0 | 4.184 | 4.185 | P-3m1 | 2RhTe-2 | Yes | 0.09 |
| Ru2Te2 | 4/mmm | - | 4.056 | - | - | - | 4.115 | 4.115 | P4/nmm | 2RuTe-1 | Yes | 0.161 |
| Ru2Te2 | -3m | - | 3.897 | - | - | 0 | 4.128 | 4.129 | P-3m1 | 2RuTe-2 | Yes | 0.386 |
| Ru2Te2 | -6m2 | - | 4.342 | - | - | - | 4.1 | 4.1 | P-6m2 | 2RuTe-4 | No | 0.59 |
| Ru2Te2 | mm2 | - | 4.1 | - | - | - | 2.959 | 3.799 | Pmm2 | 2RuTe-3 | No | 0.538 |
| AgS2 | -42m | - | 5.744 | - | - | - | 2.713 | 2.713 | P-4m2 | 1AgS2-2 | No | 0.467 |
| AuS2 | -42m | - | 5.949 | - | - | - | 3.384 | 3.384 | P-4m2 | 1AuS2-3 | No | 0.727 |
| AuS2 | -3m | - | 5.447 | - | - | - | 3.656 | 3.656 | P-3m1 | 1AuS2-1 | No | 0.4 |
| AuS2 | -6m2 | - | 5.611 | - | - | - | 3.593 | 3.593 | P-6m2 | 1AuS2-2 | No | 0.665 |
| CoS2 | -6m2 | - | 5.166 | - | - | - | 3.226 | 3.226 | P-6m2 | 1CoS2-2 | No | 0.237 |
| CoS2 | -42m | - | 5.383 | - | - | - | 2.929 | 2.929 | P-4m2 | 1CoS2-3 | No | 0.402 |
| CoS2 | -3m | - | 5.924 | - | - | - | 3.219 | 3.219 | P-3m1 | 1CoS2-1 | No | 0.11 |
| Co2S4 | 2/m | 1.546 | 5.172 | - | - | - | 5.404 | 5.432 | P2_1/c | 2CoS2-3 | Yes | 0.237 |
| Co2S4 | 2/m | - | 5.748 | - | - | - | 3.242 | 5.502 | P2_1/m | 2CoS2-1 | No | 0.093 |
| Cr2S4 | 4/mmm | 4.965 | 4.792 | - | - | - | 5.809 | 5.809 | P4/mbm | 2CrS2-2 | Yes | 0.48 |
| HgS2 | -6m2 | - | 5.213 | - | - | - | 4.312 | 4.312 | P-6m2 | 1HgS2-1 | No | 0.574 |
| HgS2 | -3m | 1.812 | 6.095 | - | - | - | 3.873 | 3.873 | P-3m1 | 1HgS2-2 | Unknown | 0.673 |
| HgS2 | -42m | - | 5.841 | - | - | - | 3.09 | 3.09 | P-4m2 | 1HgS2-3 | No | 0.689 |
| Mn2Se4 | 2/m | 1.959 | 4.996 | - | - | - | 3.302 | 5.642 | P2_1/m | 2MnSe2-1 | Yes | 0.053 |
| Mo2S4 | 2/m | - | 5.78 | -1.552 | -1.482 | 0.069 | 3.183 | 5.724 | P2_1/m | 2MoS2-1 | Yes | 0.18 |
| Mo2S4 | 4/mmm | 3.993 | 4.657 | -2.337 | -2.331 | 0.006 | 6.042 | 6.042 | P4/mbm | 2MoS2-2 | Yes | 1.022 |
| NiTe2 | -3m | - | 4.431 | - | - | - | 3.783 | 3.783 | P-3m1 | 1NiTe2-1 | Yes | 0.067 |
| NiTe2 | -42m | 0.589 | 4.59 | - | - | - | 3.239 | 3.239 | P-4m2 | 1NiTe2-3 | No | 0.301 |
| NiTe2 | -6m2 | - | 3.968 | - | - | - | 3.715 | 3.715 | P-6m2 | 1NiTe2-2 | Yes | 0.164 |
| Pt2Se4 | 2/m | - | 4.67 | -3.273 | -0.976 | 2.297 | 5.735 | 5.905 | P2_1/c | 2PtSe2-1 | Yes | 0.103 |
| VSe2 | -6m2 | 0.992 | 5.434 | -1.328 | -0.35 | 0.977 | 3.343 | 3.343 | P-6m2 | 1VSe2-1 | Yes | 0 |
| VSe2 | -42m | 1 | 5.346 | - | - | - | 3.753 | 3.753 | P-4m2 | 1VSe2-3 | Yes | 0.275 |
| VSe2 | -3m | 0.624 | 5 | - | - | - | 3.347 | 3.347 | P-3m1 | 1VSe2-2 | Yes | 0.016 |
| WS2 | -42m | - | 4.77 | - | - | - | 3.52 | 3.52 | P-4m2 | 1WS2-3 | No | 0.715 |
| WS2 | -3m | - | 4.868 | - | - | - | 3.21 | 3.21 | P-3m1 | 1WS2-2 | No | 0.296 |
| WS2 | -6m2 | - | 4.733 | -1.023 | 1.032 | 2.055 | 3.186 | 3.186 | P-6m2 | 1WS2-1 | Yes | 0 |
| YTe2 | -42m | - | 5.71 | - | - | - | 4.2 | 4.2 | P-4m2 | 1YTe2-1 | No | 0.56 |
| Zn2Te4 | 2/m | - | 4.185 | -2.596 | -0.959 | 1.637 | 6.701 | 6.185 | P2_1/c | 2ZnTe2-1 | No | 0.317 |
| Ni2Se6 | 2/m | - | 5.377 | - | - | - | 5.076 | 3.405 | P2_1/m | 2NiSe3-1 | No | 0.097 |
| Pt2Te6 | 2/m | - | 4.502 | - | - | - | 6.658 | 3.916 | P2_1/m | 2PtTe3-1 | Yes | 0.176 |
| Ag2Te2 | 2/m | - | 4.773 | -2.863 | -2.108 | 0.756 | 4.083 | 6.31 | P2/m | 2AgTe-3 | Yes | 0.11 |
| Ag2Te2 | -3m | - | 5.274 | - | - | 0 | 4.202 | 4.207 | P-3m1 | 2AgTe-4 | Yes | 0.136 |
| Ag2Te2 | -3m | - | 5.706 | - | - | - | 4.331 | 4.331 | P-3m1 | 2AgTe-6 | No | 0.343 |
| Cu2S2 | -3m | - | 5.612 | - | - | 0 | 3.803 | 3.805 | P-3m1 | 2CuS-1 | Yes | 0.111 |
| Cu2S2 | -6m2 | - | 4.324 | - | - | - | 3.858 | 3.858 | P-6m2 | 2CuS-4 | No | 0.209 |
| Cu2S2 | 2/m | - | 5.584 | -4.397 | -3.086 | 1.311 | 3.35 | 5.415 | P2/m | 2CuS-2 | Yes | 0.127 |
| Ni2Te2 | 4/mmm | 0.489 | 4.63 | - | - | - | 3.651 | 3.651 | P4/nmm | 2NiTe-2 | Yes | 0.063 |
| Ni2Te2 | -3m | - | 4.889 | - | - | - | 4.068 | 4.068 | P-3m1 | 2NiTe-5 | Yes | 0.368 |
| Ni2Te2 | 4/mmm | 1.034 | 4.27 | - | - | - | 3.736 | 3.736 | P4/mmm | 2NiTe-3 | Yes | 0.068 |
| Ni2Te2 | -6m2 | - | 4.453 | - | - | - | 4.166 | 4.166 | P-6m2 | 2NiTe-4 | Yes | 0.299 |
| Ni2Te2 | -3m | - | 4.416 | - | - | 0 | 3.899 | 3.9 | P-3m1 | 2NiTe-1 | Yes | 0.036 |
| Re2Te2 | mm2 | - | 4.154 | - | - | - | 3.021 | 3.74 | Pmm2 | 2ReTe-3 | No | 0.527 |
| Re2Te2 | -3m | - | 3.972 | - | - | - | 3.937 | 3.937 | P-3m1 | 2ReTe-1 | No | 0.423 |
| Re2Te2 | -6m2 | - | 3.765 | - | - | - | 3.143 | 3.143 | P-6m2 | 2ReTe-4 | No | 0.784 |
| Re2Te2 | 4/mmm | - | 3.443 | - | - | - | 3.948 | 3.948 | P4/nmm | 2ReTe-2 | No | 0.501 |
| Ag2Se4 | 2/m | - | 4.728 | - | - | - | 6.149 | 6.375 | P2_1/c | 2AgSe2-1 | No | 0.093 |
| Ir4S8 | -1 | - | 5.342 | -2.468 | -0.948 | 1.52 | 6.444 | 7.117 | P-1 | 4IrS2-1 | Yes | 0.076 |
| MnSe2 | -6m2 | 1.011 | 4.885 | - | - | - | 3.254 | 3.254 | P-6m2 | 1MnSe2-2 | No | 0.143 |
| MnSe2 | -42m | 3 | 5.26 | - | - | - | 3.823 | 3.823 | P-4m2 | 1MnSe2-3 | No | 0.376 |
| MnSe2 | -3m | 2.827 | 5.097 | - | - | - | 3.494 | 3.494 | P-3m1 | 1MnSe2-1 | Yes | 0 |
| OsTe2 | -6m2 | - | 3.955 | - | - | - | 3.741 | 3.741 | P-6m2 | 1OsTe2-1 | No | 0.313 |
| OsTe2 | -3m | - | 4.52 | - | - | - | 3.86 | 3.86 | P-3m1 | 1OsTe2-2 | No | 0.319 |
| OsTe2 | -42m | - | 4.082 | - | - | - | 3.296 | 3.296 | P-4m2 | 1OsTe2-3 | No | 0.523 |
| PtSe2 | -42m | - | 4.821 | - | - | - | 3.375 | 3.375 | P-4m2 | 1PtSe2-3 | No | 0.546 |
| PtSe2 | -6m2 | - | 4.616 | - | - | - | 3.646 | 3.646 | P-6m2 | 1PtSe2-2 | Yes | 0.452 |
| PtSe2 | -3m | - | 4.883 | -2.169 | -0.533 | 1.636 | 3.748 | 3.748 | P-3m1 | 1PtSe2-1 | Yes | 0 |
| Rh2Te4 | 2/m | - | 5.08 | - | - | - | 6.451 | 6.559 | P2_1/c | 2RhTe2-2 | No | 0.425 |
| Rh2Te4 | 2/m | - | 4.816 | - | - | - | 3.837 | 6.459 | P2_1/m | 2RhTe2-1 | No | 0.106 |
| ZnTe2 | -42m | - | 4.54 | - | - | - | 3.453 | 3.453 | P-4m2 | 1ZnTe2-1 | Yes | 0.237 |
| Ni2Te6 | 2/m | - | 4.515 | - | - | - | 6.594 | 3.711 | P2_1/m | 2NiTe3-3 | Yes | 0.16 |
| Ta2S6 | mmm | - | 4.925 | - | - | - | 4.982 | 3.364 | Pmmn | 2TaS3-1 | Yes | 0.044 |
| Cr2Se2 | 4/mmm | 0.711 | 4.441 | - | - | - | 3.52 | 3.52 | P4/nmm | 2CrSe-1 | Yes | 0.143 |
| V3Te6 | 2/m | 1.169 | 4.728 | - | - | - | 3.537 | 9.523 | C2/m | 3VTe2-1 | No | 0.015 |
| Hf2Te2 | -3m | - | 4.531 | - | - | - | 3.713 | 3.713 | P-3m1 | 2HfTe-5 | No | 0.639 |
| Hf2Te2 | -3m | 1.441 | 3.84 | - | - | - | 4.417 | 4.419 | P-3m1 | 2HfTe-3 | No | 0.429 |
| Hf2Te2 | 4/mmm | - | 4.377 | - | - | 0 | 4.271 | 4.275 | P4/mmm | 2HfTe-1 | No | 0.286 |
| Hf2Te2 | -6m2 | - | 4.485 | 0.69 | 0.834 | 0.145 | 3.727 | 3.727 | P-6m2 | 2HfTe-4 | No | 0.625 |
| Hf2Te2 | 4/mmm | - | 4.079 | - | - | - | 4.428 | 4.428 | P4/nmm | 2HfTe-2 | No | 0.415 |
| Mo2S2 | mm2 | - | 5.012 | - | - | - | 3.675 | 3.805 | Pmm2 | 2MoS-5 | No | 0.474 |
| Mo2S2 | 4/mmm | - | 4.633 | - | - | - | 3.752 | 3.752 | P4/nmm | 2MoS-3 | No | 0.316 |
| Mo2S2 | -6m2 | - | 5.441 | - | - | - | 3.081 | 3.081 | P-6m2 | 2MoS-6 | No | 0.511 |
| Mo2S2 | -3m | - | 4.582 | - | - | 0 | 3.86 | 3.86 | P-3m1 | 2MoS-4 | No | 0.417 |
| Pd2Se2 | -6m2 | - | 5.183 | - | - | - | 3.779 | 3.779 | P-6m2 | 2PdSe-5 | No | 0.4 |
| Pd2Se2 | -3m | - | 4.871 | - | - | 0 | 4.026 | 4.028 | P-3m1 | 2PdSe-3 | Yes | 0.183 |
| Pd2Se2 | -3m | - | 5.192 | - | - | - | 3.791 | 3.791 | P-3m1 | 2PdSe-6 | No | 0.406 |
| AgSe2 | -42m | - | 5.2 | - | - | - | 2.874 | 2.874 | P-4m2 | 1AgSe2-1 | Yes | 0.157 |
| CrS2 | -42m | 1.999 | 5.473 | - | - | - | 3.588 | 3.588 | P-4m2 | 1CrS2-3 | Yes | 0.319 |
| CrS2 | -3m | - | 5.081 | - | - | - | 3.065 | 3.065 | P-3m1 | 1CrS2-2 | No | 0.175 |
| CrS2 | -6m2 | - | 5.403 | -1.464 | -0.113 | 1.351 | 3.048 | 3.048 | P-6m2 | 1CrS2-1 | Yes | 0 |
| Fe2S4 | 2/m | - | 5.392 | -2.5 | -1.169 | 1.331 | 3.227 | 5.324 | P2_1/m | 2FeS2-1 | Yes | 0.148 |
| Pd2Se4 | 2/m | - | 4.747 | -3.45 | -1.402 | 2.048 | 5.75 | 5.92 | P2_1/c | 2PdSe2-1 | Yes | 0.004 |
| IrS2 | -6m2 | - | 4.82 | - | - | - | 3.399 | 3.399 | P-6m2 | 1IrS2-3 | No | 0.558 |
| IrS2 | -3m | - | 5.725 | - | - | - | 3.558 | 3.558 | P-3m1 | 1IrS2-1 | No | 0.177 |
| IrS2 | -42m | - | 5.154 | - | - | - | 3.058 | 3.058 | P-4m2 | 1IrS2-2 | No | 0.517 |
| MoS2 | -42m | - | 5.08 | - | - | - | 3.697 | 3.697 | P-4m2 | 1MoS2-3 | Yes | 0.615 |
| MoS2 | -3m | - | 5.078 | - | - | - | 3.188 | 3.188 | P-3m1 | 1MoS2-2 | No | 0.277 |
| MoS2 | -6m2 | - | 5.097 | -1.454 | 0.633 | 2.087 | 3.184 | 3.184 | P-6m2 | 1MoS2-1 | Yes | 0 |
| Nb2Te4 | 4/mmm | 2.946 | 4.9 | - | - | - | 7.141 | 7.141 | P4/mbm | 2NbTe2-1 | No | 0.842 |
| Re2S4 | 2/m | - | 5.025 | - | - | - | 3.148 | 5.73 | P2_1/m | 2ReS2-1 | No | 0.097 |
| Ru2Te4 | 2/m | - | 4.354 | -0.63 | 0.441 | 1.071 | 3.815 | 6.144 | P2_1/m | 2RuTe2-1 | Yes | 0.117 |
| Ru2Te4 | 4/mmm | - | 5.057 | -2.71 | -2.49 | 0.22 | 6.651 | 6.649 | P4/mbm | 2RuTe2-2 | No | 0.678 |
| ScS2 | -6m2 | 1 | 5.679 | -3.664 | -1.774 | 1.89 | 3.788 | 3.788 | P-6m2 | 1ScS2-2 | Yes | 0.395 |
| ScS2 | -42m | - | 7.128 | - | - | - | 3.679 | 3.679 | P-4m2 | 1ScS2-3 | No | 0.711 |
| ScS2 | -3m | - | 6.717 | - | - | - | 3.744 | 3.744 | P-3m1 | 1ScS2-1 | Yes | 0.377 |
| ZrTe2 | -42m | - | 4.875 | -2.777 | -1.131 | 1.646 | 4.307 | 4.307 | P-4m2 | 1ZrTe2-3 | Yes | 0.373 |
| ZrTe2 | -3m | - | 4.829 | - | - | - | 3.968 | 3.968 | P-3m1 | 1ZrTe2-1 | Yes | 0.012 |
| ZrTe2 | -6m2 | - | 5.278 | -1.479 | -0.643 | 0.836 | 3.923 | 3.923 | P-6m2 | 1ZrTe2-2 | Yes | 0.11 |
| Mn2Te6 | 2/m | 4.954 | 4.674 | - | - | - | 6.161 | 3.644 | P2_1/m | 2MnTe3-1 | No | 0.126 |
| Ta2Se6 | mmm | - | 4.588 | - | - | - | 5.385 | 3.496 | Pmmn | 2TaSe3-1 | Yes | 0.046 |
| Au2S2 | -6m2 | - | 6.085 | - | - | - | 3.992 | 3.992 | P-6m2 | 2AuS-3 | No | 0.368 |
| Au2S2 | 2/m | - | 5.372 | -4.101 | -2.091 | 2.01 | 3.56 | 6.254 | P2/m | 2AuS-1 | Yes | 0 |
| Au2S2 | -3m | - | 6.096 | - | - | - | 3.998 | 3.998 | P-3m1 | 2AuS-2 | No | 0.363 |
| Cd2S2 | 4/mmm | - | 4.822 | -2.875 | -2.773 | 0.102 | 5.188 | 5.188 | P4/mmm | 2CdS-2 | No | 0.425 |
| Cd2S2 | -3m | - | 5.224 | -3.706 | -1.01 | 2.696 | 4.315 | 4.318 | P-3m1 | 2CdS-1 | Yes | 0.134 |
| Co2S2 | 4/mmm | 0.96 | 4.6 | - | - | - | 3.639 | 3.639 | P4/nmm | 2CoS-1 | Yes | 0.051 |
| Co2S2 | -6m2 | - | 4.887 | - | - | - | 3.123 | 3.123 | P-6m2 | 2CoS-5 | No | 0.378 |
| Co2S2 | -3m | - | 4.774 | - | - | 0 | 3.579 | 3.58 | P-3m1 | 2CoS-2 | Yes | 0.072 |
| Co2S2 | -3m | - | 5.156 | - | - | - | 3.461 | 3.461 | P-3m1 | 2CoS-4 | No | 0.374 |
| Co2S2 | 4/mmm | 0.508 | 5.369 | - | - | - | 3.441 | 3.442 | P4/mmm | 2CoS-3 | Yes | 0.227 |
| Nb2S2 | -3m | - | 5.061 | - | - | - | 3.241 | 3.241 | P-3m1 | 2NbS-5 | No | 0.56 |
| Nb2S2 | -3m | - | 3.843 | - | - | 0 | 3.95 | 3.95 | P-3m1 | 2NbS-2 | Yes | 0.323 |
| Nb2S2 | 4/mmm | - | 5.036 | - | - | 0 | 3.872 | 3.873 | P4/mmm | 2NbS-4 | No | 0.477 |
| Nb2S2 | 4/mmm | - | 5.396 | - | - | - | 3.89 | 3.89 | P4/nmm | 2NbS-3 | No | 0.402 |
| Nb2S2 | -6m2 | - | 5.298 | - | - | - | 3.205 | 3.205 | P-6m2 | 2NbS-6 | No | 0.58 |
| Pt2Te2 | 4/mmm | - | 3.984 | - | - | 0 | 4.09 | 4.093 | P4/mmm | 2PtTe-3 | No | 0.179 |
| Pt2Te2 | -3m | - | 4.255 | - | - | 0 | 4.034 | 4.036 | P-3m1 | 2PtTe-2 | Yes | 0.164 |
| Pt2Te2 | -3m | - | 4.554 | - | - | - | 4.198 | 4.198 | P-3m1 | 2PtTe-6 | No | 0.415 |
| Pt2Te2 | 4/mmm | - | 4.104 | - | - | - | 3.987 | 3.987 | P4/nmm | 2PtTe-4 | No | 0.301 |
| Pt2Te2 | -6m2 | - | 4.722 | - | - | - | 4.408 | 4.408 | P-6m2 | 2PtTe-5 | Yes | 0.372 |
| Pt2Te2 | -3m | - | 4.522 | - | - | - | 3.978 | 3.978 | P-3m1 | 2PtTe-1 | Yes | 0.048 |
| Ag2Te4 | 2/m | - | 4.595 | - | - | - | 6.608 | 6.896 | P2_1/c | 2AgTe2-1 | No | 0.129 |
| Au2Se4 | 2/m | - | 4.62 | - | - | - | 5.921 | 6.617 | P2_1/c | 2AuSe2-1 | No | 0.11 |
| Co2Se4 | 2/m | - | 5.217 | - | - | - | 3.398 | 5.769 | P2_1/m | 2CoSe2-1 | No | 0.044 |
| FeS2 | -42m | - | 5.366 | - | - | - | 3.468 | 3.468 | P-4m2 | 1FeS2-3 | Yes | 0.405 |
| FeS2 | -3m | 1.802 | 5.565 | - | - | - | 3.203 | 3.203 | P-3m1 | 1FeS2-1 | No | 0.205 |
| FeS2 | -6m2 | 1.428 | 5.116 | - | - | - | 3.154 | 3.154 | P-6m2 | 1FeS2-2 | Yes | 0.267 |
| HgSe2 | -3m | 0.49 | 5.807 | - | - | - | 3.867 | 3.867 | P-3m1 | 1HgSe2-3 | No | 0.527 |
| HgSe2 | -42m | - | 5.025 | - | - | - | 3.206 | 3.206 | P-4m2 | 1HgSe2-1 | No | 0.409 |
| HgSe2 | -6m2 | - | 4.764 | - | - | - | 4.452 | 4.452 | P-6m2 | 1HgSe2-2 | No | 0.478 |
| NbTe2 | -3m | - | 4.618 | - | - | - | 3.653 | 3.653 | P-3m1 | 1NbTe2-2 | Yes | 0.028 |
| NbTe2 | -42m | 0.99 | 4.57 | - | - | - | 4.137 | 4.137 | P-4m2 | 1NbTe2-3 | No | 0.495 |
| NbTe2 | -6m2 | 0.6 | 5.005 | - | - | - | 3.682 | 3.682 | P-6m2 | 1NbTe2-1 | Yes | 0.018 |
| Nb3Te6 | 2/m | - | 4.711 | - | - | - | 3.598 | 9.886 | C2/m | 3NbTe2-1 | Yes | 0 |
| PdSe2 | -6m2 | - | 3.655 | -1.026 | -0.76 | 0.266 | 4.001 | 4.001 | P-6m2 | 1PdSe2-2 | Yes | 0.268 |
| PdSe2 | -42m | - | 4.817 | - | - | - | 2.836 | 2.836 | P-4m2 | 1PdSe2-3 | No | 0.35 |
| PdSe2 | -3m | - | 5.057 | -2.152 | -1.245 | 0.906 | 3.735 | 3.735 | P-3m1 | 1PdSe2-1 | Yes | 0.026 |
| Re4S8 | -1 | - | 5.086 | -1.777 | 0.076 | 1.854 | 6.407 | 6.515 | P-1 | 4ReS2-1 | Yes | 0 |
| RhTe2 | -6m2 | - | 4.415 | - | - | - | 3.788 | 3.788 | P-6m2 | 1RhTe2-2 | No | 0.215 |
| RhTe2 | -42m | - | 4.166 | - | - | - | 3.342 | 3.342 | P-4m2 | 1RhTe2-3 | No | 0.414 |
| RhTe2 | -3m | - | 4.952 | - | - | - | 3.799 | 3.799 | P-3m1 | 1RhTe2-1 | No | 0.111 |
| TiS2 | -42m | - | 5.937 | -4.489 | -1.752 | 2.737 | 3.676 | 3.676 | P-4m2 | 1TiS2-3 | Yes | 0.236 |
| TiS2 | -6m2 | - | 6.399 | -3.397 | -1.382 | 2.015 | 3.345 | 3.345 | P-6m2 | 1TiS2-2 | No | 0.145 |
| TiS2 | -3m | - | 5.734 | -2.391 | -1.207 | 1.184 | 3.414 | 3.414 | P-3m1 | 1TiS2-1 | No | 0 |
| V2Te4 | 4/mmm | 4.427 | 4.848 | - | - | - | 6.899 | 6.899 | P4/mbm | 2VTe2-3 | Yes | 0.504 |
| Ta2Te6 | mmm | - | 4.226 | - | - | - | 5.947 | 3.737 | Pmmn | 2TaTe3-1 | Yes | 0.074 |
| W2S6 | mmm | - | 4.848 | - | - | - | 4.976 | 3.201 | Pmmn | 2WS3-1 | No | 0.39 |
| Zr2S6 | mmm | - | 5.432 | -1.624 | 0.578 | 2.202 | 5.192 | 3.645 | Pmmn | 2ZrS3-1 | Yes | 0 |
| Cu2Se2 | 2/m | - | 5.189 | -3.683 | -2.567 | 1.116 | 3.524 | 5.183 | P2/m | 2CuSe-4 | Yes | 0.101 |
| Cu2Se2 | -3m | - | 6.168 | - | - | - | 3.811 | 3.811 | P-3m1 | 2CuSe-6 | No | 0.265 |
| Cu2Se2 | -6m2 | - | 4.179 | - | - | - | 4.093 | 4.093 | P-6m2 | 2CuSe-5 | Yes | 0.158 |
| Cu2Se2 | 4/mmm | - | 5.665 | - | - | - | 3.712 | 3.712 | P4/nmm | 2CuSe-3 | Yes | 0.069 |
| Cu2Se2 | -3m | - | 5.586 | - | - | 0 | 3.763 | 3.764 | P-3m1 | 2CuSe-1 | Yes | 0.056 |
| Fe2S2 | 4/mmm | 2.924 | 5.163 | - | - | - | 3.447 | 3.447 | P4/mmm | 2FeS-3 | No | 0.239 |
| Fe2S2 | -6m2 | 0.323 | 4.786 | - | - | - | 3.418 | 3.418 | P-6m2 | 2FeS-5 | No | 0.401 |
| Fe2S2 | 4/mmm | - | 4.861 | - | - | - | 3.599 | 3.599 | P4/nmm | 2FeS-1 | Yes | 0 |
| Fe2S2 | -3m | - | 4.968 | - | - | - | 3.393 | 3.393 | P-3m1 | 2FeS-4 | No | 0.39 |
| Ir2Te2 | -6m2 | - | 4.048 | - | - | - | 4.002 | 4.002 | P-6m2 | 2IrTe-6 | No | 0.544 |
| Ir2Te2 | 4/mmm | - | 3.525 | - | - | - | 4.053 | 4.053 | P4/nmm | 2IrTe-1 | No | 0.224 |
| Ir2Te2 | -3m | - | 3.819 | - | - | 0 | 4.161 | 4.162 | P-3m1 | 2IrTe-4 | Yes | 0.331 |
| Ir2Te2 | -3m | - | 4.06 | - | - | - | 4.059 | 4.059 | P-3m1 | 2IrTe-5 | No | 0.519 |
| Ir2Te2 | mm2 | - | 3.992 | - | - | 0 | 3.934 | 3.958 | Pmm2 | 2IrTe-2 | No | 0.259 |
| AgTe2 | -42m | - | 4.724 | - | - | - | 3.19 | 3.19 | P-4m2 | 1AgTe2-1 | Yes | 0.052 |
| AuSe2 | -6m2 | - | 5.223 | - | - | - | 3.632 | 3.632 | P-6m2 | 1AuSe2-3 | Yes | 0.443 |
| AuSe2 | -3m | - | 5.052 | - | - | - | 3.806 | 3.806 | P-3m1 | 1AuSe2-1 | No | 0.263 |
| AuSe2 | -42m | - | 4.959 | - | - | - | 2.882 | 2.882 | P-4m2 | 1AuSe2-2 | No | 0.288 |
| CoSe2 | -42m | - | 4.717 | - | - | - | 3.033 | 3.033 | P-4m2 | 1CoSe2-3 | No | 0.335 |
| CoSe2 | -6m2 | - | 4.689 | - | - | - | 3.372 | 3.372 | P-6m2 | 1CoSe2-2 | No | 0.168 |
| CoSe2 | -3m | - | 5.369 | - | - | - | 3.368 | 3.368 | P-3m1 | 1CoSe2-1 | No | 0.053 |
| Cu2S4 | 2/m | - | 4.914 | - | - | - | 5.555 | 5.69 | P2_1/c | 2CuS2-1 | No | 0.11 |
| HfS2 | -6m2 | - | 6.354 | -3.517 | -1.479 | 2.038 | 3.543 | 3.543 | P-6m2 | 1HfS2-2 | No | 0.217 |
| HfS2 | -42m | - | 5.605 | -4.559 | -1.408 | 3.151 | 3.879 | 3.879 | P-4m2 | 1HfS2-3 | Yes | 0.333 |
| HfS2 | -3m | - | 5.726 | -3.129 | -0.981 | 2.148 | 3.652 | 3.652 | P-3m1 | 1HfS2-1 | Yes | 0 |
| MnTe2 | -42m | 3 | 4.969 | - | - | - | 4.042 | 4.042 | P-4m2 | 1MnTe2-3 | No | 0.379 |
| MnTe2 | -6m2 | 2.504 | 4.428 | - | - | - | 3.66 | 3.66 | P-6m2 | 1MnTe2-2 | Yes | 0.096 |
| MnTe2 | -3m | 2.708 | 4.574 | - | - | - | 3.735 | 3.735 | P-3m1 | 1MnTe2-1 | Yes | 0 |
| Os2S4 | 4/mmm | - | 5.386 | -2.895 | -2.773 | 0.122 | 5.735 | 5.732 | P4/mbm | 2OsS2-2 | No | 0.635 |
| Os2S4 | 2/m | - | 5.13 | -1.83 | -0.443 | 1.387 | 3.522 | 5.537 | P2_1/m | 2OsS2-1 | Yes | 0.081 |
| Pt2Te4 | 2/m | - | 4.433 | -2.735 | -0.735 | 2.001 | 6.112 | 6.406 | P2_1/c | 2PtTe2-1 | Yes | 0.144 |
| ReS2 | -3m | - | 5.08 | - | - | - | 3.094 | 3.094 | P-3m1 | 1ReS2-2 | No | 0.372 |
| ReS2 | -42m | - | 4.74 | - | - | - | 3.632 | 3.632 | P-4m2 | 1ReS2-3 | Yes | 0.566 |
| Rh2S4 | 2/m | - | 5.376 | - | - | 0 | 5.683 | 5.687 | P2_1/c | 2RhS2-2 | No | 0.247 |
| Rh2S4 | 2/m | - | 5.887 | - | - | - | 3.529 | 5.804 | P2_1/m | 2RhS2-1 | No | 0.123 |
| RuTe2 | -42m | - | 4.228 | - | - | - | 3.292 | 3.292 | P-4m2 | 1RuTe2-3 | No | 0.508 |
| RuTe2 | -3m | - | 4.736 | - | - | - | 3.767 | 3.767 | P-3m1 | 1RuTe2-2 | No | 0.329 |
| RuTe2 | -6m2 | - | 4.244 | - | - | - | 3.694 | 3.694 | P-6m2 | 1RuTe2-1 | No | 0.304 |
| TaSe2 | -6m2 | - | 5.361 | - | - | - | 3.471 | 3.471 | P-6m2 | 1TaSe2-1 | No | 0 |
| TaSe2 | -42m | - | 4.391 | - | - | - | 3.815 | 3.815 | P-4m2 | 1TaSe2-3 | Yes | 0.472 |
| TaSe2 | -3m | - | 4.696 | - | - | - | 3.503 | 3.503 | P-3m1 | 1TaSe2-2 | Yes | 0.023 |
| VTe2 | -3m | 0.885 | 4.576 | - | - | - | 3.6 | 3.6 | P-3m1 | 1VTe2-2 | No | 0.018 |
| VTe2 | -6m2 | 0.995 | 4.968 | -0.69 | 0.164 | 0.854 | 3.598 | 3.598 | P-6m2 | 1VTe2-1 | Yes | 0.016 |
| VTe2 | -42m | 1 | 5.102 | - | - | - | 4.062 | 4.062 | P-4m2 | 1VTe2-3 | Yes | 0.338 |
| W2Te6 | 2/m | - | 4.371 | - | - | - | 6.662 | 3.509 | P2_1/m | 2WTe3-1 | No | 0.188 |
| Zr2Se6 | mmm | - | 5.021 | -0.581 | 0.45 | 1.031 | 5.479 | 3.774 | Pmmn | 2ZrSe3-1 | Yes | 0 |
| Cd2Se2 | 4/mmm | - | 4.91 | - | - | - | 5.422 | 5.422 | P4/mmm | 2CdSe-3 | No | 0.422 |
| Cd2Se2 | -3m | - | 5.141 | -3.17 | -0.972 | 2.197 | 4.453 | 4.455 | P-3m1 | 2CdSe-1 | Yes | 0.11 |
| Os2Se2 | -6m2 | - | 4.125 | - | - | - | 3.85 | 3.85 | P-6m2 | 2OsSe-4 | No | 0.682 |
| Os2Se2 | -3m | - | 4.226 | - | - | - | 3.266 | 3.273 | P-3m1 | 2OsSe-2 | No | 0.428 |
| Os2Se2 | m | - | 4.138 | - | - | - | 2.932 | 4.233 | Pm | 2OsSe-3 | No | 0.572 |
| Os2Se2 | 4/mmm | - | 3.811 | - | - | - | 3.975 | 3.975 | P4/nmm | 2OsSe-1 | Yes | 0.383 |
| Pd2Te2 | -3m | - | 4.806 | - | - | - | 3.976 | 3.976 | P-3m1 | 2PdTe-5 | No | 0.427 |
| Pd2Te2 | -6m2 | - | 4.788 | - | - | - | 3.98 | 3.98 | P-6m2 | 2PdTe-4 | No | 0.422 |
| Pd2Te2 | -3m | - | 4.485 | - | - | 0 | 4.177 | 4.18 | P-3m1 | 2PdTe-2 | Yes | 0.134 |
| Rh2S2 | 4/mmm | 0.575 | 4.401 | - | - | - | 3.934 | 3.934 | P4/nmm | 2RhS-2 | No | 0.165 |
| Rh2S2 | -6m2 | - | 4.95 | -0.23 | -0.12 | 0.11 | 3.222 | 3.222 | P-6m2 | 2RhS-3 | No | 0.201 |
| Rh2S2 | -3m | - | 4.808 | - | - | - | 3.28 | 3.28 | P-3m1 | 2RhS-4 | No | 0.228 |
| Rh2S2 | -3m | - | 4.783 | - | - | 0 | 3.844 | 3.847 | P-3m1 | 2RhS-1 | Yes | 0.161 |
| Rh2S2 | 2/m | - | 5.59 | - | - | - | 2.813 | 4.759 | P2/m | 2RhS-5 | No | 0.41 |
| Ru2S2 | -3m | - | 4.504 | - | - | 0 | 3.798 | 3.799 | P-3m1 | 2RuS-2 | Yes | 0.278 |
| Ru2S2 | -3m | - | 4.834 | - | - | - | 3.475 | 3.475 | P-3m1 | 2RuS-3 | Yes | 0.335 |
| Ru2S2 | -6m2 | - | 4.671 | - | - | - | 3.496 | 3.496 | P-6m2 | 2RuS-4 | No | 0.344 |
| Ru2S2 | m | - | 5.173 | - | - | - | 2.842 | 4.765 | Pm | 2RuS-5 | No | 0.599 |
| Ru2S2 | 4/mmm | - | 4.721 | - | - | - | 3.884 | 3.884 | P4/nmm | 2RuS-1 | Yes | 0.05 |
| Cd2S4 | 2/m | - | 4.115 | -3.14 | -0.762 | 2.378 | 5.958 | 5.901 | P2_1/c | 2CdS2-2 | Yes | 0.292 |
| CuS2 | -42m | - | 5.551 | - | - | - | 2.663 | 2.663 | P-4m2 | 1CuS2-2 | No | 0.365 |
| CuS2 | -3m | - | 5.875 | - | - | - | 3.373 | 3.373 | P-3m1 | 1CuS2-3 | Yes | 0.424 |
| CuS2 | -6m2 | 0.836 | 4.427 | - | - | - | 3.739 | 3.739 | P-6m2 | 1CuS2-1 | Yes | 0.301 |
| Ir2Se4 | 2/m | - | 5.148 | - | - | - | 3.732 | 6.118 | P2_1/m | 2IrSe2-1 | No | 0.12 |
| Ir2Se4 | 2/m | - | 5.296 | - | - | 0 | 5.98 | 6.034 | P2_1/c | 2IrSe2-2 | No | 0.473 |
| PtTe2 | -42m | - | 4.261 | - | - | - | 3.457 | 3.457 | P-4m2 | 1PtTe2-3 | No | 0.487 |
| PtTe2 | -3m | - | 4.133 | -0.68 | -0.08 | 0.599 | 4.016 | 4.016 | P-3m1 | 1PtTe2-1 | Yes | 0.027 |
| PtTe2 | -6m2 | - | 4 | - | - | - | 3.899 | 3.899 | P-6m2 | 1PtTe2-2 | Yes | 0.326 |
| ScSe2 | -3m | - | 6.21 | - | - | - | 3.867 | 3.867 | P-3m1 | 1ScSe2-1 | Yes | 0.079 |
| ScSe2 | -6m2 | 0.998 | 5.338 | -3.034 | -1.544 | 1.49 | 3.945 | 3.945 | P-6m2 | 1ScSe2-2 | Yes | 0.126 |
| ScSe2 | -42m | - | 6.398 | - | - | - | 3.749 | 3.749 | P-4m2 | 1ScSe2-3 | No | 0.373 |
| W2Se4 | 2/m | - | 4.938 | -0.561 | -0.443 | 0.118 | 3.304 | 5.95 | P2_1/m | 2WSe2-1 | Yes | 0.092 |
| W2Se4 | 4/mmm | 3.965 | 4.524 | - | - | - | 6.442 | 6.442 | P4/mbm | 2WSe2-2 | No | 1.117 |
| Mo2S6 | mmm | - | 4.865 | - | - | - | 4.877 | 3.212 | Pmmn | 2MoS3-2 | No | 0.355 |
| Nb2S6 | mmm | - | 4.761 | - | - | - | 4.944 | 3.35 | Pmmn | 2NbS3-1 | No | 0.024 |
| Zr2Te6 | mmm | - | 4.827 | - | - | - | 5.958 | 3.953 | Pmmn | 2ZrTe3-1 | No | 0 |
| Ta4Se12 | 2/m | - | 5.02 | - | - | - | 3.519 | 12.267 | P2_1/m | 4TaSe3-1 | Yes | 0 |
| Au2Se2 | -3m | - | 5.827 | - | - | - | 4.151 | 4.151 | P-3m1 | 2AuSe-5 | No | 0.344 |
| Au2Se2 | -6m2 | - | 5.77 | - | - | - | 4.151 | 4.151 | P-6m2 | 2AuSe-6 | No | 0.348 |
| Au2Se2 | -3m | - | 5.221 | - | - | 0 | 4.062 | 4.062 | P-3m1 | 2AuSe-4 | No | 0.319 |
| Au2Se2 | 2/m | - | 5.05 | -3.516 | -1.954 | 1.561 | 3.732 | 6.326 | P2/m | 2AuSe-1 | Yes | 0.007 |
| Mo2Se2 | -3m | - | 4.316 | - | - | 0 | 3.94 | 3.941 | P-3m1 | 2MoSe-3 | No | 0.38 |
| Mo2Se2 | 4/mmm | - | 4.099 | - | - | - | 3.805 | 3.805 | P4/nmm | 2MoSe-2 | No | 0.263 |
| Mo2Se2 | -6m2 | - | 4.921 | - | - | - | 3.134 | 3.134 | P-6m2 | 2MoSe-5 | No | 0.56 |
| Mo2Se2 | -3m | - | 4.914 | - | - | 0 | 3.126 | 3.125 | P-3m1 | 2MoSe-1 | Yes | 0.231 |
| Mo2Se2 | mm2 | - | 4.578 | - | - | - | 3.706 | 3.861 | Pmm2 | 2MoSe-4 | No | 0.4 |
| Ni2S2 | 4/mmm | - | 4.908 | - | - | - | 3.807 | 3.807 | P4/nmm | 2NiS-3 | Yes | 0.098 |
| Ni2S2 | -3m | - | 5.552 | - | - | 0 | 3.586 | 3.586 | P-3m1 | 2NiS-5 | Yes | 0.303 |
| Ni2S2 | 4/mmm | 0.964 | 5.215 | - | - | - | 3.538 | 3.539 | P4/mmm | 2NiS-2 | Yes | 0.09 |
| Ni2S2 | -3m | - | 5.087 | - | - | 0 | 3.612 | 3.613 | P-3m1 | 2NiS-1 | Yes | 0.042 |
| Ni2S2 | -6m2 | - | 5.312 | - | - | - | 3.648 | 3.648 | P-6m2 | 2NiS-4 | Yes | 0.261 |
| Fe2Se4 | 2/m | - | 4.849 | -1.663 | -0.609 | 1.053 | 3.393 | 5.567 | P2_1/m | 2FeSe2-1 | Yes | 0.061 |
| Fe2Se4 | 4/mmm | 5.871 | 4.935 | - | - | - | 6.121 | 6.121 | P4/mbm | 2FeSe2-2 | No | 0.435 |
| CdS2 | -1 | - | 4.387 | -3.828 | -1.449 | 2.38 | 4.024 | 5.484 | P-1 | 1CdS2-1 | No | 0.383 |
| CdS2 | -42m | - | 5.96 | - | - | - | 3.092 | 3.091 | P-4m2 | 1CdS2-2 | No | 0.728 |
| Hg2Te4 | 2/m | - | 4.668 | - | - | - | 4.07 | 5.66 | P2_1/m | 2HgTe2-2 | No | 0.357 |
| Ni2S4 | 2/m | - | 4.868 | -4.063 | -1.833 | 2.231 | 5.226 | 5.339 | P2_1/c | 2NiS2-1 | Yes | 0.035 |
| OsS2 | -42m | - | 5.104 | - | - | - | 2.988 | 2.988 | P-4m2 | 1OsS2-3 | No | 0.497 |
| OsS2 | -3m | - | 5.465 | - | - | - | 3.511 | 3.511 | P-3m1 | 1OsS2-1 | No | 0.366 |
| OsS2 | -6m2 | - | 4.411 | - | - | - | 3.365 | 3.365 | P-6m2 | 1OsS2-2 | No | 0.453 |
| Pd2Te4 | 2/m | - | 4.518 | -2.855 | -1.112 | 1.743 | 6.139 | 6.428 | P2_1/c | 2PdTe2-1 | Yes | 0.093 |
| Rh4S8 | -1 | - | 5.694 | -2.999 | -1.597 | 1.402 | 6.414 | 7.053 | P-1 | 4RhS2-1 | Yes | 0.066 |
| Co2S6 | mm2 | - | 5.577 | - | - | - | 5.489 | 3.144 | Pmn2_1 | 2CoS3-2 | No | 0.226 |
| Pt2S2 | -3m | - | 5.047 | - | - | - | 3.76 | 3.76 | P-3m1 | 2PtS-5 | No | 0.498 |
| Pt2S2 | -6m2 | - | 5.105 | - | - | - | 3.768 | 3.768 | P-6m2 | 2PtS-4 | No | 0.497 |
| Pt2S2 | -3m | - | 4.987 | - | - | 0 | 3.554 | 3.555 | P-3m1 | 2PtS-3 | No | 0.396 |
| Pt2S2 | 2/m | - | 5.525 | - | - | - | 3.407 | 4.471 | P2/m | 2PtS-1 | No | 0.316 |
| Pt2S2 | 4/mmm | - | 4.887 | - | - | - | 4.55 | 4.55 | P4/nmm | 2PtS-6 | No | 0.545 |
| Mo2Se6 | 2/m | - | 4.883 | - | - | - | 5.994 | 3.274 | P2_1/m | 2MoSe3-1 | No | 0.241 |
| Nb2Se6 | mmm | - | 4.662 | - | - | - | 5.342 | 3.485 | Pmmn | 2NbSe3-1 | Yes | 0.037 |
| Ru2Se6 | 2/m | - | 4.465 | - | - | - | 7.192 | 3.568 | P2_1/m | 2RuSe3-1 | Unknown | 0.21 |
| Sc2S6 | mmm | - | 6.029 | - | - | - | 5.257 | 3.417 | Pmmn | 2ScS3-1 | Yes | 0.189 |
| PdTe2 | -6m2 | - | 3.781 | - | - | - | 4.037 | 4.037 | P-6m2 | 1PdTe2-2 | Yes | 0.227 |
| PdTe2 | -3m | - | 4.317 | - | - | - | 4.019 | 4.019 | P-3m1 | 1PdTe2-1 | Yes | 0.086 |
| PdTe2 | -42m | - | 4.512 | - | - | - | 3.156 | 3.156 | P-4m2 | 1PdTe2-3 | No | 0.31 |
| Nb4S12 | 2/m | - | 4.689 | -2.029 | -0.999 | 1.03 | 5.023 | 6.772 | P2_1/m | 4NbS3-2 | Yes | 0.002 |
| Cd2Te2 | 4/mmm | - | 4.995 | - | - | - | 5.794 | 5.794 | P4/mmm | 2CdTe-3 | No | 0.417 |
| Cd2Te2 | -3m | - | 4.778 | -2.085 | -0.754 | 1.331 | 4.669 | 4.67 | P-3m1 | 2CdTe-1 | Yes | 0.094 |
| Cd2Te2 | -3m | - | 5.068 | -2.659 | -1.182 | 1.478 | 4.698 | 4.698 | P-3m1 | 2CdTe-2 | No | 0.242 |
| Hf2S2 | -3m | 0.718 | 4.806 | - | - | - | 3.56 | 3.56 | P-3m1 | 2HfS-3 | No | 0.535 |
| Hf2S2 | 4/mmm | - | 3.942 | - | - | - | 4.325 | 4.325 | P4/nmm | 2HfS-4 | No | 0.543 |
| Hf2S2 | -6m2 | 0.426 | 4.783 | - | - | - | 3.555 | 3.555 | P-6m2 | 2HfS-2 | No | 0.532 |
| Hf2S2 | -3m | - | 3.355 | 0.299 | 0.434 | 0.135 | 4.148 | 4.149 | P-3m1 | 2HfS-1 | Yes | 0.449 |
| Hf2S2 | 2/m | - | 4.866 | - | - | - | 3.448 | 5.317 | P2/m | 2HfS-5 | No | 0.731 |
| Au2Te4 | 2/m | - | 4.414 | - | - | - | 6.415 | 7.089 | P2_1/c | 2AuTe2-1 | No | 0.141 |
| Co2Te4 | 2/m | - | 4.832 | - | - | - | 3.636 | 6.256 | P2_1/m | 2CoTe2-1 | No | 0.071 |
| Cr2Se4 | 4/mmm | 5.168 | 4.845 | - | - | - | 6.225 | 6.225 | P4/mbm | 2CrSe2-2 | No | 0.431 |
| HgTe2 | -6m2 | - | 5.07 | - | - | - | 3.722 | 3.722 | P-6m2 | 1HgTe2-3 | No | 0.492 |
| HgTe2 | -42m | - | 4.401 | - | - | - | 3.506 | 3.506 | P-4m2 | 1HgTe2-1 | No | 0.277 |
| HgTe2 | -3m | - | 4.911 | - | - | - | 3.705 | 3.705 | P-3m1 | 1HgTe2-2 | No | 0.384 |
| Ir4Se8 | -1 | - | 4.823 | -1.915 | -0.347 | 1.568 | 6.692 | 7.445 | P-1 | 4IrSe2-1 | Yes | 0.036 |
| Mo2Se4 | 4/mmm | 4.032 | 4.635 | - | - | - | 6.428 | 6.428 | P4/mbm | 2MoSe2-2 | No | 0.964 |
| Mo2Se4 | 2/m | - | 5.1 | -0.732 | -0.624 | 0.108 | 3.285 | 5.963 | P2_1/m | 2MoSe2-1 | Yes | 0.109 |
| NiS2 | -42m | - | 5.525 | - | - | - | 2.924 | 2.924 | P-4m2 | 1NiS2-3 | No | 0.451 |
| NiS2 | -6m2 | - | 3.747 | - | - | - | 3.542 | 3.542 | P-6m2 | 1NiS2-2 | Yes | 0.246 |
| Re2Se4 | 2/m | - | 4.438 | - | - | - | 3.287 | 5.934 | P2_1/m | 2ReSe2-1 | No | 0.109 |
| RhS2 | -6m2 | - | 5.1 | - | - | - | 3.414 | 3.414 | P-6m2 | 1RhS2-2 | No | 0.392 |
| RhS2 | -42m | - | 5.411 | - | - | - | 3.065 | 3.065 | P-4m2 | 1RhS2-3 | No | 0.463 |
| RhS2 | -3m | - | 6.043 | - | - | - | 3.512 | 3.512 | P-3m1 | 1RhS2-1 | No | 0.162 |
| Ru2S4 | 2/m | - | 5.35 | -2.481 | -0.898 | 1.583 | 3.455 | 5.567 | P2_1/m | 2RuS2-1 | Yes | 0.167 |
| Ru2S4 | 4/mmm | - | 5.391 | -3.215 | -3.021 | 0.194 | 5.732 | 5.732 | P4/mbm | 2RuS2-2 | No | 0.584 |
| Co2Se6 | 2 | - | 5.213 | - | - | - | 4.898 | 3.571 | P2_1 | 2CoSe3-2 | No | 0.142 |
| Ta2Te4 | 4/mmm | 2.507 | 4.853 | - | - | - | 7.145 | 7.148 | P4/mbm | 2TaTe2-1 | No | 0.92 |
| WSe2 | -3m | - | 4.317 | - | - | - | 3.291 | 3.291 | P-3m1 | 1WSe2-2 | No | 0.258 |
| WSe2 | -6m2 | - | 4.233 | -0.303 | 1.427 | 1.73 | 3.319 | 3.319 | P-6m2 | 1WSe2-1 | Yes | 0 |
| WSe2 | -42m | - | 4.397 | - | - | - | 3.643 | 3.643 | P-4m2 | 1WSe2-3 | No | 0.715 |
| Mo2Te6 | 2/m | - | 4.477 | - | - | - | 6.535 | 3.391 | P2_1/m | 2MoTe3-1 | No | 0.171 |
| Nb2Te6 | mmm | - | 4.279 | - | - | - | 5.942 | 3.694 | Pmmn | 2NbTe3-1 | No | 0.065 |
| Ru2Te6 | 2/m | - | 4.693 | - | - | - | 6.16 | 3.607 | P2_1/m | 2RuTe3-1 | No | 0.239 |
| Sc2Se6 | mmm | - | 5.642 | - | - | - | 5.544 | 3.525 | Pmmn | 2ScSe3-1 | No | 0 |
| Cu2Te2 | mmm | - | 4.541 | - | - | - | 3.223 | 4.034 | Pmmn | 2CuTe-1 | Yes | 0 |
| Cu2Te2 | -3m | - | 4.96 | - | - | 0 | 3.893 | 3.894 | P-3m1 | 2CuTe-2 | Yes | 0.026 |
| Cu2Te2 | -3m | 0.994 | 5.284 | - | - | - | 4.265 | 4.269 | P-3m1 | 2CuTe-7 | No | 0.311 |
| Cu2Te2 | -6m2 | - | 3.867 | - | - | - | 4.393 | 4.393 | P-6m2 | 2CuTe-5 | Yes | 0.181 |
| Cu2Te2 | 4/mmm | - | 4.496 | - | - | 0 | 3.888 | 3.89 | P4/mmm | 2CuTe-4 | Yes | 0.087 |
| Hf2Te10 | mmm | - | 4.459 | -2.252 | -2.127 | 0.126 | 4.024 | 13.814 | Pmmn | 2HfTe5-1 | Yes | 0 |
| Y2Te6 | 4/mmm | - | 4.757 | - | - | - | 4.331 | 4.331 | P4/nmm | 2YTe3-1 | Yes | 0 |
| Co2Se2 | -3m | - | 4.416 | - | - | 0 | 3.741 | 3.742 | P-3m1 | 2CoSe-2 | Yes | 0.058 |
| Co2Se2 | -6m2 | - | 4.377 | - | - | - | 3.312 | 3.312 | P-6m2 | 2CoSe-5 | No | 0.383 |
| Co2Se2 | 4/mmm | - | 4.825 | - | - | - | 3.52 | 3.521 | P4/mmm | 2CoSe-3 | No | 0.109 |
| Co2Se2 | -3m | 1.104 | 4.863 | - | - | - | 3.64 | 3.64 | P-3m1 | 2CoSe-4 | No | 0.363 |
| Co2Se2 | 4/mmm | - | 4.208 | - | - | - | 3.646 | 3.646 | P4/nmm | 2CoSe-1 | Yes | 0.016 |
| Cr2S2 | -3m | 2.504 | 4.495 | - | - | - | 3.668 | 3.668 | P-3m1 | 2CrS-2 | Yes | 0.177 |
| Fe2Se2 | 4/mmm | 5.313 | 4.852 | - | - | - | 3.62 | 3.62 | P4/mmm | 2FeSe-3 | No | 0.115 |
| Fe2Se2 | -6m2 | - | 4.683 | - | - | - | 3.669 | 3.669 | P-6m2 | 2FeSe-5 | No | 0.417 |
| Fe2Se2 | -3m | 0.663 | 4.768 | - | - | - | 3.65 | 3.65 | P-3m1 | 2FeSe-4 | No | 0.391 |
| Fe2Se2 | 4/mmm | - | 4.379 | - | - | - | 3.68 | 3.68 | P4/nmm | 2FeSe-1 | Yes | 0.009 |
| Fe2Se2 | -3m | 5.162 | 4.817 | - | - | - | 3.676 | 3.677 | P-3m1 | 2FeSe-2 | Yes | 0.068 |
| Fe2Se2 | 4/mmm | 6.692 | 4.321 | - | - | - | 4.889 | 4.889 | P4/nmm | 2FeSe-6 | Unknown | 0.65 |
| Nb2Se2 | 4/mmm | - | 5.503 | - | - | - | 3.86 | 3.86 | P4/nmm | 2NbSe-1 | No | 0.273 |
| Nb2Se2 | -3m | 0.351 | 4.692 | - | - | - | 3.329 | 3.329 | P-3m1 | 2NbSe-4 | No | 0.555 |
| Nb2Se2 | -3m | - | 3.986 | - | - | 0 | 4.05 | 4.051 | P-3m1 | 2NbSe-2 | Yes | 0.298 |
| Nb2Se2 | 4/mmm | - | 4.882 | - | - | 0 | 3.895 | 3.897 | P4/mmm | 2NbSe-3 | No | 0.345 |
| Nb2Se2 | -6m2 | - | 5.048 | - | - | - | 3.218 | 3.218 | P-6m2 | 2NbSe-5 | No | 0.574 |
| Re2S2 | -6m2 | - | 4.895 | - | - | - | 2.955 | 2.955 | P-6m2 | 2ReS-1 | No | 0.458 |
| Re2S2 | 4/mmm | - | 4.473 | - | - | - | 3.795 | 3.795 | P4/nmm | 2ReS-3 | No | 0.547 |
| Re2S2 | -3m | - | 3.726 | 0.294 | 0.613 | 0.319 | 3.88 | 3.88 | P-3m1 | 2ReS-2 | Yes | 0.546 |
| Re2S2 | m | - | 4.654 | - | - | - | 2.981 | 4.27 | Pm | 2ReS-4 | No | 0.615 |
| Sc2Se2 | -3m | - | 4.921 | - | - | - | 3.851 | 3.851 | P-3m1 | 2ScSe-4 | No | 0.564 |
| Sc2Se2 | -3m | 0.792 | 3.513 | - | - | - | 4.381 | 4.383 | P-3m1 | 2ScSe-1 | No | 0.333 |
| Sc2Se2 | -6m2 | - | 4.866 | -0.954 | -0.021 | 0.933 | 3.872 | 3.872 | P-6m2 | 2ScSe-3 | No | 0.548 |
| Sc2Se2 | 4/mmm | - | 3.712 | - | - | - | 4.685 | 4.685 | P4/nmm | 2ScSe-2 | Yes | 0.34 |
| Sc2Se2 | 4/mmm | - | 5.242 | - | - | 0 | 4.297 | 4.295 | P4/mmm | 2ScSe-5 | No | 0.571 |
| AuTe2 | -3m | - | 4.632 | - | - | - | 4.028 | 4.028 | P-3m1 | 1AuTe2-2 | No | 0.224 |
| AuTe2 | -42m | - | 4.535 | - | - | - | 3.206 | 3.206 | P-4m2 | 1AuTe2-1 | No | 0.172 |
| AuTe2 | -6m2 | - | 4.55 | - | - | - | 3.852 | 3.852 | P-6m2 | 1AuTe2-3 | Yes | 0.311 |
| Cd2Se4 | 2/m | - | 4.356 | -2.948 | -0.712 | 2.236 | 5.631 | 6.004 | C2/m | 2CdSe2-1 | No | 0.24 |
| CoTe2 | -3m | - | 4.901 | - | - | - | 3.623 | 3.623 | P-3m1 | 1CoTe2-1 | No | 0.073 |
| CoTe2 | -42m | - | 4.379 | - | - | - | 3.287 | 3.287 | P-4m2 | 1CoTe2-3 | No | 0.367 |
| CoTe2 | -6m2 | - | 4.336 | - | - | - | 3.613 | 3.613 | P-6m2 | 1CoTe2-2 | Yes | 0.16 |
| Cu2Se4 | 2/m | - | 4.838 | - | - | - | 5.851 | 6.045 | P2_1/c | 2CuSe2-1 | No | 0.098 |
| FeSe2 | -6m2 | 1.918 | 4.772 | - | - | - | 3.294 | 3.294 | P-6m2 | 1FeSe2-2 | No | 0.152 |
| FeSe2 | -42m | - | 4.92 | - | - | - | 3.667 | 3.667 | P-4m2 | 1FeSe2-3 | Yes | 0.382 |
| FeSe2 | -3m | 1.692 | 4.974 | - | - | - | 3.364 | 3.364 | P-3m1 | 1FeSe2-1 | No | 0.106 |
| IrSe2 | -42m | - | 4.376 | - | - | - | 3.132 | 3.132 | P-4m2 | 1IrSe2-3 | No | 0.463 |
| IrSe2 | -3m | - | 5.262 | - | - | - | 3.709 | 3.709 | P-3m1 | 1IrSe2-1 | No | 0.155 |
| IrSe2 | -6m2 | - | 4.361 | - | - | - | 3.558 | 3.558 | P-6m2 | 1IrSe2-2 | No | 0.42 |
| Re4Se8 | -1 | - | 4.487 | -0.957 | 0.664 | 1.621 | 6.655 | 6.779 | P-1 | 4ReSe2-1 | Yes | 0 |
| Sc2Te4 | 4/mmm | - | 4.372 | - | - | 0 | 7.304 | 7.309 | P4/mbm | 2ScTe2-1 | No | 0.59 |
| TaTe2 | -3m | - | 4.381 | - | - | - | 3.691 | 3.691 | P-3m1 | 1TaTe2-2 | Yes | 0.049 |
| TaTe2 | -6m2 | - | 4.879 | - | - | - | 3.699 | 3.699 | P-6m2 | 1TaTe2-1 | No | 0.044 |
| TaTe2 | -42m | 0.615 | 4.279 | - | - | - | 4.076 | 4.076 | P-4m2 | 1TaTe2-3 | No | 0.537 |
| TiSe2 | -42m | - | 5.467 | -3.641 | -1.421 | 2.22 | 3.829 | 3.829 | P-4m2 | 1TiSe2-3 | Yes | 0.27 |
| TiSe2 | -3m | - | 5.288 | -0.884 | -0.737 | 0.147 | 3.536 | 3.536 | P-3m1 | 1TiSe2-1 | No | 0.009 |
| TiSe2 | -6m2 | - | 5.832 | -2.306 | -0.846 | 1.46 | 3.495 | 3.495 | P-6m2 | 1TiSe2-2 | Yes | 0.126 |
| V2S4 | 4/mmm | 3.649 | 4.982 | - | - | - | 5.934 | 5.934 | P4/mbm | 2VS2-1 | No | 0.649 |
| Co2Te6 | 2/m | - | 4.568 | - | - | - | 6.12 | 3.591 | P2_1/m | 2CoTe3-2 | Yes | 0.193 |
| Os2Se6 | 2/m | - | 4.948 | - | - | - | 5.501 | 3.516 | P2_1/m | 2OsSe3-1 | No | 0.248 |
| Sc2Te6 | 2/m | - | 4.981 | - | - | - | 5.987 | 3.77 | P2_1/m | 2ScTe3-1 | No | 0.081 |
| Os2Te2 | 4/mmm | - | 3.602 | - | - | - | 4.088 | 4.088 | P4/nmm | 2OsTe-1 | Yes | 0.422 |
| Os2Te2 | -3m | - | 4.215 | - | - | - | 3.764 | 3.765 | P-3m1 | 2OsTe-2 | Yes | 0.457 |
| Os2Te2 | -3m | - | 4.091 | - | - | - | 4.118 | 4.118 | P-3m1 | 2OsTe-4 | No | 0.729 |
| Os2Te2 | -42m | - | 3.629 | - | - | 0 | 3.023 | 3.022 | P-4m2 | 2OsTe-3 | No | 0.498 |
| Os2Te2 | -6m2 | - | 4.215 | - | - | - | 4.136 | 4.136 | P-6m2 | 2OsTe-5 | No | 0.773 |
| Ir2S2 | -3m | - | 4.607 | - | - | - | 3.248 | 3.248 | P-3m1 | 2IrS-5 | No | 0.337 |
| Ir2S2 | mm2 | - | 4.888 | - | - | - | 3.733 | 3.885 | Pmm2 | 2IrS-4 | Yes | 0.321 |
| Ir2S2 | 4/mmm | - | 4.142 | - | - | - | 3.873 | 3.873 | P4/nmm | 2IrS-6 | No | 0.374 |
| Ir2S2 | -3m | - | 4.817 | 0.412 | 0.631 | 0.219 | 3.279 | 3.28 | P-3m1 | 2IrS-1 | Yes | 0.158 |
| Ir2S2 | -6m2 | - | 4.763 | -0.043 | 0.669 | 0.713 | 3.236 | 3.236 | P-6m2 | 2IrS-3 | No | 0.305 |
| CdSe2 | -42m | - | 5.14 | - | - | 0 | 3.215 | 3.215 | P-4m2 | 1CdSe2-1 | No | 0.435 |
| CrSe2 | -42m | 2 | 5.13 | - | - | - | 3.778 | 3.778 | P-4m2 | 1CrSe2-3 | Yes | 0.285 |
| CrSe2 | -6m2 | - | 4.884 | -0.767 | 0.361 | 1.128 | 3.21 | 3.21 | P-6m2 | 1CrSe2-1 | Yes | 0 |
| CrSe2 | -3m | - | 4.615 | - | - | - | 3.223 | 3.223 | P-3m1 | 1CrSe2-2 | No | 0.14 |
| CuSe2 | -6m2 | - | 3.869 | - | - | - | 3.844 | 3.844 | P-6m2 | 1CuSe2-2 | Yes | 0.222 |
| CuSe2 | -42m | - | 5.158 | - | - | - | 2.865 | 2.865 | P-4m2 | 1CuSe2-1 | No | 0.128 |
| CuSe2 | -3m | - | 5.422 | - | - | - | 3.39 | 3.39 | P-3m1 | 1CuSe2-3 | Yes | 0.267 |
| HfSe2 | -42m | - | 5.259 | -3.827 | -1.193 | 2.634 | 4.01 | 4.01 | P-4m2 | 1HfSe2-3 | Yes | 0.337 |
| HfSe2 | -3m | - | 5.16 | -1.947 | -0.727 | 1.219 | 3.773 | 3.773 | P-3m1 | 1HfSe2-1 | Yes | 0 |
| HfSe2 | -6m2 | - | 5.859 | -2.689 | -1.063 | 1.626 | 3.677 | 3.677 | P-6m2 | 1HfSe2-2 | Yes | 0.174 |
| MoSe2 | -42m | 0.374 | 4.695 | - | - | - | 3.801 | 3.801 | P-4m2 | 1MoSe2-3 | No | 0.637 |
| MoSe2 | -3m | - | 4.563 | - | - | - | 3.282 | 3.282 | P-3m1 | 1MoSe2-2 | No | 0.234 |
| MoSe2 | -6m2 | - | 4.572 | -0.725 | 1.079 | 1.804 | 3.32 | 3.32 | P-6m2 | 1MoSe2-1 | Yes | 0 |
| NbS2 | -3m | - | 5.329 | - | - | - | 3.378 | 3.378 | P-3m1 | 1NbS2-2 | Yes | 0.033 |
| NbS2 | -6m2 | - | 6.096 | - | - | - | 3.344 | 3.344 | P-6m2 | 1NbS2-1 | No | 0 |
| NbS2 | -42m | - | 4.835 | - | - | - | 3.72 | 3.72 | P-4m2 | 1NbS2-3 | Yes | 0.406 |
| Os2Se4 | 4/mmm | - | 5.225 | -2.795 | -2.588 | 0.207 | 6.134 | 6.134 | P4/mbm | 2OsSe2-2 | No | 0.679 |
| Os2Se4 | 2/m | - | 4.524 | -1.188 | 0.201 | 1.389 | 3.666 | 5.763 | P2_1/m | 2OsSe2-1 | Yes | 0 |
| ReSe2 | -3m | - | 4.43 | - | - | - | 3.172 | 3.172 | P-3m1 | 1ReSe2-2 | No | 0.372 |
| ReSe2 | -6m2 | - | 3.93 | - | - | - | 3.456 | 3.456 | P-6m2 | 1ReSe2-1 | No | 0.308 |
| ReSe2 | -42m | - | 4.408 | - | - | - | 3.814 | 3.814 | P-4m2 | 1ReSe2-3 | No | 0.638 |
| RuS2 | -42m | - | 5.217 | - | - | - | 3.649 | 3.649 | P-4m2 | 1RuS2-3 | Yes | 0.484 |
| RuS2 | -3m | 1.842 | 5.847 | - | - | - | 3.354 | 3.354 | P-3m1 | 1RuS2-1 | No | 0.409 |
| RuS2 | -6m2 | - | 4.772 | - | - | - | 3.332 | 3.332 | P-6m2 | 1RuS2-2 | No | 0.435 |
| ScTe2 | -42m | - | 5.515 | - | - | - | 3.931 | 3.931 | P-4m2 | 1ScTe2-3 | No | 0.455 |
| ScTe2 | -3m | - | 5.552 | - | - | - | 3.828 | 3.828 | P-3m1 | 1ScTe2-1 | No | 0.153 |
| ScTe2 | -6m2 | - | 5.269 | - | - | - | 3.742 | 3.742 | P-6m2 | 1ScTe2-2 | No | 0.285 |
| VS2 | -42m | 1 | 5.56 | - | - | - | 3.59 | 3.59 | P-4m2 | 1VS2-3 | Yes | 0.246 |
| VS2 | -3m | 0.491 | 5.515 | - | - | - | 3.195 | 3.195 | P-3m1 | 1VS2-2 | Yes | 0.017 |
| VS2 | -6m2 | 0.953 | 5.914 | - | - | - | 3.186 | 3.186 | P-6m2 | 1VS2-1 | Yes | 0 |
| ZrS2 | -6m2 | - | 6.366 | -3.524 | -1.502 | 2.023 | 3.571 | 3.571 | P-6m2 | 1ZrS2-2 | No | 0.19 |
| ZrS2 | -42m | - | 5.723 | -4.624 | -1.523 | 3.101 | 3.915 | 3.915 | P-4m2 | 1ZrS2-3 | Yes | 0.31 |
| ZrS2 | -3m | - | 5.846 | -3.272 | -1.105 | 2.167 | 3.682 | 3.682 | P-3m1 | 1ZrS2-1 | Yes | 0 |
| Hf2S6 | mmm | - | 5.404 | -1.519 | 0.59 | 2.109 | 5.153 | 3.619 | Pmmn | 2HfS3-1 | Yes | 0 |
| Os2Te6 | 2/m | - | 4.605 | - | - | - | 6.172 | 3.76 | P2_1/m | 2OsTe3-1 | Yes | 0.248 |
| Ti2S6 | mmm | - | 5.238 | -0.778 | 0.674 | 1.452 | 5.031 | 3.419 | Pmmn | 2TiS3-1 | Yes | 0 |
| Au2Te2 | -3m | - | 4.736 | - | - | 0 | 4.218 | 4.219 | P-3m1 | 2AuTe-4 | No | 0.152 |
| Au2Te2 | -3m | - | 5.411 | - | - | - | 4.372 | 4.372 | P-3m1 | 2AuTe-6 | No | 0.264 |
| Au2Te2 | -6m2 | - | 4.084 | -0.706 | -0.628 | 0.078 | 4.682 | 4.682 | P-6m2 | 2AuTe-3 | Yes | 0.139 |
| Au2Te2 | 2/m | - | 4.548 | -2.69 | -1.623 | 1.067 | 4.015 | 6.627 | P2/m | 2AuTe-1 | Yes | 0 |
| HgS | 3m | - | 5.44 | -4.01 | -3.286 | 0.724 | 4.287 | 4.287 | P3m1 | 1HgS-1 | No | 0.146 |
| Cd2Te4 | 2/m | - | 4.111 | -2.238 | -0.474 | 1.764 | 5.973 | 6.472 | C2/m | 2CdTe2-1 | No | 0.244 |
| Ni2Se4 | 2/m | - | 4.686 | -3.849 | -1.666 | 2.183 | 5.509 | 5.704 | P2_1/c | 2NiSe2-1 | Yes | 0.067 |
| Fe2Te6 | 2/m | - | 4.665 | - | - | - | 6.146 | 3.488 | P2_1/m | 2FeTe3-2 | No | 0.215 |
| Hf2Se6 | mmm | - | 5.025 | -0.464 | 0.421 | 0.885 | 5.452 | 3.751 | Pmmn | 2HfSe3-1 | Yes | 0.003 |
| Ir2S6 | m | - | 5.528 | - | - | - | 5.773 | 3.597 | Pc | 2IrS3-1 | Yes | 0.213 |
| Ti2Se6 | mmm | - | 4.85 | 0.108 | 0.428 | 0.32 | 5.351 | 3.562 | Pmmn | 2TiSe3-1 | No | 0.018 |
| V2S6 | mmm | - | 4.484 | - | - | - | 4.878 | 3.243 | Pmmn | 2VS3-1 | No | 0.088 |
| Hg2Se2 | -3m | - | 5.646 | -2.746 | -2.457 | 0.289 | 4.478 | 4.479 | P-3m1 | 2HgSe-1 | No | 0.107 |
| Mn2S2 | -3m | 1.164 | 5.061 | - | - | - | 3.534 | 3.534 | P-3m1 | 2MnS-7 | Unknown | 0.314 |
| CdTe2 | -42m | - | 4.439 | - | - | - | 3.5 | 3.5 | P-4m2 | 1CdTe2-1 | No | 0.286 |
| NiSe2 | -42m | 0.974 | 4.958 | - | - | - | 2.953 | 2.953 | P-4m2 | 1NiSe2-3 | No | 0.293 |
| NiSe2 | -6m2 | - | 4.39 | - | - | - | 3.507 | 3.507 | P-6m2 | 1NiSe2-2 | Yes | 0.18 |
| NiSe2 | -3m | - | 4.846 | -1.632 | -1.333 | 0.299 | 3.542 | 3.542 | P-3m1 | 1NiSe2-1 | Yes | 0 |
| OsSe2 | -3m | - | 4.966 | - | - | - | 3.661 | 3.661 | P-3m1 | 1OsSe2-1 | No | 0.294 |
| OsSe2 | -6m2 | - | 4.064 | - | - | - | 3.513 | 3.513 | P-6m2 | 1OsSe2-2 | No | 0.322 |
| OsSe2 | -42m | - | 4.474 | - | - | - | 3.079 | 3.079 | P-4m2 | 1OsSe2-3 | No | 0.41 |
| TiTe2 | -6m2 | - | 5.15 | -1.029 | -0.307 | 0.722 | 3.737 | 3.737 | P-6m2 | 1TiTe2-2 | Yes | 0.109 |
| TiTe2 | -42m | - | 4.959 | -2.625 | -1.157 | 1.469 | 4.123 | 4.123 | P-4m2 | 1TiTe2-3 | Yes | 0.338 |
| TiTe2 | -3m | - | 4.803 | - | - | - | 3.744 | 3.744 | P-3m1 | 1TiTe2-1 | Yes | 0.009 |
| Zn2S4 | 2/m | - | 4.163 | -3.432 | -0.712 | 2.719 | 5.817 | 5.504 | P2_1/c | 2ZnS2-1 | Yes | 0.361 |
| Hf2Te6 | mmm | - | 4.818 | - | - | - | 5.955 | 3.939 | Pmmn | 2HfTe3-1 | Yes | 0.024 |
| Ir2Se6 | 2/m | - | 4.819 | - | - | - | 5.495 | 3.543 | P2_1/m | 2IrSe3-2 | Yes | 0.275 |
| Ti2Te6 | mmm | - | 4.696 | - | - | - | 5.928 | 3.748 | Pmmn | 2TiTe3-1 | Yes | 0.034 |
| V2Se6 | mmm | - | 4.657 | - | - | - | 5.228 | 3.376 | Pmmn | 2VSe3-1 | No | 0.092 |
| Mn2Se2 | 4/mmm | 2.735 | 4.44 | - | - | - | 3.594 | 3.594 | P4/nmm | 2MnSe-1 | Yes | 0.038 |
| Mn2Se2 | mmm | 0.913 | 4.675 | - | - | - | 3.555 | 3.749 | Pmmm | 2MnSe-2 | No | 0.086 |
| Ag2S2 | -3m | - | 5.738 | - | - | - | 4.181 | 4.186 | P-3m1 | 2AgS-3 | Yes | 0.289 |
| Ag2S2 | 2/m | - | 5.718 | -4.495 | -2.938 | 1.557 | 3.567 | 6.106 | P2/m | 2AgS-1 | Yes | 0.12 |
| HgSe | 3m | - | 5.427 | -3.869 | -3.445 | 0.423 | 4.464 | 4.464 | P3m1 | 1HgSe-1 | Yes | 0.157 |
| ZnS2 | -42m | - | 5.824 | - | - | - | 2.984 | 2.984 | P-4m2 | 1ZnS2-1 | No | 0.609 |
| Cr2Te6 | 2/m | 4.071 | 4.829 | - | - | - | 6.175 | 3.619 | P2_1/m | 2CrTe3-1 | No | 0.099 |
| Ir2Te6 | 2/m | - | 4.481 | - | - | - | 6.178 | 3.77 | P2_1/m | 2IrTe3-2 | Yes | 0.232 |
| V2Te6 | 2/m | 1.235 | 4.705 | - | - | - | 6.152 | 3.565 | P2_1/m | 2VTe3-1 | Yes | 0.067 |
| YS2 | -42m | 1 | 6.881 | - | - | - | 4.275 | 4.275 | P-4m2 | 1YS2-1 | Unknown | 0.8 |
| Mn2Te2 | 4/mmm | 0.266 | 4.203 | - | - | - | 3.773 | 3.827 | P4/mmm | 2MnTe-2 | No | 0.121 |
| Rh4Se8 | -1 | - | 5.199 | -2.218 | -1.172 | 1.046 | 6.639 | 7.387 | P-1 | 4RhSe2-1 | Yes | 0.034 |
| ReS2 | -6m2 | - | 4.205 | - | - | - | 3.307 | 3.307 | P-6m2 | 1ReS2-1 | No | 0.316 |
| NiS2 | -3m | - | 5.5 | -2.698 | -1.696 | 1.002 | 3.354 | 3.354 | P-3m1 | 1NiS2-1 | Yes | 0.049 |
| Ru2Se4 | 2/m | - | 4.727 | -1.779 | -0.184 | 1.595 | 3.595 | 5.792 | P2_1/m | 2RuSe2-1 | Yes | 0.076 |
| Rh2Se4 | 2/m | - | 5.339 | - | - | 0 | 5.992 | 6.039 | P2_1/c | 2RhSe2-2 | No | 0.34 |

Table S3. 2D transition metal halides with a notable magnetic moment (>1 μB), structural stability, and a band gap of 0.5–2.5 eV.

| Formula | Point group | Magnetic moment | Work fuction | vbm | cbm | band_gap | a_length | b_length | space_group | uid | Dynamically stable | Energy above hull |
| --- | --- | --- | --- | --- | --- | --- | --- | --- | --- | --- | --- | --- |
| NiI2 | -3m | 1.998 | 4.881 | -2.386 | -0.482 | 1.904 | 3.97 | 3.97 | P-3m1 | 1NiI2-1 | Yes | 0 |
| Au2Cl6 | -3m | 3.92 | 6.589 | -4.815 | -3.539 | 1.276 | 6.505 | 6.505 | P-31m | 2AuCl3-1 | Yes | 0.169 |
| Cu2Br6 | -3m | 3.899 | 6.416 | -4.448 | -3.805 | 0.643 | 6.481 | 6.481 | P-31m | 2CuBr3-1 | Yes | 0.091 |
| Mn2Cl6 | -3m | 4.009 | 5.317 | -2.245 | -1.366 | 0.879 | 5.901 | 5.901 | P-31m | 2MnCl3-2 | Yes | 0.073 |
| PdBr2 | -3m | 1.981 | 5.577 | -3.642 | -2.117 | 1.525 | 3.904 | 3.904 | P-3m1 | 1PdBr2-1 | Yes | 0.148 |
| Cu2Cl6 | -3m | 3.985 | 7.168 | -5.65 | -4.241 | 1.409 | 6.107 | 6.107 | P-31m | 2CuCl3-1 | Yes | 0.072 |
| ScBr2 | -6m2 | 0.985 | 3.846 | -0.367 | 0.39 | 0.757 | 3.694 | 3.694 | P-6m2 | 1ScBr2-1 | Yes | 0 |
| YBr2 | -6m2 | 0.997 | 3.762 | -0.554 | 0.347 | 0.901 | 3.867 | 3.867 | P-6m2 | 1YBr2-1 | Yes | 0.016 |
| W2I6 | -3m | 6 | 4.368 | -2.088 | -0.058 | 2.03 | 7.201 | 7.201 | P-31m | 2WI3-2 | Yes | 0.069 |
| PdCl2 | -3m | 1.998 | 6.009 | -4.339 | -2.254 | 2.086 | 3.731 | 3.731 | P-3m1 | 1PdCl2-1 | Yes | 0.166 |
| Ti2Br6 | -6m2 | 2 | 4.819 | -2.289 | -1.217 | 1.071 | 6.594 | 6.594 | P-62m | 2TiBr3-2 | Yes | 0.065 |
| YCl2 | -6m2 | 0.998 | 3.901 | -0.746 | 0.253 | 0.999 | 3.73 | 3.73 | P-6m2 | 1YCl2-1 | Yes | 0.046 |
| ScCl2 | -6m2 | 0.981 | 4.057 | -0.521 | 0.215 | 0.736 | 3.518 | 3.518 | P-6m2 | 1ScCl2-1 | Yes | 0.009 |
| Cu2F4 | 2/m | 2 | 7.325 | -8.832 | -8.235 | 0.596 | 3.416 | 5.28 | P2_1/c | 2CuF2-1 | Yes | 0.011 |
| Ti2I6 | -6m2 | 2 | 4.668 | -1.85 | -0.709 | 1.141 | 7.154 | 7.154 | P-62m | 2TiI3-2 | Yes | 0.083 |
| Ag2Br6 | -3m | 3.733 | 6.522 | -4.852 | -4.084 | 0.768 | 6.843 | 6.843 | P-31m | 2AgBr3-5 | Yes | 0.131 |
| ScI2 | -6m2 | 0.971 | 3.688 | 0.227 | 0.8 | 0.573 | 3.979 | 3.979 | P-6m2 | 1ScI2-2 | Yes | 0.008 |
| YI2 | -6m2 | 0.981 | 3.583 | 0.171 | 0.715 | 0.544 | 4.105 | 4.105 | P-6m2 | 1YI2-1 | Yes | 0.003 |
| Ag2Cl6 | -3m | 3.919 | 7.184 | -5.778 | -4.5 | 1.278 | 6.476 | 6.476 | P-31m | 2AgCl3-1 | Yes | 0.123 |
| Cr2I6 | -3m | 6 | 5.009 | -2.86 | -0.769 | 2.091 | 7.008 | 7.008 | P-31m | 2CrI3-1 | Yes | 0 |
| Au2Br6 | -3m | 3.717 | 6.086 | -4.129 | -3.269 | 0.86 | 6.847 | 6.847 | P-31m | 2AuBr3-2 | Yes | 0.121 |

**DFT Calculations:**


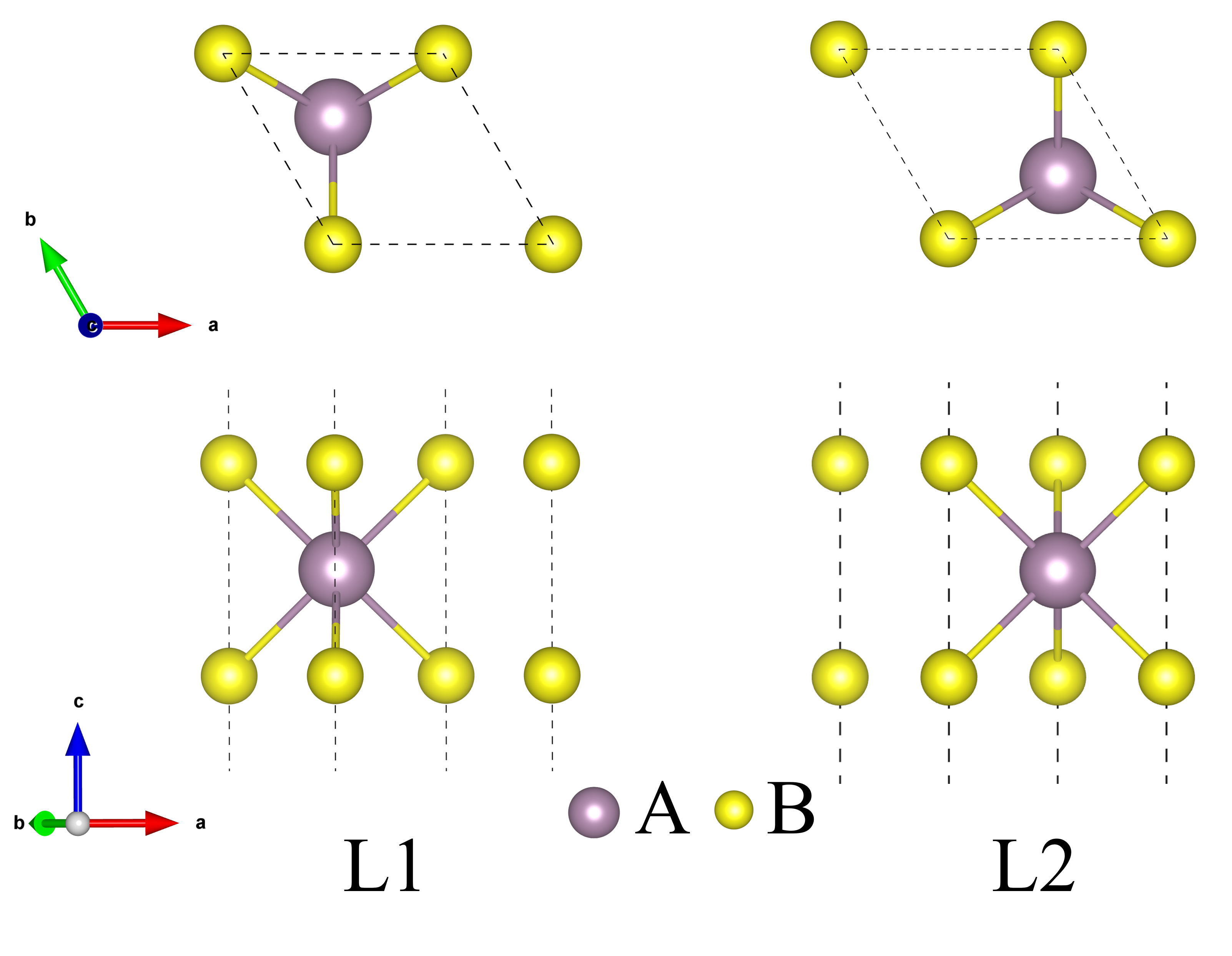


Fig S1. Geometric structures of AB_2_ (A = Sc, Y, B = Cl, Br, I) monolayer for the fractional quantum ferroelectric L1 and L2 phases.


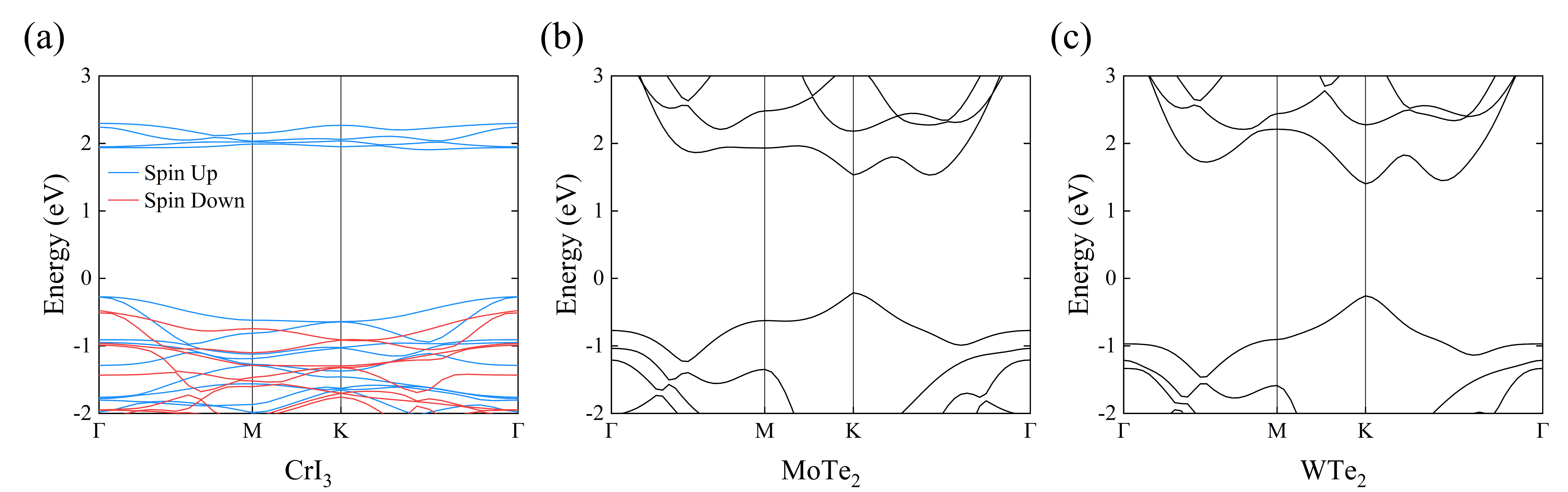


Fig S2. The band structures of single-layer CrI_3_, MoTe_2_, and WTe_2_.


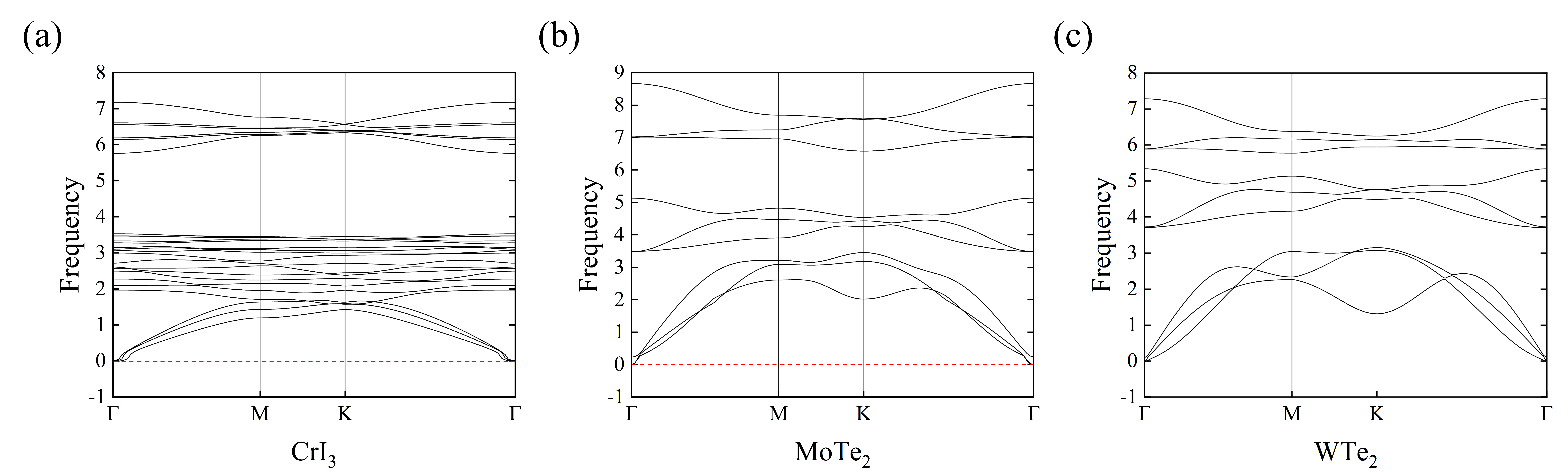


Fig S3. Phonon dispersion curves of CrI_3_, MoTe_2_ and WTe_2_ monolayers.

Fig S4. The projected band structure of stacking configurations for CrI_3_/MoTe_2_ HJs: (a) stacking-1, (b) stacking-2, and (c) stacking-3; and for CrI_3_/WTe_2_ HJs: (f) stacking-4, (g) stacking-5, and (h) stacking-6. The projected band structures of non-polarized CrI_3_/MoTe_2_ HJ (d) and CrI_3_/WTe_2_ HJ (h).


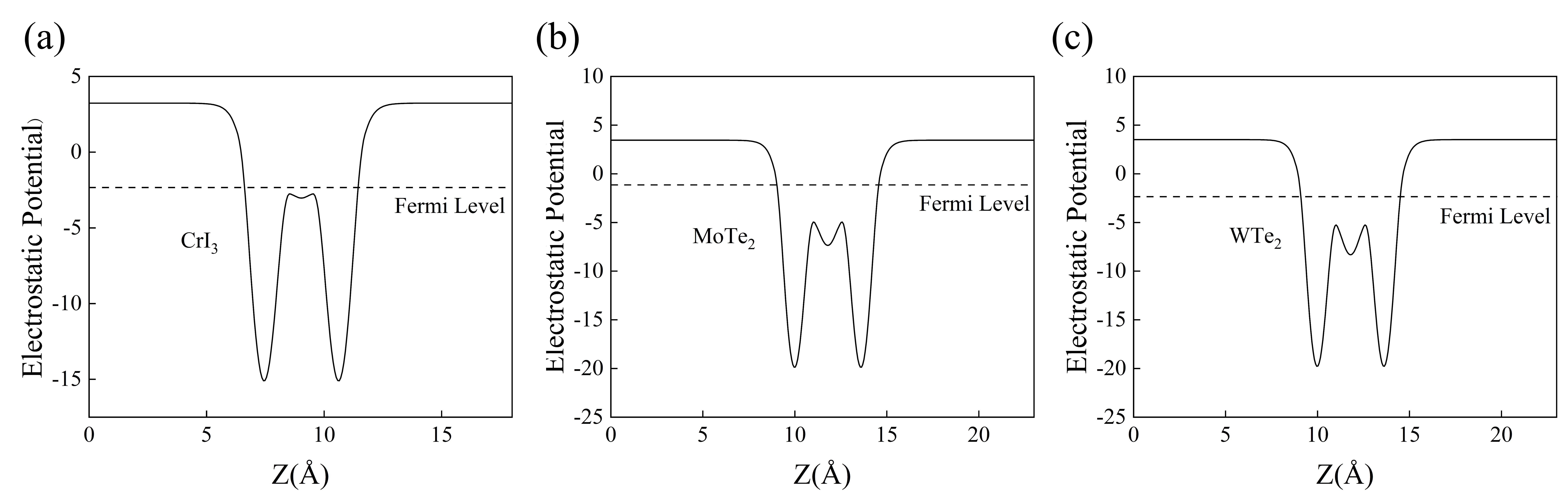


Fig ~~S4~~S5. Electrostatic potential distributions of CrI_3_, MoTe_2_ and WTe_2_ monolayers.


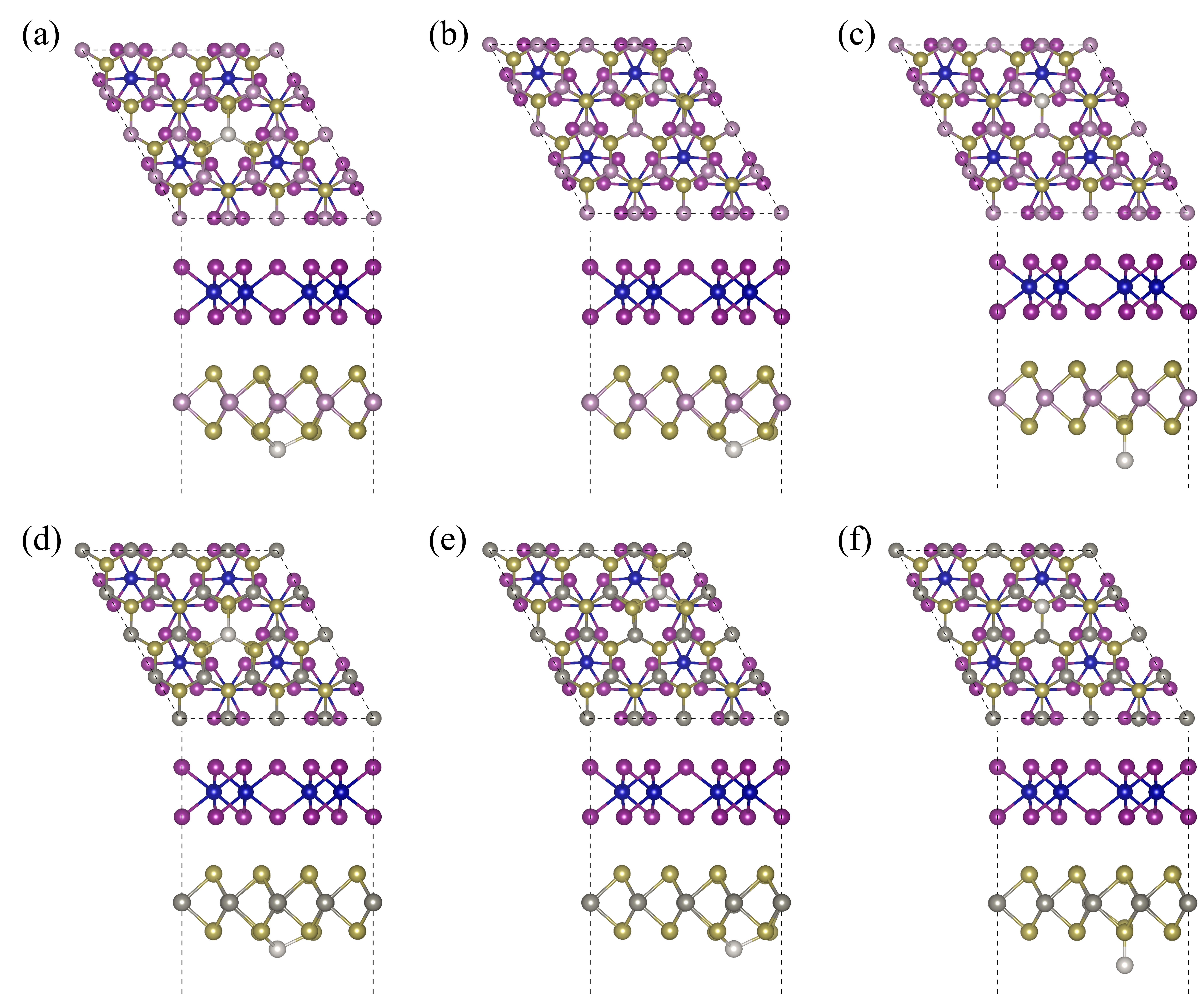


Fig ~~S5~~ S6. Anchoring configurations of single Pt atom on CrI_3_/MoTe_2_ and CrI_3_/WTe_2_ HJs.


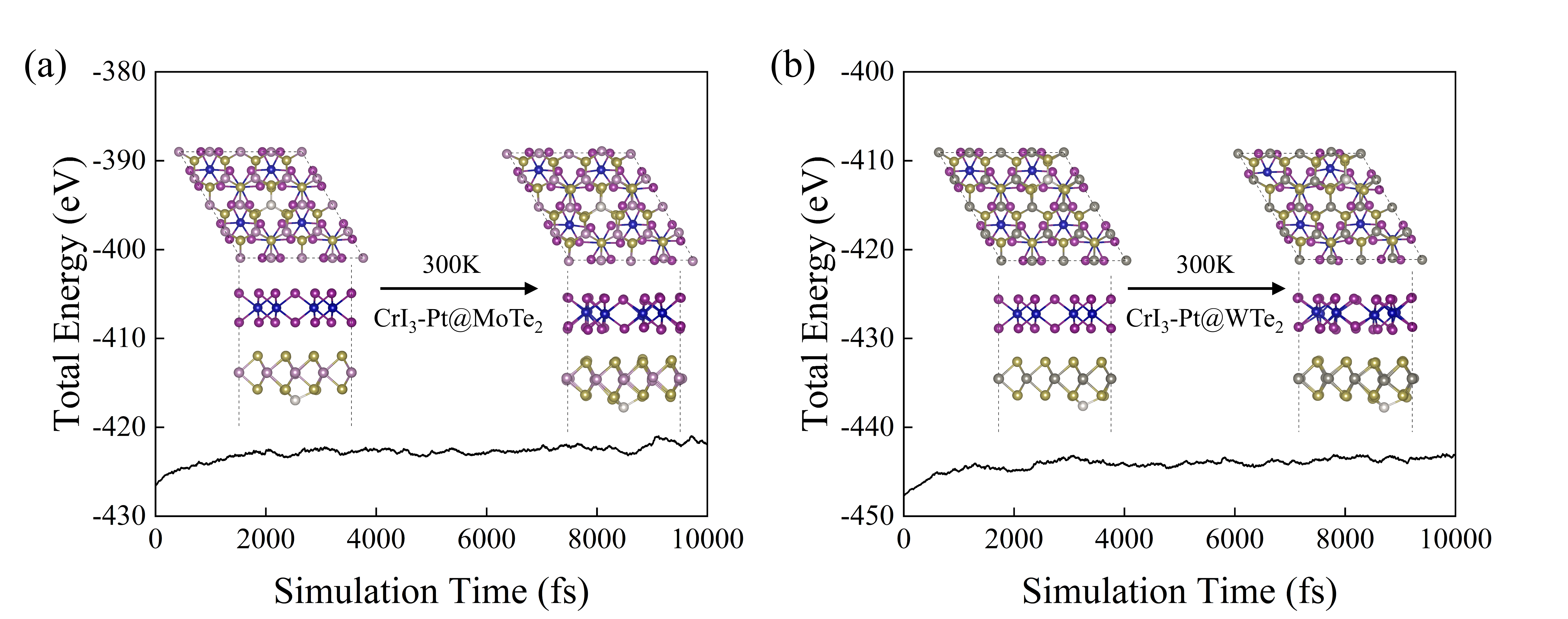


Fig S7. Ab initio molecular dynamics simulation results at 300 K are presented for (a) CrI_3_/Pt@MoTe_2_ and (b) CrI_3_/Pt@WTe_2_ heterojunctions.


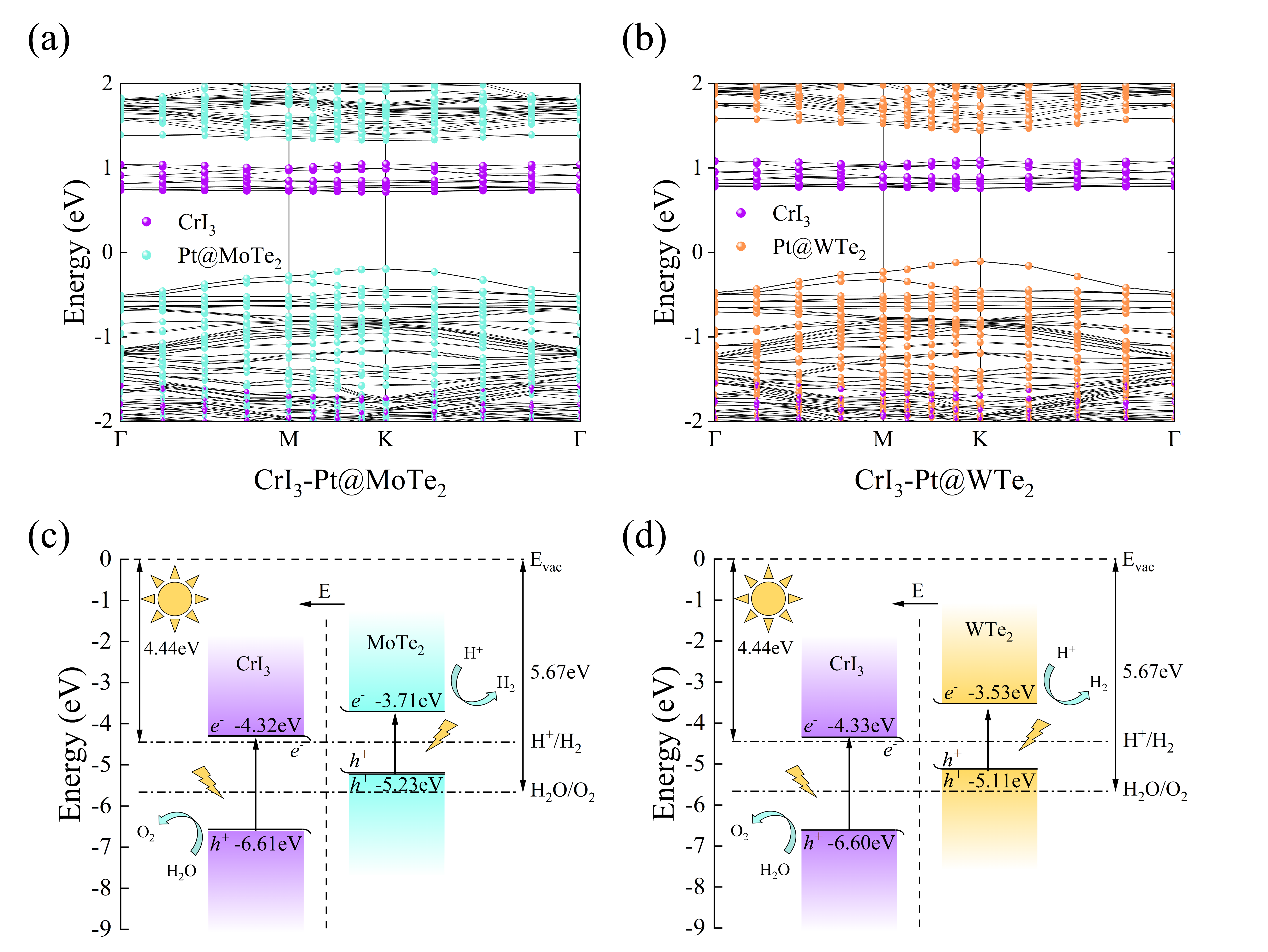


Fig ~~S6~~ S8. The band structures of CrI_3_/MoTe_2_ and CrI_3_/WTe_2_ HJs anchored with single Pt atoms.
